# Statistical Analysis of Copper(I) Iodide and Bis(Diphenylphosphino)alkane-Based Complexes and Coordination Polymers

**DOI:** 10.3390/molecules28237781

**Published:** 2023-11-25

**Authors:** Léo Boivin, Adrien Schlachter, Daniel Fortin, Christophe Lescop, Pierre D. Harvey

**Affiliations:** 1 Département de Chimie, Université de Sherbrooke, Sherbrooke, QC J1K 2R1, Canada; 2 ISCR (Institut des Sciences Chimiques de Rennes)—UMR 6226, CNRS, INSA Rennes, University Rennes, F-35000 Rennes, France

**Keywords:** copper(I) iodide coordination polymers, photophysics, TADF

## Abstract

The prediction of the metal cluster within a coordination polymer or complex, as well as the dimensionality of the resulting polymer or complex (i.e., 0D, 1D, 2D, or 3D), is often challenging. This is the case for Ph_2_P(CH_2_)_m_PPh_2_ ligands (1 ≤ m ≤ 8) and CuX salts, particularly for X = I. This work endeavors a systematic statistical analysis combining studies in the literature and new data, mapping the nature of the resulting CuI aggregates with eight different diphoshphines in 2:1, 3:2, 1:1, 2:3, and 1:2 CuI:Ph_2_P(CH_2_)_m_PPh_2_ molar ratios as a function of m, which lead to either pure products or mixtures. Several trends are made relating stoichiometry and chain length to the CuI cluster formed (i.e., globular vs. quasi-planar). Four new X-ray structures were determined: [Cu_3_I_2_(**L1**)_3_]I, Cu_3_I_3_(**L2**)_2_, Cu_2_I_2_(**L6**)_2_, and Cu_4_I_4_(**L8**)_2_, where m is, respectively, 1, 2, 6, and 8, in which the Cu_x_I_y_ central aggregates adopt triangular bipyramid, diamond, rhomboid, and cubane shaped motifs, respectively. Photophysical measurements assisted the establishment of trends considering the paucity of the crystallographic structures. During this study, it was also found that the 0D-complex Cu_2_I_2_(Ph_2_P(CH_2_)_5_PPh_2_)_2_ exhibits thermally activated delayed fluorescence.

## 1. Introduction

The quest to predict the resulting identity of complexes or coordination polymers (CPs) formed during the reaction between low-valent metals and soft bidentate ligands is, unfortunately, stubbornly futile. The ubiquitous structure–property relationship is of utmost importance when it comes to the time at which to aim specific applications, such as catalysis, medical, and photonic applications, since these features are directly linked to the nature of the central resulting metal aggregates or nodes, and the constraints, both rigidity and steric hindrance, imposed by the ligands [1,2]. For instance, the properties and applications of (di)chalcogenoether and (di)chalcogenone complexes and CPs of copper(I) halide salts have recently been reviewed, and the main conclusion is that 0D- (distinct complexes), 1D-, 2D-, and 3D-coordination polymers (CPs) are formed in an unpredictable manner, with a clear statistical preference for 1D- and 2D-CPs [1,2].

These observations are essentially based on probability. However, good practice states that predictions should be performed using comparables with a minimum number of variables to extract reliable cause-and-effect relationships.

**Figure 1:**
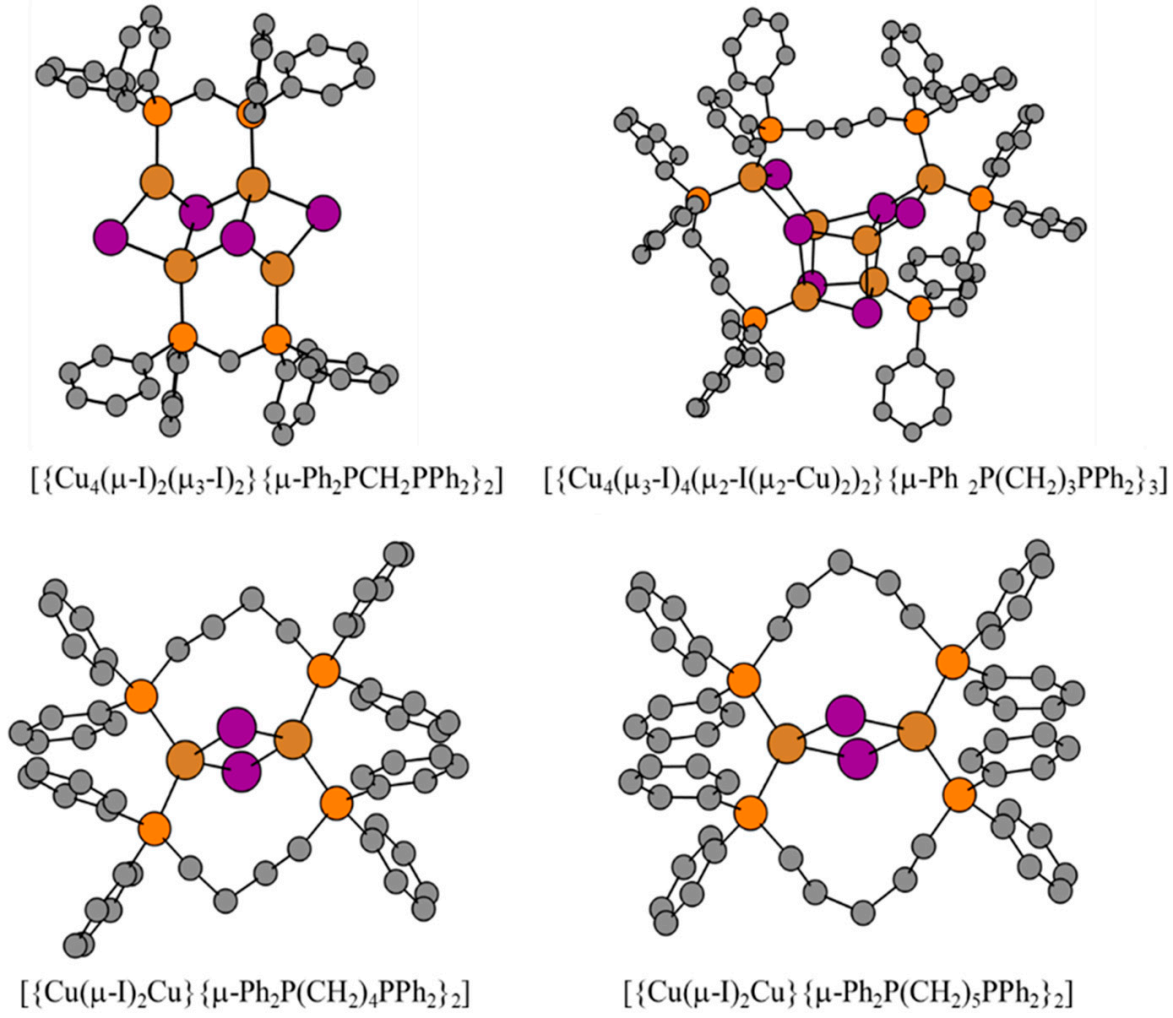
Selected examples of known (CuI)_n_/diphosphine complexes: (**top**) step staircase [3,4] eared cubane [8], and (**bottom**) rhomboid (C4 and C5 chains) [9]. Gray: carbon, orange: phosphorus, brown: copper, purple: iodine.

Diphosphines, namely of the type Ph_2_P(CH_2_)_m_PPh_2_ (Ph = phenyl), are an important class of ligands to the point that many are even available commercially (1 ≤ m ≤ 6, m = 8). Their reactivity towards CuI salts has also been explored for m = 1 [3,4,5], 2 [6,7], 3 [8,9,10], 4 [9,11], and 5 [9], and the main observation is that this brochette of complexes is dominated by 0D- species, except, to the best of our knowledge, in one case (m = 3), where a 1D-CP has been reported [12]. This outcome drastically contrasts with that of other reported ligand classes, and the obvious question is why there is a such a drastic contrast. A large variation in the secondary binding unit (SBU) of these complexes can also be found (Figure 1), with as of yet very little predictability.

From the reported literature only, it is impossible to extract statistical trends in the formation of specific SBUs from the reaction conditions. This is due to the overwhelming paucity in crystallographic and photophysical data. However, a firm preference for the rhomboid SBU is found, especially for the 1:1 metal salt–ligand ratio, and 2 ≤ m ≤ 5 ligands [6,9,10,13,14]. The apparent predominance of globular (i.e., cubane, eared-cubane, diamond) motifs for ligand-poor reaction mixtures [6,7,11], and of CuI centers for ligand-rich mixtures [5,8,12], is confirmed in this work.

We now report a systematic investigation where the stoichiometry of the reaction CuI vs. diphosphine varies as 2:1, 3:2, 1:1, 2:3, and 1:2 for m = 1 to 8 inclusively (i.e., 40 reaction mixtures). The presence of unidentified oligomers, for most cases, strongly interferes with the purity and the uniqueness of the resulting products, where only seven out of forty reactions give rise to a pure product.

Four new structures, two of which exhibit new motifs for this category of complexes, were determined using a single crystal X-ray diffraction to increase the current data bank of 10 literature structures. The structure for the Cu_4_I_4_(**L8**)_2_ product is notable in the sense that it is the first reported structure for such bis(diphenyl)phosphinoalkane ligands to exhibit a cubane SBU. Moreover, an unusual (but not unheard of) motif ([triangular bipyramid]^+^) is found for the [Cu_3_I_2_(**L1**)_3_]I complex. The addition of the structures of Cu_2_I_2_(**L6**)_2_ (rhomboid SBU) and Cu_4_I_4_(**L8**)_2_ ends the heretofore inconvenient paucity of crystallographic structures for these complexes with m > 5. Finally, the Cu_3_I_3_(**L2**)_2_ structure helps in the precision of the stoichiometric dependency of the SBU.

A full photophysical characterization of all 40 reaction mixtures was performed to link central CuI aggregate motifs with spectral and emission lifetime signatures, and therefore expand the statistical trends reported herein by addressing the crystallographic paucity. New CuI cluster formation trends concerning stoichiometry and chain length can be extracted for the CuI-diphosphine complexes.

## 2. Results

A code system is used to simplify the reading. In essence, ligands and complexes are respectively coded **Lm** and **m(x:y)** where m is the number of CH_2_ in the chain and x:y is the molar stoichiometry ratio used for the reaction CuI:diphosphine ligand.

### 2.1. Synthesis and Powder X-ray Diffraction (PXRD)

**Scheme 1:**
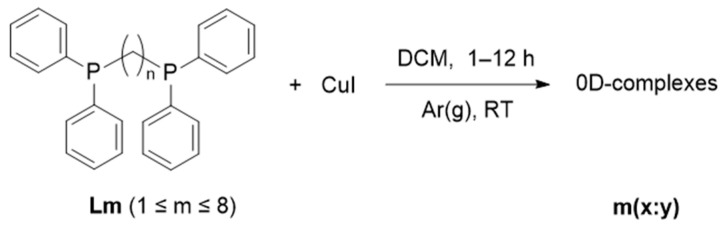
General reaction scheme for the preparation of all complexes.

The general reaction is shown in Scheme 1, where dichloromethane was used to solubilize the diphosphine ligands.

**Figure 2:**
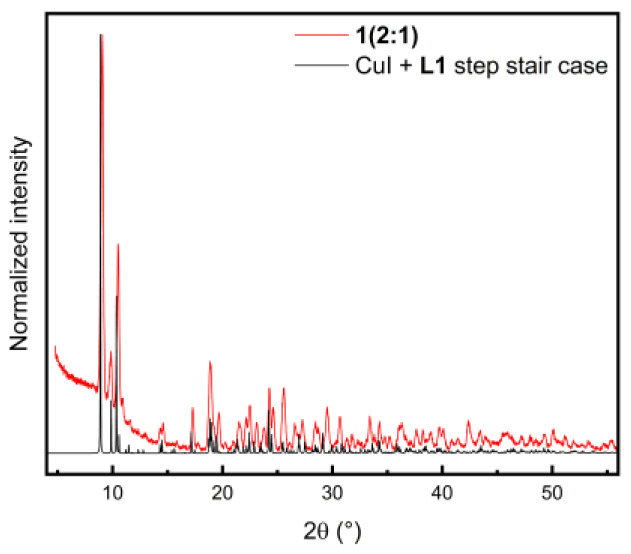
PXRD pattern of **1(2:1)**, compared to the calculated pattern for the step staircase motif published in references [3,4].

**Figure 3:**
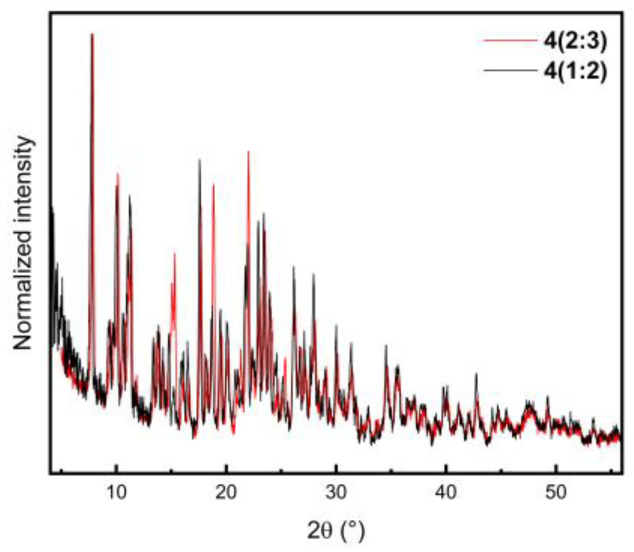
Peak matching between the PXRD patterns of **4(2:3)** and **4(1:2)**.

**Figure 4:**
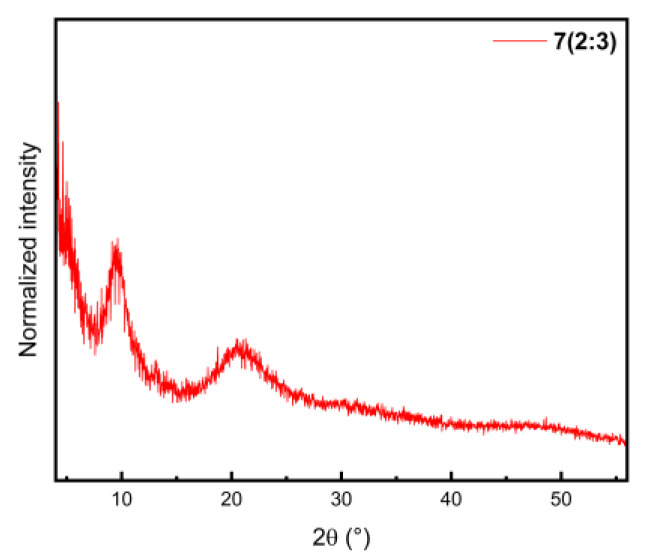
PXRD pattern of **7(2:3)** showing an amorphous halo.

The PXRD pattern of each reaction mixture was acquired. Three typical situations occur and are now described. The remainder patterns are placed in the Supplementary Material (Figures S1–S40). The first example concerns the case where the PXRD pattern corresponds to that of another reported pattern, or a theoretical pattern calculated from a deposited *.cif file. This is precisely the case for **1(2:1)** (Figure 2). In this case, the PXRD experiment compares favorably to that calculated for the step staircase motif presented in Figure 1 (top left) [3,4]. Another case of such a situation is occasionally observed when the stoichiometry of the reaction varies but the major product is the same. This is the case for the reaction mixtures **4(2:3)** and **4(1:2)** for the known product (chelate-**L4**)CuI(bridging-**L4**)CuI(chelate-**L4**) (Figure 3). [11] In brief, the identity and relative purity of a sample can readily be assessed. The second example concerns the case where the PXRD pattern exhibits large halos, indicating that the sample is amorphous with no crystalline domain. The impossibility to crystallize a sample is often due to a mixture of ill-defined species (like poorly soluble poly-dispersed oligomers). This is the case for the reaction mixture **7(2:3)** (Figure 4). It is also noteworthy that, despite a great effort, all attempts to obtain crystals suitable for X-ray crystallography for any reaction mixture containing **L7** stubbornly failed. Another situation concerns a pattern exhibiting a mixture of sharp diffraction peaks (similar to those presented in Figure 2 and Figure 3) along with halos (as exemplified in Figure 4), and is indicative of amorphous and crystalline domains, most likely related to a mixture of products.

### 2.2. Single Crystal X-ray Diffraction

**Figure 5:**
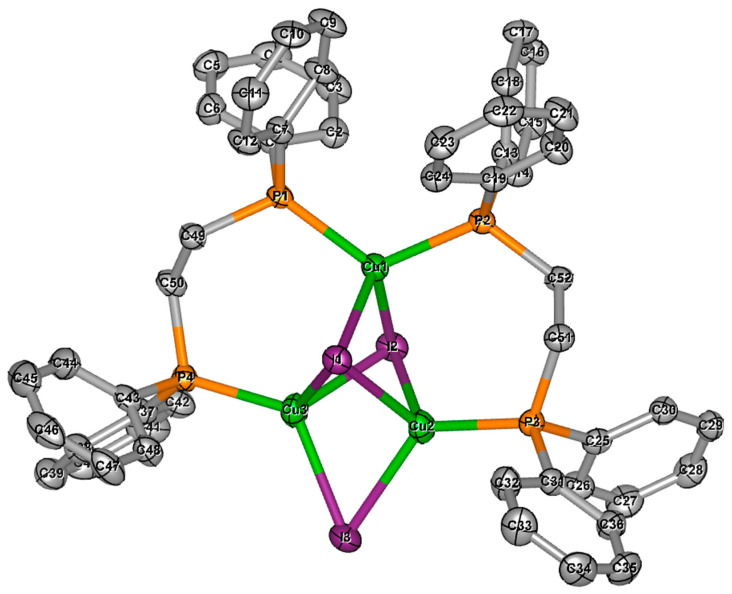
Molecular structure of **2(2:1)**. Molecular structures of propionitrile solvent molecules and H-atoms omitted. Anisotropic displacement ellipsoids are drawn at 50% probability level.

**Figure 6:**
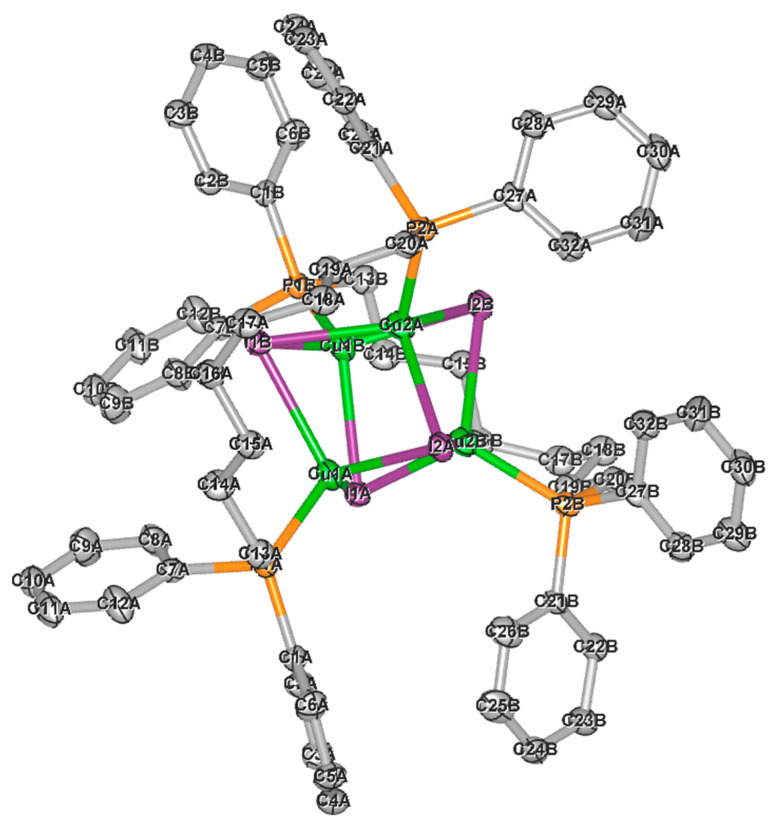
Molecular structure of **8(2:1)**. H-atoms omitted. Anisotropic displacement ellipsoids are drawn at 50% probability level.

**Figure 7:**
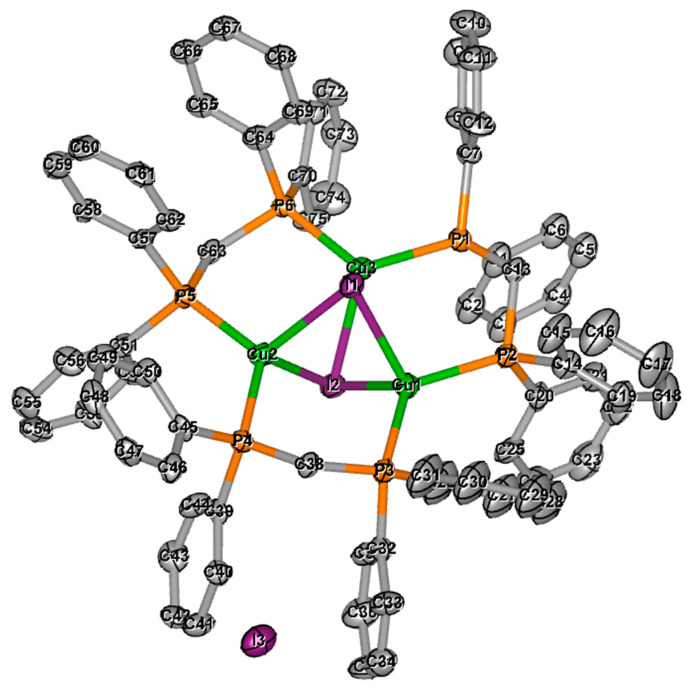
Molecular structure of **1(1:1)**. Molecular structures of chloroform solvent molecules and H-atoms omitted. Anisotropic displacement ellipsoids are drawn at 50% probability level.

**Figure 8:**
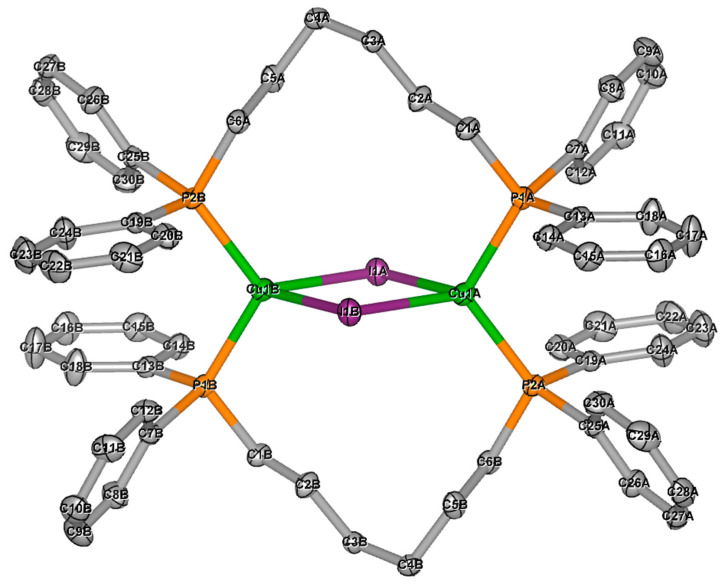
Molecular structure of **6(1:1)**. H-atoms omitted. Anisotropic displacement ellipsoids are drawn at 50% probability level.

Despite that 40 reaction mixtures were attempted, success in obtaining single crystals suitable for the SCXRD turned out to be poor, thus explaining the relative paucity of the X-ray structures available throughout the literature (to the best of our knowledge 10, as mentionned above). In this work, an X-ray analysis permitted us to determine four new structures for the products issued from **2(2:1)**, **8(2:1)**, **6(1:1)** and **1(1:1)** (Figure 5, Figure 6, Figure 7 and Figure 8; crystallographic data in Table 1). The metal aggregates are of a globular shape and adopt, respectively, a diamond (Cu_3_I_3_), cubane (Cu_4_I_4_), and triangular bipyramid ([Cu_3_I_2_]^+^) geometry. A supplementary rhomboid (quasi-planar) motif is also found for **6(1:1)**. The cubane and triangular bipyramid motifs are not uncommon, but turn out to be unprecedented for this series of 0D-CuI/Ph_2_P(CH_2_)_m_PPh_2_ complexes. However, the cubane geometry was also formed for a previously reported 1D-CP (**3(2:1)**). It is noteworthy that this CP was obtained in different solvent and temperature conditions (MeCN/H_2_O, 1:1 vs. CH_2_Cl_2_).

The Cu···Cu distances of the three new structures presented in Figure 5, Figure 6, Figure 7 and Figure 8 reveal three distances in the cubane motif that are near the sum of the Van der Waals radii of copper (2.8 Å), indicating very weak Cu···Cu interactions (Table 2) [1]. One distance at 2.522 Å is also depicted for the diamond central unit for the Cu-Cu bond tightly bridged by a µ_2_-iodide. In this case, significant Cu···Cu (cuprophilic) interactions are suspected. The other bond lengths, Cu-I and Cu-P, and the bond angles, are found to correspond to previously led investigations on similar complexes [3,4,5,6,7,8,9,10,11,12,13,14].

The structure for **8(2:1)** confirms that globular, or cubane-derived, motifs are preferred for the 2:1 ratio by joining the previously reported (1D-CP) cubane and eared-cubane motifs [8,12]. This is also true of **2(2:1)**’s diamond-shaped cluster. However, this substance is the first instance of such a motif in the 2:1 stoichiometry, as the only other reported instance (with **L1**) is made from a 3:2 stoichiometry [5]. Based on the literature, **6(1:1)**’s rhomboid motif was highly suspected, as there is a clear trend in rhomboid preference for this stoichiometric ratio (evidenced by the undisturbed line from m = 2 to m = 5, inclusively) [6,9,10,13,14]. This trend is broken, however, by **1(1:1)**’s peculiar (but not unheard of) [triangular bipyramid]^+^ motif.

Using PXRD and SCXRD analyses with reliable identifications, along with those reported in the literature (SCXRD), the current state of the art is summarized in Table 3. However, there are many gaps in this Table, where the reaction mixture obtained could not be identified reliably (Figures S1–S40).

It is noteworthy that the presence of excess γ-CuI particles has been observed based on the characteristic PXRD peaks. From a photophysical standpoint, γ-CuI is non-emissive and will not affect the luminescence band shape of aggregates containing saturated Cu-centers. This is the case for several reactions in Table 4.

### 2.3. Steady-State UV-Vis Spectroscopy

Single crystals suitable for X-ray structure determination were not obtained for all the reaction mixtures attempted. In previous studies on CuI/dithioether-containing CPs [1,2], there was a clear distinction between the photoluminescence arising from globular versus quasi-planar clusters. The former motifs (cubanes, fused dicubanes, etc.) tend to emit intensively, and the emission bands are generally red-shifted towards the near-IR. Conversely, the latter type of motifs (rhomboids, step staircases, etc.) exhibit a blue-shifted triplet emission, and the intensity of the luminescence tends to be highly variable, most of the time being silent. Based on this past observation, a trend was sought herein (globular motifs = cubane, eared cubane, diamond, and triangular bipyramid; quasi-planar = rhomboid, CuI, and step staircase) to use these signatures as a prediction tool (i.e., marker) for what may be present in some of the mixtures.

**Figure 9:**
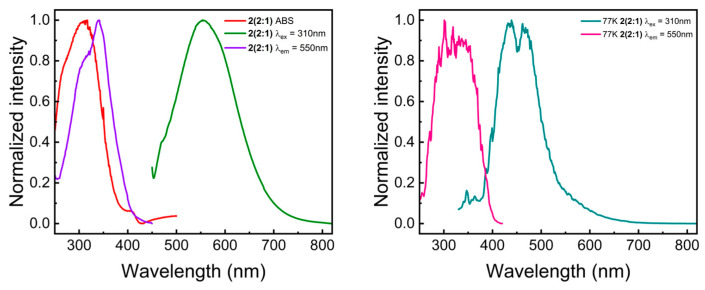
UV-Vis absorption (red), excitation (purple and pink), and photoluminescence (green and turquoise) spectra of Cu_3_I_3_(**L2**)_2_ (diamond motif; Figure 5 issued from the reaction **2(2:1)**; (**left**): 293 K. (**right**): 77 K.

Typical examples of absorption, emission, and excitation spectra are presented in Figure 9. At room temperature (left), the position of the emission band (λ_em_ = 555 nm) is red-shifted, meaning that the CuI-aggregate is of a globular shape, which is indeed verified using SCXRD (vide supra) as a diamond shape (Figure 5). A weak shoulder in the 450–500 nm range is noticed. The assignment for the 555 nm-emission is readily ascribed to a triplet cluster-centered excited state, ^3^CC*. The microsecond time scale for the emission lifetimes (vide infra) confirms the triplet nature of this excited state. Upon cooling the solid sample at 77 K, this intense band becomes weaker, a new band appears as a shoulder, and this high-energy band then becomes the dominant feature (Figure 9, right). This behavior is clearly reminiscent of certain Cu_4_I_4_P_4_ cubane-containing materials and permits us to assign this higher energy signal to ^3^MXLCT* [15]. Concurrently, the product issued from the reaction **4(1:1)** is pure (Table 4), where **4(1:1)** has been identified as the same product reported in the literature based on the PXRD pattern [9,14]. In this case, the central motif is a rhomboid motif, as shown in Figure 1 (bottom), and is non-emissive at room temperature. Conversely, an emission band was detected at 413 nm at 77 K (Table 5). These behaviors (blue-shifted and not strongly emissive at room temperature) are typical for this motif [1,2]. Moreover, this same behavior has been found for the reliably identified rhomboid motif of the **6(1:1)** reaction mixture.

The rule of thumb dividing the globular vs. quasi-planar families at ~500 nm is not bullet-proof as several cases do not conform to it. For instance, pure **1(2:1)** (step staircase-Cu_4_I_4_(**L1**)_2_, a quasi-planar motif, fused dirhomboid), strongly emits at 597 nm (293 K) and 622 nm (77 K). It is noteworthy that other step staircase-containing materials have also been reported to be strongly emissive [1,2]. Moreover, a temperature dependence of the relative intensity between the high- and low-energy bands, as illustrated for the product in **2(2:1)**, diamond Cu_3_I_3_(**L2**)_2_, is also possible. This is indeed the case for [Cu_3_I_2_(**L1**)_3_]I (**1(1:1)**, [triangular bipyramid]^+^), which is non-emissive at room temperature but is luminescent at 77 K (λ_em_ = 443 nm). One may suspect that the ionic character of this species promotes extra non-radiative deactivation pathways (cation···anion interactions). Table 5 summarizes the emission properties, emission maxima, λ_em_, and quantum yields, QY, for all reaction mixtures (see Figures S41–S106 for all spectra). Room and cryo-temperature spectra for all compounds are provided in Figures S41–S106, except for those which are non-emissive at 293 K or 77 K.

### 2.4. Time-Resolved UV-Vis Spectroscopy and Thermally Activated Delayed Fluorescence (TADF)

The emission lifetimes of the pure compounds (including these with γ-CuI) are placed in Table 6 (all decays are placed in Figures S107–S172). Rigorous analyses of the whole decay traces (χ^2^ ~ 1) indicate that the best fit is mostly a polyphasic decay in the microsecond time scale. This time scale is typical for triplet emissions, and for this type of complexes, and polyexponential decays are not uncommon for solid state samples. The emission decay of **2(3:2)**, an unidentified product (Table 3), at room temperature exhibits short components in the picosecond and nanosecond time scales. These components are most likely associated with a singlet emission strongly overlapping with the triplet luminescence band, thus suggesting a small energy gap between the singlet and triplet excited states. Indeed, upon cooling the sample at 77 K, these short-lived components (ps and short ns) disappear, which is reminiscent of TADF, a phenomenon that is characterized by a strong dependence of relative intensities of fluorescence and phosphorescence, along with the fluorescence lifetime.

**Figure 10:**
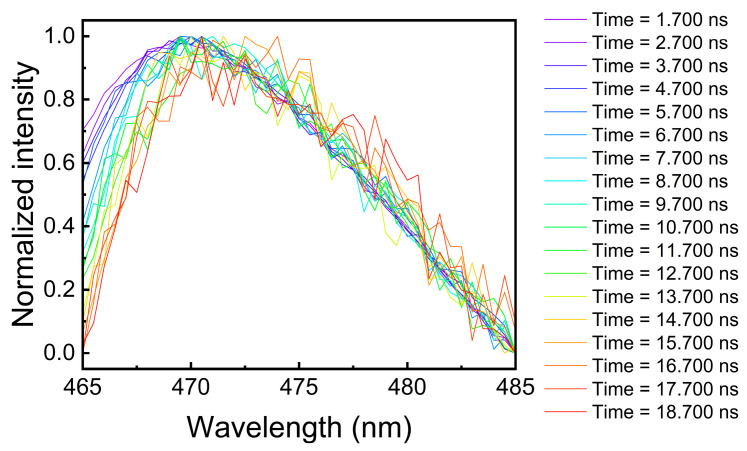
Normalized time-resolved emission spectra of (Cu_2_I_2_(**L5**)_2_) at 293 K issued from the reaction **5(1:1)**. The absolute spectra are available in Figure S173.

**Figure 11:**
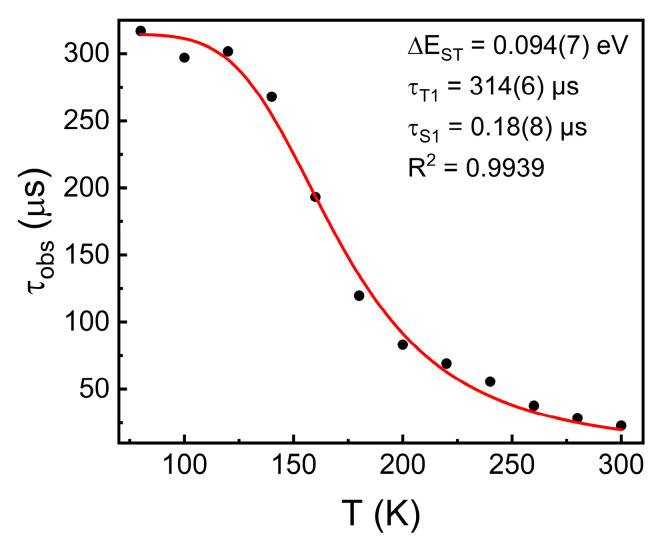
Temperature-dependent lifetime measurements of **5(1:1)**. From these is extracted the energy difference between the S_1_ and T_1_ excited states.

As an example, the reaction mixture **5(1:1)** is known to produce the bridged rhomboid (Cu_2_I_2_(**L5**)_2_) [9,13]. However, ns-components are depicted in the decay trace (Figure S142). The time-resolved emission spectra, TRES, exhibit a very fast decay of the emission envelope at 470 nm, finishing at 473 nm when reaching 18 ns (Figure 10), and longer (i.e., microsecond time scale). At 77 K, these short-lived components disappear (Figure S143). This observation is also reminiscent of TADF. Emission spectra measured at different temperatures also show a shift in the placement of the emission band (whereby cooling down the solid compound produces a slight red-shifting of the emission, Figure S174). Concurrently, measuring the fluorescence and phosphorescence lifetimes of **5(1:1)** at varying temperatures (Figure 11) yields the calculation of an energy difference of 0.094 eV (758 cm^−1^) between the S_1_ and T_1_ excited states (Equation (1)), which confirms the occurrence of TADF. Indeed, a ΔE_ST_ value under 0.38 eV is generally considered to be the condition for the occurrence of TADF.

(1) $$\tau_{obs}=\frac{1+\frac{1}{3}\exp\left(-\frac{\Delta E_{ST}}{k_{B}T}\right)}{\frac{1}{\tau_{S1}}+\frac{1}{3\tau_{T1}}\exp\left(-\frac{\Delta E_{ST}}{k_{B}T}\right)}$$

## 3. Discussion

### 3.1. Statistical Structural Predictability

Three major conclusions can be drawn from the structural data presented herein.

Firstly, the crystallizable materials using bisphosphine ligands lead overwhelmingly to 0D complexes rather than coordination polymers. This is assigned to the quite strong Cu-P bond (especially compared to other reported CPs using CuX, such as with N- or S- ligands) [1,2], as well as to the occasionally poor solubility of the products. However, the presence of CPs, or more precisely poly-dispersed oligomers, within the mixtures, is highly suspected, since a 1D-CP in the series, **3(2:1)**, was previously reported [12], and recent investigations were carried out where [Cu_4_I_4_(PPh_2_R)_2_]_n_-containing CPs (bidentate ligands with R = various rigid chains) were prepared but no crystal suitable for the SCXRD was presented [16,17]. Moreover, large amorphous halos in the presented PXRD patterns can readily be observed in this work.

Secondly, there seems to be a marked stoichiometric preference in the formation of specific SBUs. Namely, cubane and other globally Cu_2_I_2_Lm aggregates strongly favor ligand-poor mixtures. Diamond motifs prefer the slightly less ligand-poor 3:2 ratio, which corresponds to the stoichiometry of the complex. Rhomboid motifs are frequently encountered when the 1:1 ratio is used, but do not occur exclusively within this ratio, as this very stable motif may be preferentially formed in other more ligand-poor mixtures, leaving extra metal salt behind (as seen from residual γ-CuI in the PXRD, Table 4). On the other side of the stoichiometric spectrum, highly ligand-rich mixtures lead almost invariably to CuI centers, which is consistent with the unfavorable energetic costs of leaving the starting uncoordinated ligands.

Thirdly, ligands exhibiting a short bite distance (i.e., **L1** and **L2**) show a preference for globular motifs (diamond, [triangular bipyramid]^+^), probably due to the high bond strain that would be implied by the formation of a rhomboid motif.

### 3.2. Photophysical Trends

Although not bullet-proof, a general trend in the emission (phosphorescence) and photoluminescence lifetimes of CuI-diphosphine complexes can be formed. Indeed, the high- and low-energy bands observed in the emission spectra (under and above 500 nm each) have been previously assigned to ^3^MXLCT*, metal/halide-to-ligand charge transfer, and ^3^CC*, cluster-centered triplet excited states, respectively, for cubane species [18,19]. The ^3^CC* emission becomes more dominant (intense and red-shifted) as the cuprophilic interactions get stronger (i.e., shorter Cu···Cu distances). For some cases, the relative intensities of the ^3^CC* and ^3^MXLCT* bands are temperature-dependent [20]. This simplified version, ^3^CC* (low-energy) and ^3^MXLCT* (high-energy), have recently been updated by complementing this diagram with other upper excited states, which may appear at a lower energy depending on the treatment of the Cu_4_I_4_P_4_ cubane-containing solid [15]. Concurrently, the rhomboid motifs exhibit ^3^MXLCT*-type excited states only [21]. In brief, the emission maximum for the ^3^CC* and ^3^MXLCT*, which in a way may be associated with globular and quasi-planar species, respectively, are mainly placed above and below 500 nm, respectively, at room temperature. Therefore, on the one hand, the occurrence of a high-energy band associated with ^3^MXLCT phosphorescence under 500 nm is assigned to rhomboid and associated quasi-planar motifs. On the other hand, the occurrence of a lower-energy band associated with ^3^CC phosphorescence over 500 nm is assigned to cubane and associated globular motifs. The temperature dependence of the prevalence of ^3^CC over ^3^MXLCT luminescence is also a trademark of globular motifs.

However, this rule has been shown to be of limited certitude, as several cases (e.g., **1(2:1)** and **1(1:1)**) do not conform to it. In these cases, a complete SCXRD study is needed to elucidate the identity of the SBU. However, the deviation from the rule can be explained consequentially.

This tool based on emission wavelength can be complemented by the consideration of the excited state lifetimes. Indeed, short (i.e., ps-ns) lifetimes overlapping with longer (µs-ms) lifetimes can be a sign of a singlet emission strongly overlapping with a triplet emission. This suggests a small singlet-to-triplet energy gap, which would suggest that the lowest energy excited state is ^3^MXLCT*, thus leading to a rhomboid or *quasi-planar* motif. Since the rare signature of TADF implies a very small difference between the S_1_ and T_1_ states, its presence could also be employed to establish a link between the structure and photophysical behavior. Indeed, several rhomboid-containing complexes (Cu_2_I_2_P_4_) have been reported to exhibit TADF [22,23,24]. This is true for other soft ligand complexes involving the rhomboid motif Cu_2_I_2_L_4_ (L = N-, As-, or P- ligand) [24,25,26,27]. Conversely, to the best of our knowledge for Cu_4_I_4_L_4_-cubane-containing materials (L = N-, P-, S-ligand), the literature exhibits no evidence for TADF. This outcome is simply due to the large energy gap between the singlet and triplet excited states for this type of complexes, as evidenced by the very large Stoke shift generally encountered for this motif.

Therefore, quasi-planar motifs can be predicted if there is a prevalence of the following signs: (1) emission under 500 nm, (2) low emission quantum yield, (3) co-occurrence of ps-ns lifetimes with µs-ms lifetimes, (4) temperature-dependence of the lifetimes, where the ps-ns components disappear at low temperatures, and (5) occurrence of TADF.

On the other hand, the prevalence of the following indicates a globular motif: (1) one emission band over 500 nm at 293 K, (2) upon cooling, the appearance of a high energy band (under 500 nm), (3) high emission quantum yield, and (4) no short (ps-ns) lifetimes.

## 4. Materials and Methods

### 4.1. Synthesis

#### 4.1.1. Materials

Copper(I) iodide, and seven bis(diphenylphosphino)alkanes (**L1**, **L2**, **L3**, **L4**, **L5**, **L6**, and **L8**), 1,7-dibromoheptane, and diphenylphosphine (10% wt. in hexanes) were acquired from Millipore Sigma (Burlington, MA, USA), Oakwood Chemicals (Estill, SC, USA), and Ambeed (Arlington Heights, IL, USA). They were used without further purifications.

#### 4.1.2. Procedure for the Synthesis of 1,7-Bis(Diphenylphosphino)Heptane (**L7**)

Powdered KOH (0.6 g, 10.5 mmol) was placed in a three-neck flask (150 mL), equipped with a magnetic stirrer, under Ar atmosphere. DMSO (45 mL) was added, the mixture was stirred at room temperature for 10 min, and diphenylphosphine (10% (wt. in *n*-hexane) solution (10.5 mL, 3.8 mmol) was added dropwise. The resulting pink solution was stirred at room temperature for another 10 min. Subsequently, 1,7-dibromoheptane (0.3 mL, 1.7 mmol) was added dropwise. The reaction mixture was stirred for 5 min after it became colorless and poured into 200 mL of water, followed by bubbling with air for approximately 2 min. The product was extracted with hexanes (3 × 75 mL), organic layers were combined, washed with water and brine, dried over Na_2_SO_4_, filtered, and evaporated to obtain 0.9 g of a crude extract. The reaction was repeated on the same scale and crudes from the two batches (0.9 g and 1.5 g) were combined and chromatophied with silica with the help of a flash machine using a gradient elution from hexanes 100% to hexanes/dichloromethane = 70/30%. The material obtained after chromatography was precipitated twice from hot isopropanol under Ar to obtain 723 mg of the product as a colorless oil. Y = 22%. ^1^H NMR (400 MHz, CDCl_3_, Figure S175): δ 7.47–7.41 (m, 8H), 7.38–7.32 (m, 12H), 2.06 (dd, *J*_1_ = 9.0 Hz, *J*_2_ = 6.1 Hz, 4H), 1.49–1.35 (m, 8H), 1.33–1.24 (m, 2H) ppm. ^31^P NMR (162 MHz, CDCl_3_, Figure S176): −15.71 ppm. ^13^C NMR (100 MHz, CDCl_3_, Figure S177): 132.82, 132.64, 128.63, 128.46, 128.39, 31.07, 30.94, 28.82, 27.76, 25.89, 25.74 ppm.

#### 4.1.3. General Procedure for the Synthesis of Complexes

Following Scheme 1, complexes **1(2:1)** through **8(1:2)** were synthesized in the same manner. As an example, the synthesis of **1(2:1)** is given. Dichloromethane (25 mL) is degassed using argon for 5 min under magnetic stirring at room temperature. Copper(I) iodide (500 mg, 2.62 mmol) is suspended in the solvent, and bis(diphenylphosphino)methane (523 mg, 1.31 mmol) is added. The reaction is stirred for 1–12 h at room temperature. For some compounds, the reaction is completed when a white precipitate appears. For others, it is found to be complete when a clear solution is reached. Precipitates are separated using vacuum filtration. Solubilized products are isolated by the evaporation under reduced pressure of the solvent. Residual solvent traces are removed by the application of a vacuum.

#### 4.1.4. Crystalization

Single crystals of **2(2:1)** were obtained by the slow cooling of a hot solution of the product in propionitrile. CAUTION: Propionitrile is highly acutely toxic (LD_50_ = 39 mg/kg bw oral, rats) through inhalation, dermal, and oral pathways [28]. Single crystals of **8(2:1)** and **1(1:1)** were obtained by the slow evaporation of solutions of the products in chloroform. Single crystals of **6(1:1)** were obtained by layering a portion of hexanes over a concentrated DCM solution of the compound and waiting for the non-solvent to diffuse and cause the apparition of suitably large crystals.

Propionitrile acts as a modulating agent for crystallization, as it can momentarily replace the ligand molecules in the complex. Chloroform and DCM can solubilize the complexes rather easily in some cases. Hexanes is a very clear non-solvent and causes desolubilization.

### 4.2. Instrumentation

#### 4.2.1. Powder X-ray Diffraction Measurements

Powder samples of the reaction mixtures were used for PXRD measurements. A small sample of powder was dispersed over a ZERO diffraction plate from Charles Supper Company and placed for analysis in a BRUKER D8 ADVANCE diffractometer.

Diffractograms were acquired using the DIFFRAC.COMMANDER (version 8.6.3.0) software (accessed on 30 September 2023) from BRUKER using Cu Kα (1.54060 Å) as an X-ray source (40.0 kV, 40.0 mA). Data were collected from 4 to 60° (2θ) over 2046 steps (0.027°step^−1^; 0.50 s.step^−1^) using a LynxEye detector. No further data treatment or baselining was done to the acquired diffractograms.

#### 4.2.2. Single Crystal X-ray Stucture Analyses

Single crystals of **2(2:1)**, **8(2:1)**, **1(1:1)**, and **6(1:1)** were mounted on a Bruker D8 Venture four-circle diffractometer equipped with an Oxford Cryosystems nitrogen jet stream low-temperature system. X-ray radiation was generated with Mo-Kα radiation (λ = 0.71073 Å) or Cu-Kα for **2(2:1),** monochromated through graphite from a microfocus IµS tube from Incoatec GmbH, Geesthacht, Germany. Applying a least-squares fit to the optimized setting angles of the entire set of collected reflections yielded the lattice parameters. Intensity data were recorded as ϕ and ω scans with κ offsets. No significant intensity decay or temperature drift was observed during data collections. SAINT v8.37A (Bruker AXS Inc., Madison, WI, USA, 2015) software was used to reduce data, and absorption correction was effected using SADABS-2016/2 (Bruker, 2016). Structure elucidation was performed using SHELXT (Sheldrick, 2015) with intrinsic phasing. H-atoms were placed geometrically and refined on a riding model. Hydrogen atoms were located from difference Fourier maps, refined at idealized positions riding on the carbon atoms with isotropic displacement parameters U_iso_(H) = 1.2U_eq_(C) or 1.5U_eq_(-CH_3_) and C-H 0.95–1.00 Å. All CH_3_ hydrogen atoms were allowed to rotate but not to tip. Full-matrix least-squares on F^2^ was carried out using SHELXL program (Sheldrick, 2015) on the complete set of reflections. All non-hydrogen atoms were refined with anisotropic displacement parameters, whereas H-atoms were treated in a riding mode [29,30,31]. It is noted that structures **8(2:1)** and **6(1:1)** lie on special positions (Wyckoff sites 8f and 2i, respectively).

#### 4.2.3. Solid-State UV-Vis Absorption Spectra

Solid-state UV-Vis absorption spectra were obtained from the sample dispersed between two quartz plates on a Varian Cary 300 Bio UV-Vis spectrophotometer (sourced from Agilent Technologies Inc., Santa Clara, CA, USA) at 293 K.

#### 4.2.4. Solid-State UV-Vis Photoluminescence Spectra

Solid-state UV-Vis emission and excitation spectra were obtained at 293 K from the sample enclosed in a borosilicate glass capillary on a FLS980 (Edinburgh Instruments) equipped with single monochromators sourced from Edinburgh Instruments Ltd., Kirkton, Scotland, UK. Spectra at 77 K were acquired in the same manner, but the capillaries were immersed in a liquid nitrogen-filled dewar equipped with a transparent tip. All spectra were corrected for instrument response.

#### 4.2.5. Solid-State UV-Vis Photoluminescence Lifetime Measurements

Solid-state UV-Vis photoluminescence lifetimes were measured at 293 K and 77 K on a FLS980 (Edinburgh Instruments) equipped with either a µs-“flash” pulsed lamp or a nano-LED laser (λ_ex_ = 378 nm; 15 mW; full width at half-maximum (FWHM) = 120 ps). Measurements were treated using the time-correlated single-photon counting (TCSPC) method, and data were treated by multiexponential deconvolution analysis. All values have an uncertainty of ±10% based on multiple measurements.

#### 4.2.6. Quantum Yield Measurements

Solid-state emission quantum yields were recorded using a Quanta-φ F-3029 integration sphere from Horiba plugged into a Horiba Fluorolog III, sourced from Horiba Ltd., Kyoto, Japan.

#### 4.2.7. Temperature-Dependent Photoluminescence Measurements

Steady-state emission spectra and luminescence quantum yield measurements were recorded on a Horiba Jobin-Yvon (HJY) Fluorolog-3 (FL3-2iHR550) fluorescence spectrofluorometer equipped with an IR R928P PMT/HJY FL-1073 detector and with an integrating sphere sourced from HORIBA Europe Research Center, Palaiseau 91120, France. Low temperature measurements were allowed by using an OptistatCF (Oxford Inst.) in the range of 77 K to 300 K sourced from Oxford Instruments, Abingdon OX13 5QX, United Kingdom. Excited-state lifetimes in the range of 80 K to 300 K were measured with a delta hub (TCSPC: Time-Correlated Single-Photon Counting) + delta diode system, allowing us to measure excited-state lifetimes between 500 ps and 10 µsand, and with a pulsed xenon source (FL-1035), allowing us to measure excited-state lifetimes longer than 10 µs. Solid samples were placed in a quartz sample holder inside the cryostat and maintained at the desired temperature until equilibrium was reached before recording the spectrum. The experimental data were then fitted according to equation 1 where τ_obs_, τ_S1_, τ_T1_, k_B_, T, and ΔE_ST_ represent the observed lifetime, singlet state decay lifetime, triplet state decay lifetime, Boltzmann constant, temperature, and singlet-triplet energy difference, respectively [32].

## 5. Conclusions

This systematic investigation has tackled the very challenging topic of anticipating the nature of the coordination products between a soft metal halide, in this case CuI, and a soft ditopic ligand, in this case diphosphine of the type Ph_2_P(CH_2_)_m_PPh_2_ (1 ≤ m ≤ 8). We have found a strong stoichiometric dependence, where ligand-poor mixtures form globular complexes preferentially, and vice-versa. We have also found that short-bite ligands form globular SBUs preferentially. Moreover, there is a marked preference for all reaction mixtures used towards 0D complexes. Finally, a prediction tool using emission and excited state lifetimes as markers has been developed for the prediction of globular vs. *quasi*-planar motifs in unidentified CuI-diphosphine complexes.

## Figures and Tables

**Table 1:** Crystal data, data collection, and structure refinement for **2(2:1)**, **8(2:1)**, **1(1:1)**, and **6(1:1)**.

Compound	2(2:1) ∙ 2 PrCN	8(2:1)	1(1:1) ∙ 4CHCl_3_	6(1:1)
Formula	C_60_H_62_Cu_3_I_3_N_2_P_4_	C_64_H_72_Cu_4_I_4_P_4_	C_79_H_70_Cl_12_Cu_3_I_3_P_6_	C_60_H_64_Cu_2_I_2_P_4_
Formula weight/g·mol^−1^	1506.35	1726.85	2201.89	1289.87
Temperature/K	173(2)	173(2)	173(2)	173(2)
Wavelength/Å	1.54178	0.71073	0.71073	0.71073
Crystal system	monoclinic	monoclinic	monoclinic	triclinic
Space group	P2_1_/c	C2/c	P2_1_/c	P -1
a/Å	17.9896(5)	21.253(6)	23.405(8)	9.9268(13)
b/Å	12.3774(4)	15.418(4)	18.989(7)	12.1348(16)
c/Å	26.7592(8)	21.004(5)	19.594(7)	12.5080(16)
α/°	90	90	90	103.0640(10)
β/°	96.468(1)	110.805(2)	93.927(5)	92.427(2)
γ/°	90	90	90	101.5630(10)
Volume/Å^3^	5910.5(3)	6434.0(3)	8688.0(5)	1431.8(3)
Z	4	4	4	1
Density (calc.)/g·cm^−3^	1.693	1.783	1.683	1.496
Absorption coefficient/mm^−1^	14.876	3.361	2.315	1.969
F(000)	2968	3376	4336	648
θ range for data collection/°	2.48 to 71.08	1.67 to 26.41	1.50 to 26.49	1.68 to 26.44
Index ranges	−19 ≤ h ≤ 21 −15 ≤ k ≤ 15 −31 ≤ l ≤ 32	−26 ≤ h ≤ 26 −19 ≤ k ≤ 19 −26 ≤ l ≤ 26	−29 ≤ h ≤ 29 −23 ≤ k ≤ 23 −24 ≤ l ≤ 24	−12 ≤ h ≤ 12 −15 ≤ k ≤ 15 −15 ≤ l ≤ 15
Reflections collected	75,549	54,820	68,189	19,954
Independent reflections	11,215 [R(int) = 0.07]	6588 [R(int) = 0.08]	17,432 [R(int) = 0.15]	5863 [R(int) = 0.02]
Refinement method	Full-matrix least-squares on F^2^	Full-matrix least-squares on F^2^	Full-matrix least-squares on F^2^	Full-matrix least-squares on F^2^
Data/restraints/ parameters	11,215/0/651	6588/0/343	17,432/40/812	5863/0/307
Goodness-of-fit on F^2^	1.073	1.023	0.999	1.040
Final R indices [I > 2σ(I)]	R_1_ = 0.0339 wR_2_ = 0.0819	R_1_ = 0.0344 wR_2_ = 0.0581	R_1_ = 0.0719 wR_2_ = 0.1416	R_1_ = 0.0275 wR_2_ = 0.0598
R indices (all data)	R_1_ = 0.0396 wR_2_ = 0.0849	R_1_ = 0.0619 wR_2_ = 0.0650	R_1_ = 0.1890 wR_2_ = 0.1830	R_1_ = 0.0355 wR_2_ = 0.0628
Largest diff. peak and hole/e·Å^−3^	0.939 and −0.766	0.712 and −0.593	1.562 and −1.378	1.106 and −0.754

**Table 2:** Distances between copper center in newly reported crystal structures showing cuprophilic interactions. Roman numerals correspond to each copper center, as shown in Figure 5, Figure 6, Figure 7 and Figure 8.

Sample	Cu · Cu distances (Å)
**2(2:1)**	2.5216(8) (Cu2-Cu3) 3.1667(8) (Cu1-Cu2) 3.1837(8) (Cu1-Cu3)
**8(2:1**)	2.767(1) (Cu1B-Cu2A) 2.834(1) (Cu2A-Cu2B) 3.0868(9) (Cu1A-Cu2A) 3.202(2) (Cu1A-Cu1B)
**1(1:1)**	3.074(2) (Cu1-Cu3) 3.098(2) (Cu2-Cu3) 3.148(2) (Cu1-Cu2)
**6(1:1)**	3.9822(8) (Cu1A-Cu1B)

**Table 3:** Motif of the CuI aggregates for stoichiometric ratios of CuI:diphosphine ligand ^a–c^.

m	2:1	3:2	1:1	2:3	1:2
1	step staircase [3,4]	diamond [5]	[triangular bipyramid]^+ a^	─	─
2	diamond ^a^	─	chelate-rhomboid [6]	CuI [6,7]	CuI ^b^
3	eared cubane [8] 1D-CP cubane [12]	─	chelate-rhomboid [10,13]	─	─
4	rhomboid ^b^	rhomboid ^b^	bridging-rhomboid [9,14]	CuI ^b^	CuI [11]
5	─	rhomboid ^b,c^	bridging-rhomboid [9,13]	─	─
6	─	rhomboid ^b,c^	bridging-rhomboid ^a^	─	─
7	─	─	─	─	─
8	cubane ^a,c^	─	─	─	─

^a^ The identification was performed using SCXRD. ^b^ The identification was made by comparing the PXRD pattern with a literature pattern, which was reliably identified. ^c^ These samples are reliably identified. However, samples **5(3:2)**, **6(3:2)**, and **8(2:1)** contain another unidentified species.

**Table 4:** Nature of the reaction mixtures of the CuI/diphosphine reactions in CH_2_Cl_2_ after washing and isolation.

m	2:1	3:2	1:1	2:3	1:2
1	pure	mixture ^a^ + γ-CuI	mixture ^a^	unknown but pure	mixture ^a^
2	mixture ^a^ + γ-CuI	pure + γ-CuI	mixture ^a^	pure	pure
3	mixture ^a^	mixture ^a^	mixture ^a^	mixture ^a^	mixture ^a^
4	pure + γ-CuI	pure + γ-CuI	pure	pure	pure
5	mixture + γ-CuI	mixture γ-CuI	mixture	mixture ^a^	mixture ^a^
6	mixture ^a^ + γ-CuI	mixture ^a^	pure	mixture ^a^	mixture ^a^
7	unknown but pure + γ-CuI	mixture ^a^	mixture ^a^	mixture ^a^	mixture ^a^
8	mixture ^a^	mixture ^a^	mixture ^a^	mixture ^a^	mixture ^a^

^a^ Presence of an amorphous phase has been noted in the mixture.

**Table 5:** Steady-state photophysical parameters for the reaction mixtures at 293 K λ_em_ (QY) and 77 K λ_em_ (in italics).

Ligand	2:1	3:2	1:1	2:3	1:2
**L1**	597 nm (0.19) *622 nm*	720 nm (<0.01) *423, 667 nm*	─ ^a^ *443 nm*	644 nm (─ ^b^) *441, 679 nm*	─ ^a^ *441 nm*
**L2**	555 nm (0.03) *435, 471 nm*	542 nm (<0.01) *405, 500 nm*	546 nm (<0.01) *445, 761 nm*	─ ^a^ *430 nm*	─ ^a^ *443 nm*
**L3**	560 nm (0.08) *430, 600 nm*	590 nm (0.06) *450, 600 nm*	483 nm (0.04) *430 nm*	476 nm (0.01) *445 nm*	─ ^a^ *459 nm*
**L4**	510 nm (0.02) *470 nm*	576 nm (0.03) *439, 475 nm*	─ ^a^ *413 nm*	488 nm (0.02) *475 nm*	─ ^a^ *417 nm*
**L5**	585 nm (0.31) *433, 599 nm*	586 nm (0.14) *435, 569 nm*	475 nm (0.01) *464 nm*	479 nm (<0.01) *441 nm*	─ ^a^ *434 nm*
**L6**	584 nm (0.39) *428, 586 nm*	588 nm (0.17) *429, 589 nm*	─ ^a^ *431 nm*	488 nm (─ ^b^) *438 nm*	─ ^a^ *433 nm*
**L7**	544 nm (0.45) *457, 563 nm*	585 nm (0.37) *433, 588 nm*	560 nm (0.05) *430 nm*	520 nm (0.01) *474 nm*	513 nm (─ ^b^) *433 nm*
**L8**	566 nm (0.24) *430, 572 nm*	588 nm (0.08) *429, 579 nm*	470 nm (0.01) *435, 463 nm*	483 nm (<0.01) *430 nm*	─ ^a^ *440 nm*

^a^ No emission was observed. ^b^ Measurement below the limit of detection.

**Table 6:** Emission lifetimes of selected compounds. ^a^ Lifetimes for all compounds are listed in the Supplementary Materials, Table S1.

Sample	τ_1_ (f_1_,%)	τ_2_ (f_2_,%)	τ_3_ (f_3_,%)	τ_4_ (f_4_,%)	χ^2^	Notes
**1(2:1)**	1.03 µs (1.64)	6.86 µs (98.4)	n.a.	n.a.	1.032	293 K
	366 ns (28.3)	1.65 µs (48.0)	6.38 µs (23.7)	n.a.	1.156	77 K, short lifetimes
	37.0 µs (100)	n.a.	n.a.	n.a.	1.053	77 K, long lifetimes
**2(3:2)**	<100 ps (21.8)	247 ps (28.5)	1.30 ns (49.7)	n.a.	1.070	293 K, short lifetimes
	139 ns (11.5)	704 ns (27.5)	3.25 µs (61.0)	n.a.	1.076	293 K, long lifetimes
	8.82 µs (72.7)	41.0 µs (14.0)	160 µs (13.3)	n.a.	1.094	77 K
**4(2:1)**	1.73 (49.9)	5.87 µs (31.3)	42.5 µs (18.8)	n.a.	1.011	293 K
	7.85 µs (34.2)	54.2 µs (33.4)	250 µs (32.4)	n.a.	1.024	77 K, short lifetimes
	26.5 µs (1.25)	1.60 ms (13.2)	9.40 ms (85.6)	n.a.	1.043	77 K, long lifetimes
**4(3:2)**	6.54 µs (19.3)	17.9 µs (56.7)	91.3 µs (24.0)	n.a.	1.011	293 K
	308 ps (16.1)	1.23 ns (53.9)	4.85 ns (30.0)	n.a.	1.069	77 K, short lifetimes
	120 µs (5.08)	1.60 ms (8.03)	8.70 ms (86.9)	n.a.	1.045	77 K, long lifetimes
**4(1:1)**	36.9 µs (22.2)	110 µs (47.3)	380 µs (30.6)	n.a.	1.059	77 K
**4(2:3)**	<100 ps (5.94)	612 ps (16.2)	2.24 ns (41.6)	6.15 ns (36.3)	1.075	293 K
	487 ps (20.3)	1.61 ns (39.3)	5.25 ns (40.4)	n.a.	1.040	77 K, short lifetimes
	190 µs (9.86)	1.10 ms (11.4)	10.0 ms (78.7)	n.a.	1.003	77 K, long lifetimes
**4(1:2)**	17.1 µs (19.9)	68.1 µs (50.7)	290 µs (29.5)	n.a.	1.063	77 K
**6(1:1)**	294 ns (20.1)	55.8 µs (49.6)	110 µs (30.3)	n.a.	1.036	77 K
**7(2:1)**	1.30 µs (3.79)	5.00 µs (96.2)	n.a.	n.a.	1.088	293 K

^a^ f_i_(%) = (B_i_τ_i_)/Σ(B_i_τ_i_) ×100%; I_em_(t) = Σ(B_i_ exp(−t/τ_i_); n.a.: no further lifetime component found.

## Data Availability

In agreement with the policies of Canadian funding agencies, the Canadian public may access data used for this research from the authors.

## Supplementary Materials

The following supporting information can be downloaded at: https://www.mdpi.com/article/10.3390/molecules28237781/s1, Figures S1–S177: supplementary data; Table S1: PL decay lifetimes for all compounds. Crystal structures can be accessed from the Cambridge crystallographic database: www.ccdc.cam.ac.uk/structures/ [accessed on 24 November 2023] under accession codes 2304966; 2304967; 2304968; 2304969.
