# Supplementary material for: Statistical Analysis of Copper(I) Iodide and Bis(Diphenylphosphino)alkane-Based Complexes and Coordination Polymers
Source: Molecules. 2023 Nov 25;28(23):7781. doi: 10.3390/molecules28237781 (PMC10708141; doi:10.3390/molecules28237781)
Supplement: Supplementary file 1 [file molecules-28-07781-s001.zip › molecules-2723740-supplementary.pdf]

Supplementary information

Statistical analysis of Copper(I) Iodide and Bis(diphenylphosphino)alkane-Based Complexes and Coordination Polymers

Léo Boivin[1], Adrien Schlachter[2], Daniel Fortin[1], Christophe Lescop[2] and Pierre D. Harvey[1*]

[1]*Département de Chimie, Université de Sherbrooke, Sherbrooke, QC J1K 2R1, Canada*

[2]*ISCR (Institut des Sciences Chimiques de Rennes)—UMR 6226, CNRS, INSA Rennes, Unversity Rennes, F-35000 Rennes, France*

TABLE OF CONTENTS

Figure S1. PXRD pattern of **1(2:1)**, compared to the calculated pattern for the step-staircase motif as reported by references [1,2] 7
Figure S2. PXRD pattern of **2(2:1)**, compared to the calculated pattern for the diamond motif as reported by this work. Large peaks at 25.7°, 42.4°, and 50.1° are due to the presence of excess copper(I) iodide 7
Figure S3. PXRD pattern of **3(2:1)**, compared to the calculated pattern for the eared cubane motif as reported by reference [3]. Large peaks at 25.7°, 42.4°, and 50.1° are due to the presence of excess copper(I) iodide 8
Figure S4. PXRD pattern of **4(2:1)**, compared to the calculated pattern for the rhomboid motif as reported by references [4,5]. Large peaks at 25.7°, 42.4°, and 50.1° are due to the presence of excess copper(I) iodide 8
Figure S5. PXRD pattern of **5(2:1)**, compared to the calculated pattern for the rhomboid motif as reported by references [5,6]. Due to the large halo, no conclusive structural determination can be made. Large peaks at 25.7°, 42.4°, and 50.1° are due to the presence of excess copper(I) iodide 9
Figure S6. PXRD pattern of **6(2:1)**. Large peaks at 25.7°, 42.4°, and 50.1° are due to the presence of excess copper(I) iodide 9
Figure S7. PXRD pattern of **7(2:1)**. Large peaks at 25.7°, 42.4°, and 50.1° are due to the presence of excess copper(I) iodide 10
Figure S8. PXRD pattern of **8(2:1)**, compared to the calculated pattern for the cubane motif as reported by this work 10
Figure S9. PXRD pattern of **1(3:2)**, compared to the calculated pattern for the diamond motif as reported by reference [7]. Large peaks at 25.7°, 42.4°, and 50.1° are due to the presence of excess copper(I) iodide 11
Figure S10. PXRD pattern of **2(3:2)** 11
Figure S11. PXRD pattern of **3(3:2)** 12

Figure S12. PXRD pattern of **4(3:2)**, compared to the calculated pattern for the rhomboid motif as reported by references [4,5]. Large peaks at 25.7°, 42.4°, and 50.1° are due to the presence of excess copper(I) iodide 12
Figure S13. PXRD pattern of **5(3:2)**, compared to the calculated pattern for the rhomboid motif as reported by reference [5,6]. This sample is concluded to be a mix of the rhomboid motif and another unknown motif. Peaks at 25.7°, 42.4°, and 50.1° are due to the presence of excess copper(I) iodide. 13
Figure S14. PXRD pattern of **6(3:2)** compared to the calculated pattern for the rhomboid motif as reported by this work. This sample is concluded to be a mix of the rhomboid motif and another unknown motif........ 13
Figure S15. PXRD pattern of **7(3:2)**........14
Figure S16. PXRD pattern of **8(3:2)**........14
Figure S17. PXRD pattern of **1(1:1)**, compared to the calculated pattern for the bipyramid motif as reported by this work........ 15
Figure S18. PXRD pattern for **2(1:1)**........15
Figure S19. PXRD pattern of **3(1:1)**, compared to the calculated pattern for the rhomboid motif as reported by references [6,8]........ 16
Figure S20. PXRD pattern of **4(1:1)**, compared to the calculated pattern for the rhomboid motif as reported by references [4,5]........ 16
Figure S21. PXRD pattern of **5(1:1)**, compared to the calculated pattern for the rhomboid motif as reported by references [5,6]........ 17
Figure S22. PXRD pattern of **6(1:1)** compared to the calculated pattern for the rhomboid motif as reported by this work 17
Figure S23. PXRD pattern of **7(1:1)**........18
Figure S24. PXRD pattern of **8(1:1)**........18
Figure S25. PXRD pattern of **1(2:3)**........19
Figure S26. PXRD pattern of **2(2:3)**, compared to the calculated pattern for the diatomic linear motif as reported by references [9,10] 19
Figure S27. PXRD pattern of **3(2:3)**, compared to the calculated pattern for the rhomboid motif as reported by references [6,8]........ 20
Figure S28. PXRD pattern of **4(2:3)**........20
Figure S29. PXRD pattern of **5(2:3)**, compared to the calculated pattern for the rhomboid motif as reported by reference [5,6]........ 21
Figure S30. PXRD pattern of **6(2:3)**........21
Figure S31. PXRD pattern of **7(2:3)**........22
Figure S32. PXRD pattern of **8(2:3)**........22
Figure S33. PXRD pattern of **1(1:2)**........23
Figure S34. PXRD pattern of **2(1:2)**, compared to th calculated pattern for the diatomic linear motif as reported by references [9,10] 23
Figure S35. PXRD pattern of **3(1:2)**........24

Figure S36. PXRD pattern of **4(1:2)**..24
Figure S37. PXRD pattern of **5(1:2)**..25
Figure S38. PXRD pattern of **6(1:2)**..25
Figure S39. PXRD pattern of **7(1:2)**..26
Figure S40. PXRD pattern of **8(1:2)**..26
Figure S41. UV-Vis absorption and PL spectra of **1(2:1)**. Left : 293K. Right : 77K.....................27
Figure S42. UV-Vis absorption and PL spectra of **2(2:1)**. Left : 293K. Right : 77K.....................27
Figure S43. UV-Vis absorption and PL spectra of **3(2:1)**. Left : 293K. Right : 77K.....................28
Figure S44. UV-Vis absorption and PL spectra of **4(2:1)**. Left : 293K. Right : 77K.....................28
Figure S45. UV-Vis absorption and PL spectra of **5(2:1)**. Left : 293K. Right : 77K.....................29
Figure S46. UV-Vis absorption and PL spectra of **6(2:1)**. Left : 293K. Right : 77K.....................29
Figure S47. UV-Vis absorption and PL spectra of **7(2:1)**. Left : 293K. Right : 77K.....................30
Figure S48. UV-Vis absorption and PL spectra of **8(2:1)**. Left : 293K. Right : 77K.....................30
Figure S49. UV-Vis absorption and PL spectra of **1(3:2)**. Left : 293K. Right : 77K.....................31
Figure S50. UV-Vis absorption and PL spectra of **2(3:2)**. Left : 293K. Right : 77K.....................31
Figure S51. UV-Vis absorption and PL spectra of **3(3:2)**. Left : 293K. Right : 77K.....................32
Figure S52. UV-Vis absorption and PL spectra of **4(3:2)**. Left : 293K. Right : 77K.....................32
Figure S53. UV-Vis absorption and PL spectra of **5(3:2)**. Left : 293K. Right : 77K.....................33
Figure S54. UV-Vis absorption and PL spectra of **6(3:2)**. Left : 293K. Right : 77K.....................33
Figure S55. UV-Vis absorption and PL spectra of **7(3:2)**. Left : 293K. Right : 77K.....................34
Figure S56. UV-Vis absorption and PL spectra of **8(3:2)**. Left : 293K. Right : 77K.....................34
Figure S57. UV-Vis absorption and PL spectra of **1(1:1)**. Left : 293K. Right : 77K.....................35
Figure S58. UV-Vis absorption and PL spectra of **2(1:1)**. Left : 293K. Right : 77K.....................35
Figure S59. UV-Vis absorption and PL spectra of **3(1:1)**. Left : 293K. Right : 77K.....................36
Figure S60. UV-Vis absorption and PL spectra of **4(1:1)**. Left : 293K. Right : 77K.....................36
Figure S61. UV-Vis absorption and PL spectra of **5(1:1)**. Left : 293K. Right : 77K.....................37
Figure S62. UV-Vis absorption and PL spectra of **6(1:1)**. Left : 293K. Right : 77K.....................37
Figure S63. UV-Vis absorption and PL spectra of **7(1:1)**. Left : 293K. Right : 77K.....................38
Figure S64. UV-Vis absorption and PL spectra of **8(1:1)**. Left : 293K. Right : 77K.....................38
Figure S65. UV-Vis absorption and PL spectra of **1(2:3)**. Left : 293K. Right : 77K.....................39
Figure S66. UV-Vis absorption and PL spectra of **2(2:3)**. Left : 293K. Right : 77K.....................39
Figure S67. UV-Vis absorption and PL spectra of **3(2:3)**. Left : 293K. Right : 77K.....................40
Figure S68. UV-Vis absorption and PL spectra of **4(2:3)**. Left : 293K. Right : 77K.....................40
Figure S69. UV-Vis absorption and PL spectra of **5(2:3)**. Left : 293K. Right : 77K.....................41
Figure S70. UV-Vis absorption and PL spectra of **6(2:3)**. Left : 293K. Right : 77K.....................41
Figure S71. UV-Vis absorption and PL spectra of **7(2:3)**. Left : 293K. Right : 77K.....................42
Figure S72. UV-Vis absorption and PL spectra of **8(2:3)**. Left : 293K. Right : 77K.....................42
Figure S73. UV-Vis absorption and PL spectra of **1(1:2)**. Left : 293K. Right : 77K.....................43
Figure S74. UV-Vis absorption and PL spectra of **2(1:2)**. Left : 293K. Right : 77K.....................43
Figure S75. UV-Vis absorption and PL spectra of **3(1:2)**. Left : 293K. Right : 77K.....................44

Figure S76. UV-Vis absorption and PL spectra of **4(1:2)**. Left : 293K. Right : 77K....................44
Figure S77. UV-Vis absorption and PL spectra of **5(1:2)**. Left : 293K. Right : 77K....................45
Figure S78. UV-Vis absorption and PL spectra of **6(1:2)**. Left : 293K. Right : 77K....................45
Figure S79. UV-Vis absorption and PL spectra of **7(1:2)**. Left : 293K. Right : 77K....................46
Figure S80. UV-Vis absorption and PL spectra of **8(1:2)**. Left : 293K. Right : 77K....................46
Figure S81. Photoluminescence and quantum yield of **1(2:1)** 47
Figure S82. Photoluminescence and quantum yield of **2(2:1)** 47
Figure S83. Photoluminescence and quantum yield of **3(2:1)** 48
Figure S84. Photoluminescence and quantum yield of **4(2:1)** 48
Figure S85. Photoluminescence and quantum yield of **5(2:1)** 49
Figure S86. Photoluminescence and quantum yield of **6(2:1)** 49
Figure S87. Photoluminescence and quantum yield of **7(2:1)** 50
Figure S88. Photoluminescence and quantum yield of **8(2:1)** 50
Figure S89. Photoluminescence and quantum yield of **1(3:2)** 51
Figure S90. Photoluminescence and quantum yield of **2(3:2)** 51
Figure S91. Photoluminescence and quantum yield of **3(3:2)** 52
Figure S92. Photoluminescence and quantum yield of **4(3:2)** 52
Figure S93. Photoluminescence and quantum yield of **5(3:2)** 53
Figure S94. Photoluminescence and quantum yield of **6(3:2)** 53
Figure S95. Photoluminescence and quantum yield of **7(3:2)** 54
Figure S96. Photoluminescence and quantum yield of **8(3:2)** 54
Figure S97. Photoluminescence and quantum yield of **2(1:1)** 55
Figure S98. Photoluminescence and quantum yield of **3(1:1)** 55
Figure S99. Photoluminescence and quantum yield of **5(1:1)** 56
Figure S100. Photoluminescence and quantum yield of **7(1:1)** 56
Figure S101. Photoluminescence and quantum yield of **8(1:1)** 57
Figure S102. Photoluminescence and quantum yield of **3(2:3)** 57
Figure S103. Photoluminescence and quantum yield of **4(2:3)** 58
Figure S104. Photoluminescence and quantum yield of **5(2:3)** 58
Figure S105. Photoluminescence and quantum yield of **7(2:3)** 59
Figure S106. Photoluminescence and quantum yield of **8(2:3)** 59
Figure S107. PL decay of **1(2:1)** at 293K...60
Figure S108. PL decay of **1(2:1)** at 77K. Left : short lifetimes. Right : long lifetimes..................60
Figure S109. PL decay of **2(2:1)** at 293K...61
Figure S110. PL decay of **2(2:1)** at 77K...61
Figure S111. PL decay of **3(2:1)** at 293K...62
Figure S112. PL decay of **3(2:1)** at 77K...62
Figure S113. PL decay of **4(2:1)** at 293K...63
Figure S114. PL decay of **4(2:1)** at 77K. Left : short lifetimes. Right : long lifetimes..................63
Figure S115. PL decay of **5(2:1)** at 293K...64

Figure S116. PL decay of **5(2:1)** at 77K 64
Figure S117. PL decay of **6(2:1)** at 293K 65
Figure S118. PL decay of **7(2:1)** at 293K 65
Figure S119. PL decay of **8(2:1)** at 293K 66
Figure S120. PL decay of **1(3:2)** at 293K. Left : short lifetimes. Right : long lifetimes 66
Figure S121. PL decay of **1(3:2)** at 77K. Left : short lifetimes. Right : long lifetimes 67
Figure S122. PL decay of **2(3:2)** at 293K. Left : short lifetimes. Right : long lifetimes 67
Figure S123. PL decay of **2(3:2)** at 77K 68
Figure S124. PL decay of **3(3:2)** at 293K 68
Figure S125. PL decay of **3(3:2)** at 77K 69
Figure S126. PL decay of **4(3:2)** at 293K 69
Figure S127. PL decay of **4(3:2)** at 77K. Left : short lifetimes. Right : long lifetimes 70
Figure S128. PL decay of **5(3:2)** at 293K 70
Figure S129. PL decay of **5(3:2)** at 77K 71
Figure S130. PL decay of **6(3:2)** at 293K 71
Figure S131. PL decay of **6(3:2)** at 77K 72
Figure S132. PL decay of **7(3:2)** at 293K 72
Figure S133. PL decay of **7(3:2)** at 77K 73
Figure S134. PL decay of **8(3:2)** at 293K 73
Figure S135. PL decay of **8(3:2)** at 77K 74
Figure S136. PL decay of **1(1:1)** at 77K 74
Figure S137. PL decay of **2(1:1)** at 293K 75
Figure S138. PL decay of **2(1:1)** at 77K 75
Figure S139. PL decay of **3(1:1)** at 293K 76
Figure S140. PL decay of **3(1:1)** at 77K 76
Figure S141. PL decay of **4(1:1)** at 77K 77
Figure S142. PL decay of **5(1:1)** at 293K. Left : short lifetimes. Right : long lifetimes 77
Figure S143. PL decay of **5(1:1)** at 77K 78
Figure S144. PL decay of **6(1:1)** at 77K 78
Figure S145. PL decay of **7(1:1)** at 293K. Left : short lifetimes. Right : long lifetimes 79
Figure S146. PL decay of **7(1:1)** at 77K. Left : short lifetimes. Right : long lifetimes 79
Figure S147. PL decay of **8(1:1)** at 77K 80
Figure S148. PL decay of **1(2:3)** at 293K 80
Figure S149. PL decay of **1(2:3)** at 77K 81
Figure S150. PL decay of **2(2:3)** at 293K 81
Figure S151. PL decay of **2(2:3)** at 77K. Left : short lifetimes. Right : long lifetimes 82
Figure S152. PL decay of **3(2:3)** at 293K 82
Figure S153. PL decay of **3(2:3)** at 77K 83
Figure S154. PL decay of **4(2:3)** at 293K 83
Figure S155. PL decay of **4(2:3)** at 77K. Left : short lifetimes. Right : long lifetimes 84

Figure S156. PL decay of **5(2:3)** at 293K. Left : short lifetimes. Right : long lifetimes................84
Figure S157. PL decay of **5(2:3)** at 77K...85
Figure S158. PL decay of **6(2:3)** at 293K. Left : short lifetimes. Right : long lifetimes................85
Figure S159. PL decay of **6(2:3)** at 77K...86
Figure S160. PL decay of **7(2:3)** at 77K. Left : short lifetimes. Right : long lifetimes.................86
Figure S161. PL decay of **7(2:3)** at 77K. Left : short lifetimes. Right : long lifetimes.................87
Figure S162. PL decay of **8(2:3)** at 293K. Left : short lifetimes. Right : long lifetimes................87
Figure S163. PL decay of **8(2:3)** at 77K. Left : short lifetimes. Right : long lifetimes.................88
Figure S164. PL decay of **1(1:2)** at 77K...88
Figure S165. PL decay of **2(1:2)** at 77K...89
Figure S166. PL decay of **3(2:1)** at 77K...89
Figure S167. PL decay of **4(1:2)** at 77K...90
Figure S168. PL decay of **5(1:2)** at 77K...90
Figure S169. PL decay of **6(1:2)** at 77K...91
Figure S170. PL decay of **7(1:2)** at 293K...91
Figure S171. PL decay of **7(1:2)** at 77K...92
Figure S172. PL decay of **8(1:2)** at 77K...92
Table S1. PL decay lifetime components for all compounds...93
Figure S173. Absolute time-resolved emission spectra of **5(1:1)** at 293K. λ_{ex} = 378nm...............97
Figure S174. Normalized emission spectra of **5(1:1)** dependent on the temperature.....................98
Figure S175. 400 MHz ^{1}H NMR spectrum of **L7** in $CDCl_3$...99
Figure S176. 162 MHz ^{31}P NMR spectrum of **L7** in $CDCl_3$...100
Figure S177. 100 MHz ^{13}C NMR spectrum of **L7** in $CDCl_3$...101
References...101

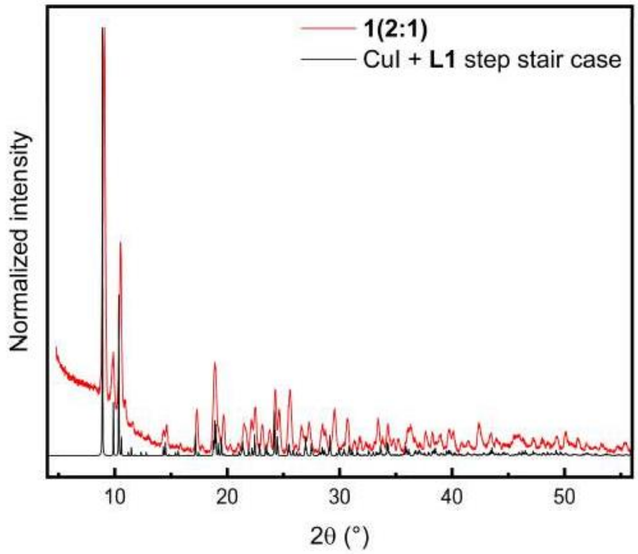

Figure S1. PXRD pattern of **1(2:1)**, compared to the calculated pattern for the step-staircase motif as reported by references [1,2].

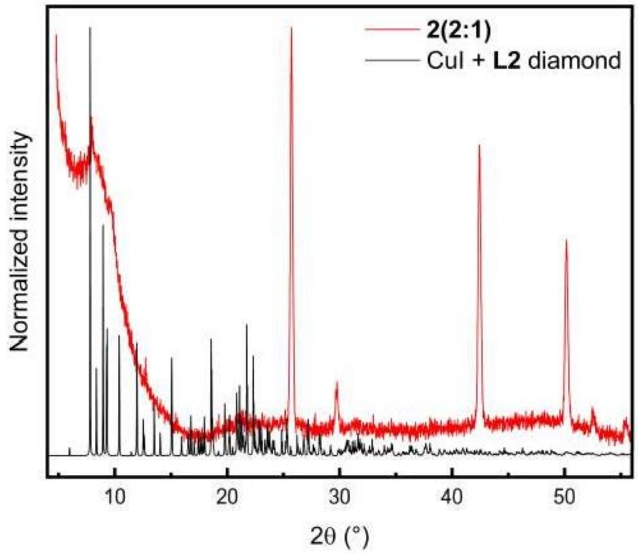

Figure S2. PXRD pattern of **2(2:1)**, compared to the calculated pattern for the diamond motif as reported by this work. Large peaks at 25.7°, 42.4°, and 50.1° are due to the presence of excess copper(I) iodide.

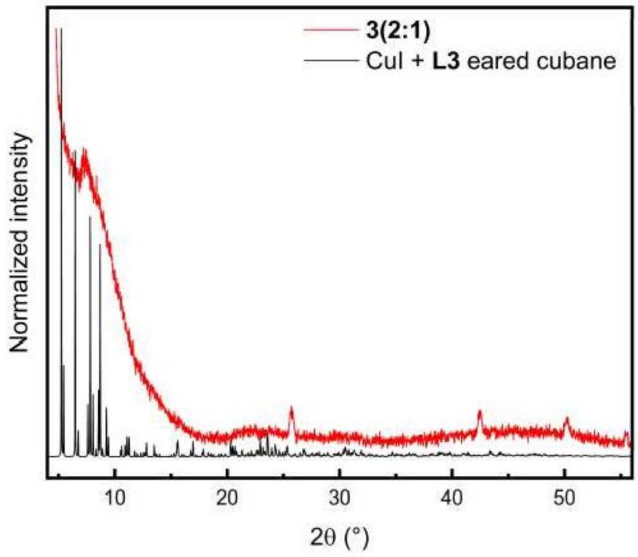

Figure S3. PXRD pattern of **3(2:1)**, compared to the calculated pattern for the eared cubane motif as reported by reference [3]. Large peaks at 25.7°, 42.4°, and 50.1° are due to the presence of excess copper(I) iodide.

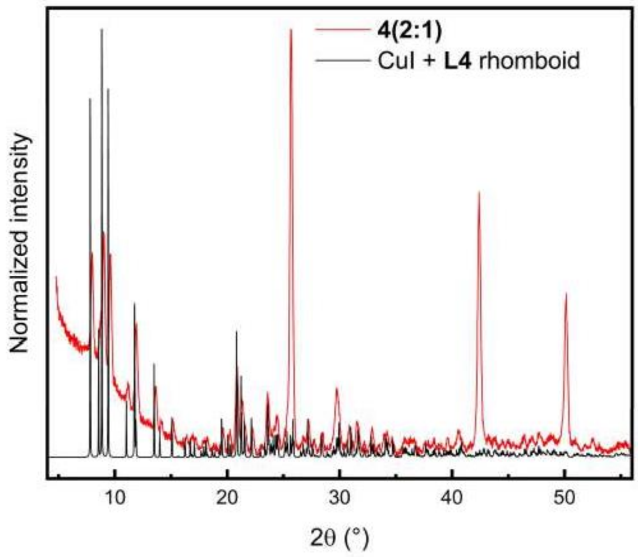

Figure S4. PXRD pattern of **4(2:1)**, compared to the calculated pattern for the rhomboid motif as reported by references [4,5]. Large peaks at 25.7°, 42.4°, and 50.1° are due to the presence of excess copper(I) iodide.

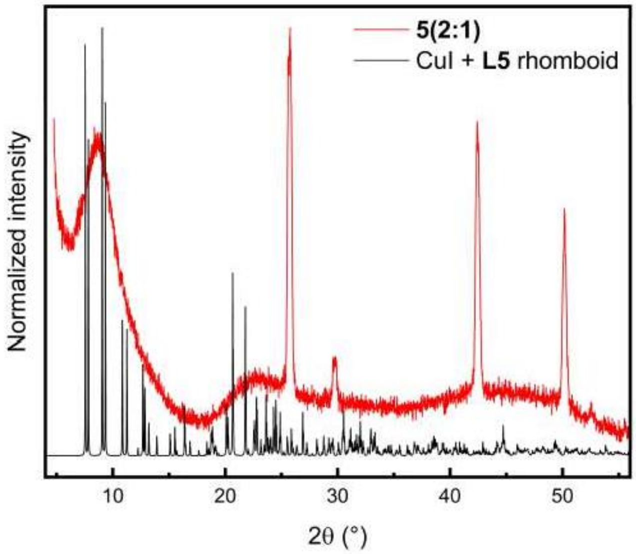

Figure S5. PXRD pattern of **5(2:1)**, compared to the calculated pattern for the rhomboid motif as reported by references [5,6]. Due to the large halo, no conclusive structural determination can be made. Large peaks at 25.7°, 42.4°, and 50.1° are due to the presence of excess copper(I) iodide.

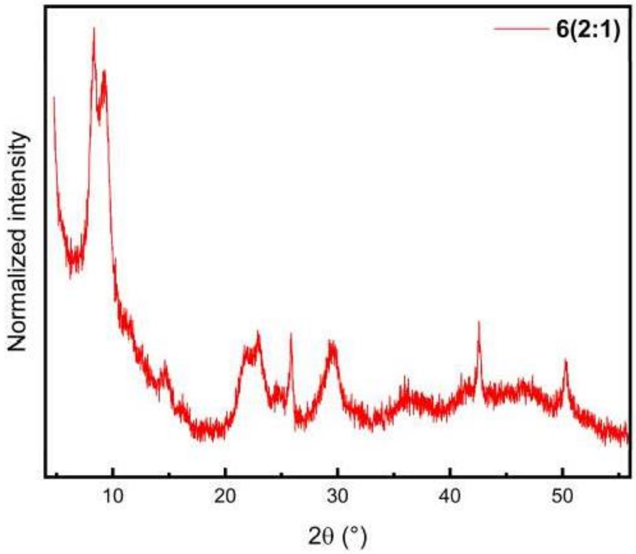

Figure S6. PXRD pattern of **6(2:1)**. Large peaks at 25.7°, 42.4°, and 50.1° are due to the presence of excess copper(I) iodide.

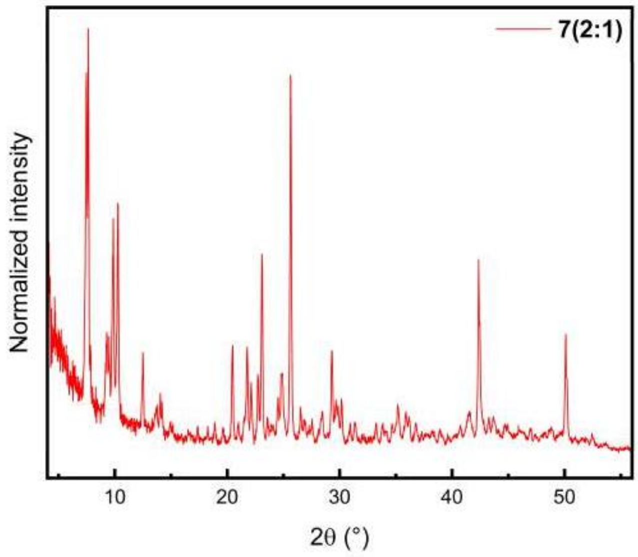

Figure S7. PXRD pattern of **7(2:1)**. Large peaks at 25.7°, 42.4°, and 50.1° are due to the presence of excess copper(I) iodide.

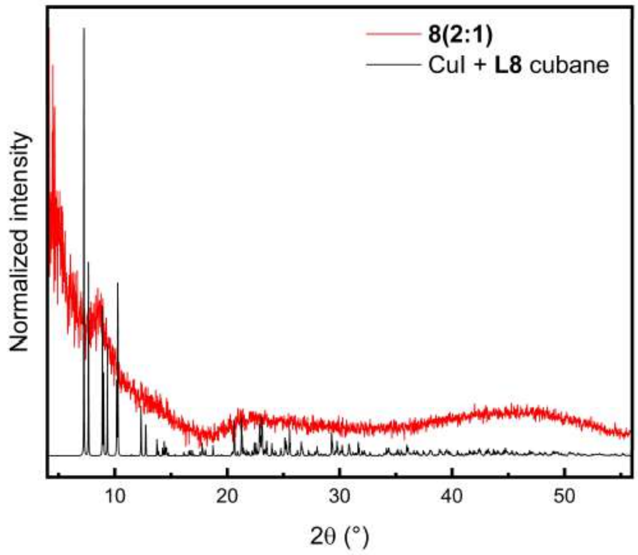

Figure S8. PXRD pattern of **8(2:1)**, compared to the calculated pattern for the cubane motif as reported by this work.

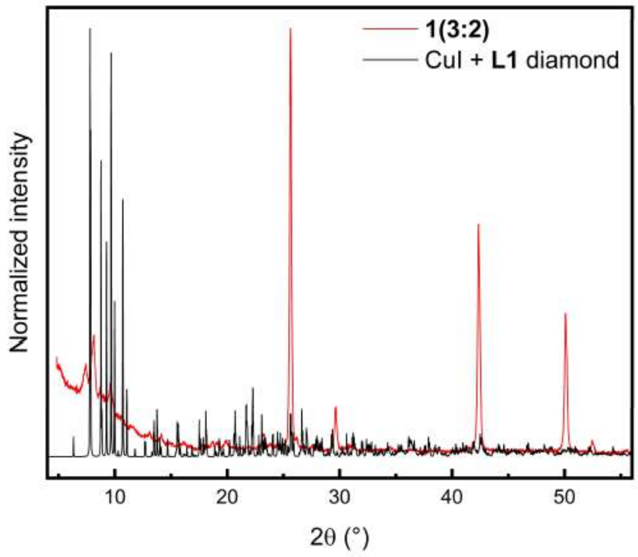

Figure S9. PXRD pattern of **1(3:2)**, compared to the calculated pattern for the diamond motif as reported by reference [7]. Large peaks at 25.7°, 42.4°, and 50.1° are due to the presence of excess copper(I) iodide.

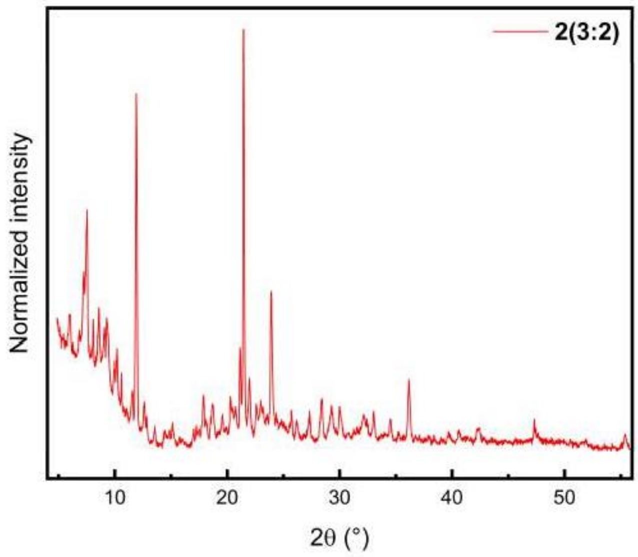

Figure S10. PXRD pattern of **2(3:2)**.

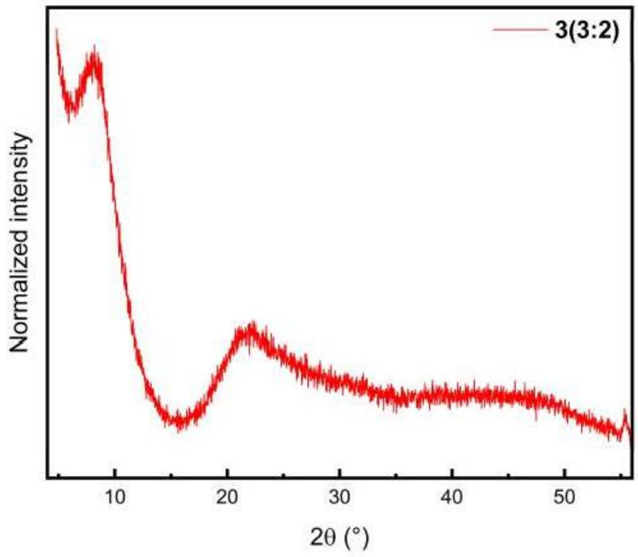

Figure S11. PXRD pattern of **3(3:2)**.

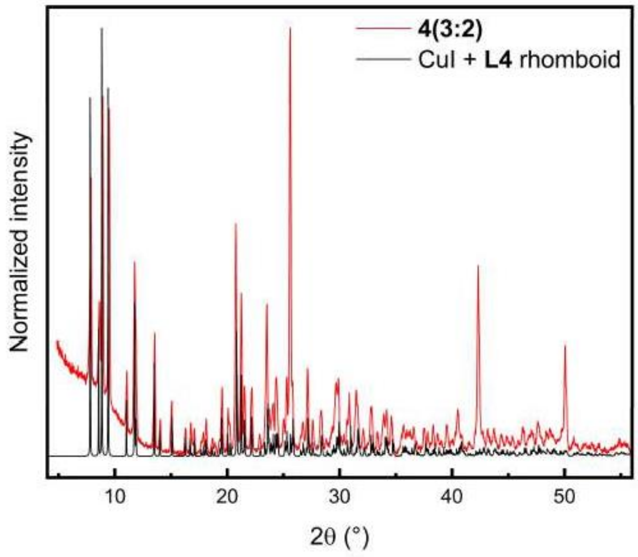

Figure S12. PXRD pattern of **4(3:2)**, compared to the calculated pattern for the rhomboid motif as reported by references [4,5]. Large peaks at 25.7°, 42.4°, and 50.1° are due to the presence of excess copper(I) iodide.

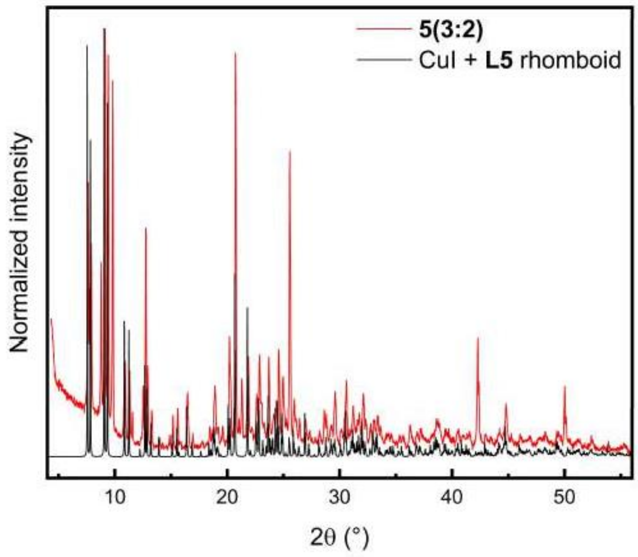

Figure S13. PXRD pattern of **5(3:2)**, compared to the calculated pattern for the rhomboid motif as reported by reference [5,6]. This sample is concluded to be a mix of the rhomboid motif and another unknown motif. Peaks at 25.7°, 42.4°, and 50.1° are due to the presence of excess copper(I) iodide.

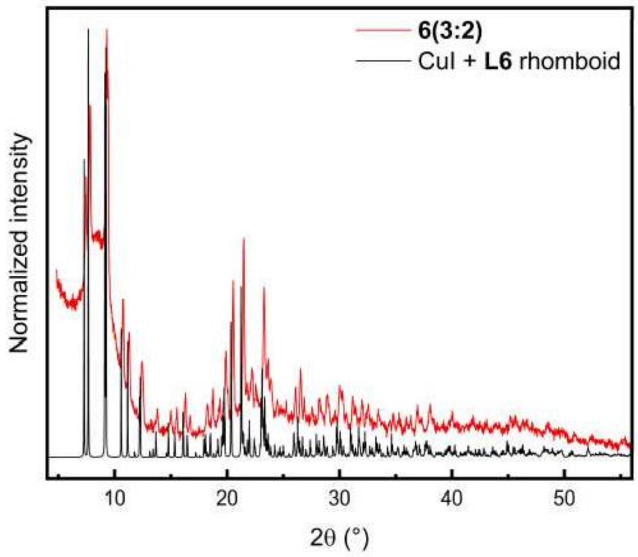

Figure S14. PXRD pattern of **6(3:2)** compared to the calculated pattern for the rhomboid motif as reported by this work. This sample is concluded to be a mix of the rhomboid motif and another unknown motif.

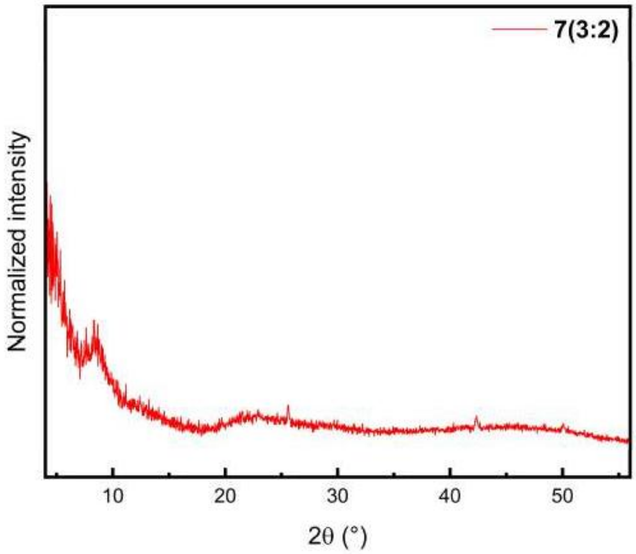

Figure S15. PXRD pattern of **7(3:2)**.

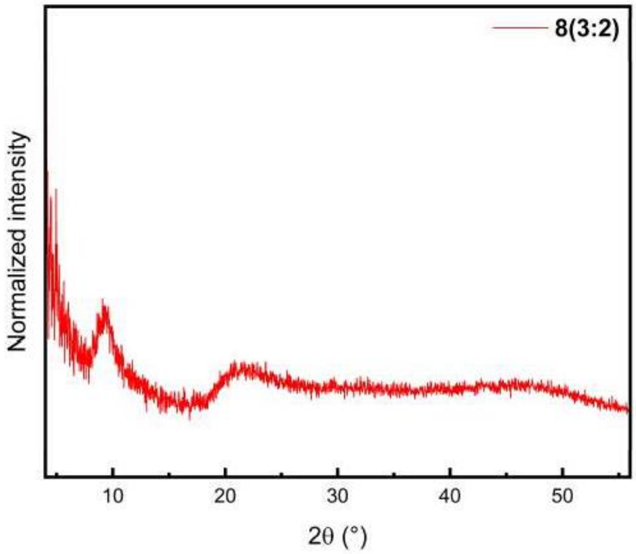

Figure S16. PXRD pattern of **8(3:2)**.

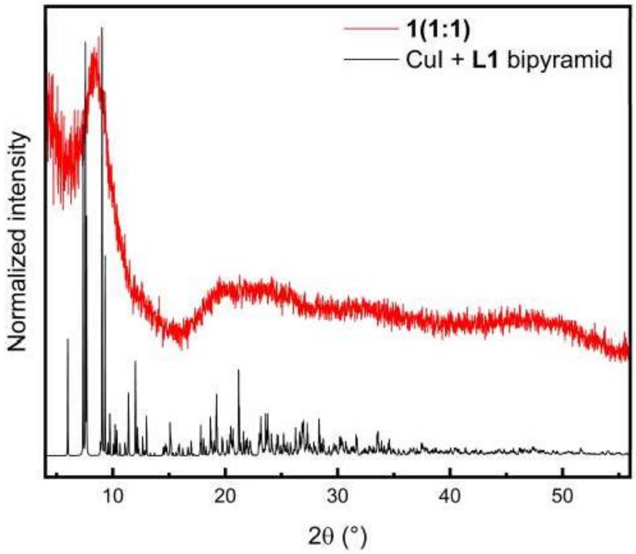

Figure S17. PXRD pattern of **1(1:1)**, compared to the calculated pattern for the bipyramid motif as reported by this work.

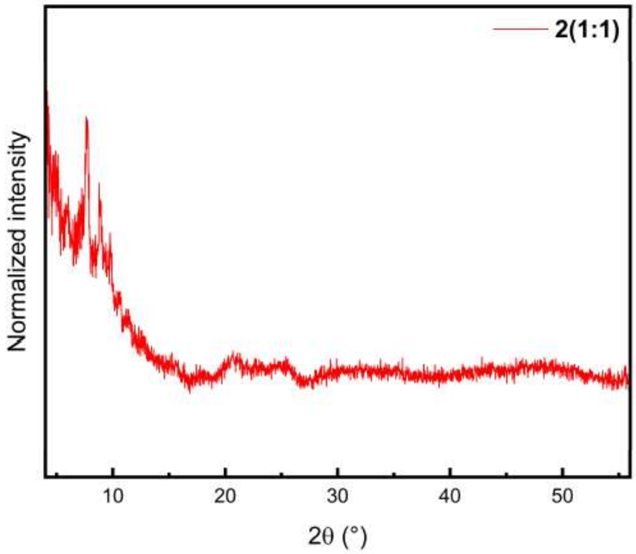

Figure S18. PXRD pattern for **2(1:1)**.

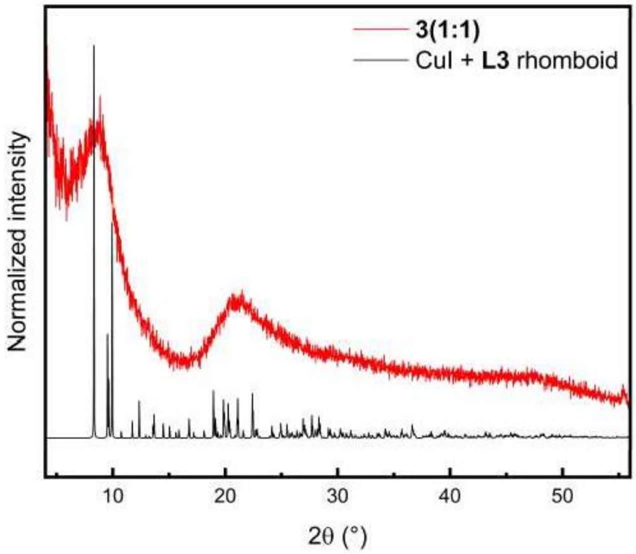

Figure S19. PXRD pattern of **3(1:1)**, compared to the calculated pattern for the rhomboid motif as reported by references [6,8].

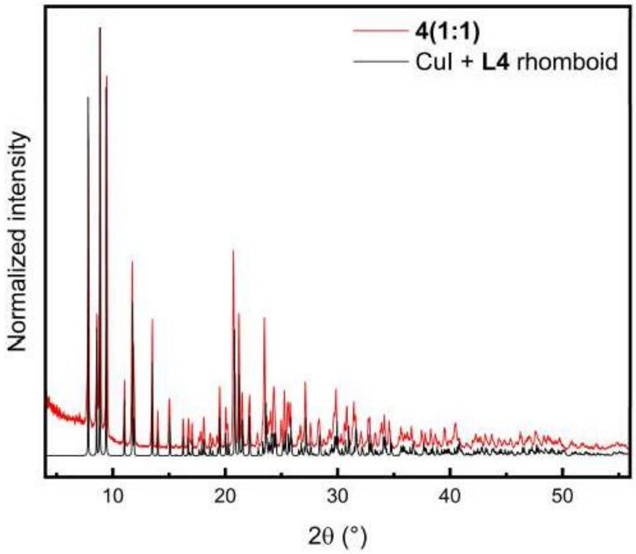

Figure S20. PXRD pattern of **4(1:1)**, compared to the calculated pattern for the rhomboid motif as reported by references [4,5].

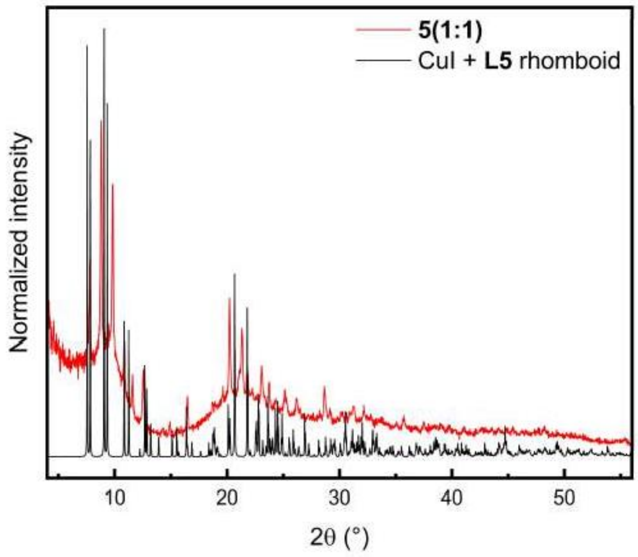

Figure S21. PXRD pattern of **5(1:1)**, compared to the calculated pattern for the rhomboid motif as reported by references [5,6].

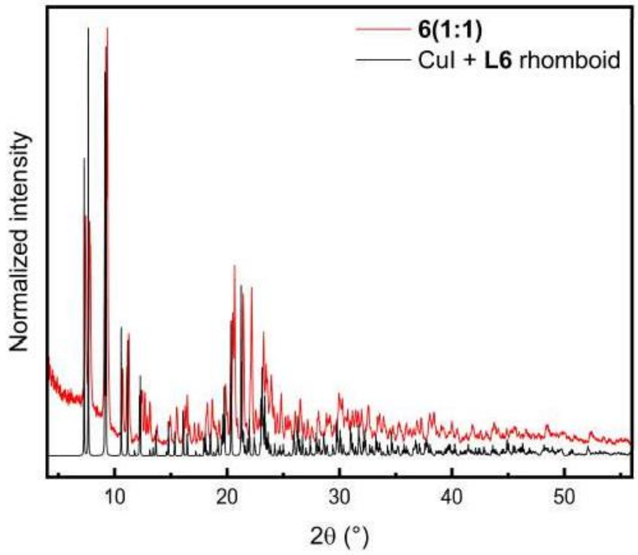

Figure S22. PXRD pattern of **6(1:1)** compared to the calculated pattern for the rhomboid motif as reported by this work.

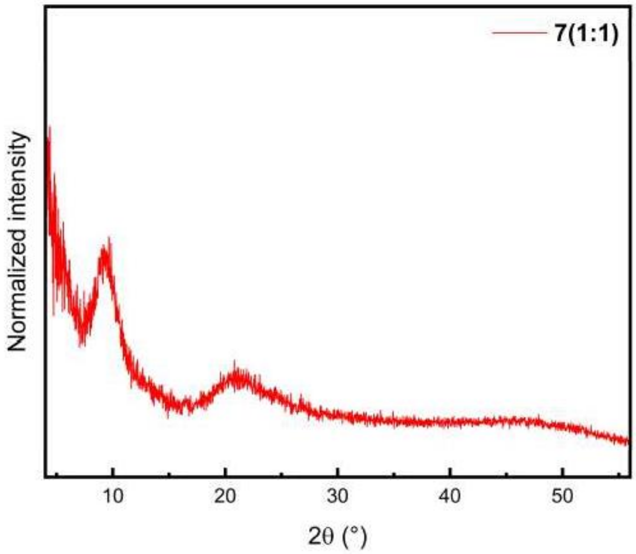

Figure S23. PXRD pattern of **7(1:1)**.

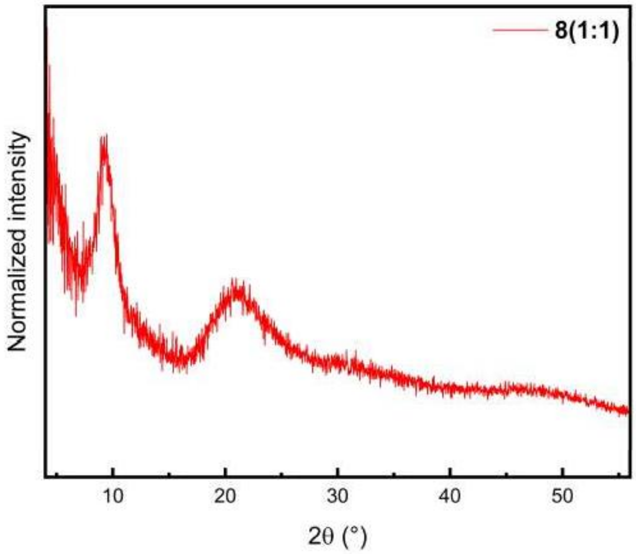

Figure S24. PXRD pattern of **8(1:1)**.

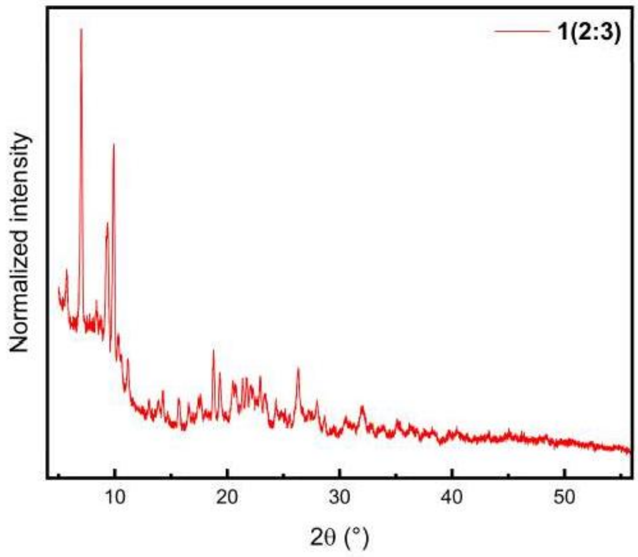

Figure S25. PXRD pattern of **1(2:3)**.

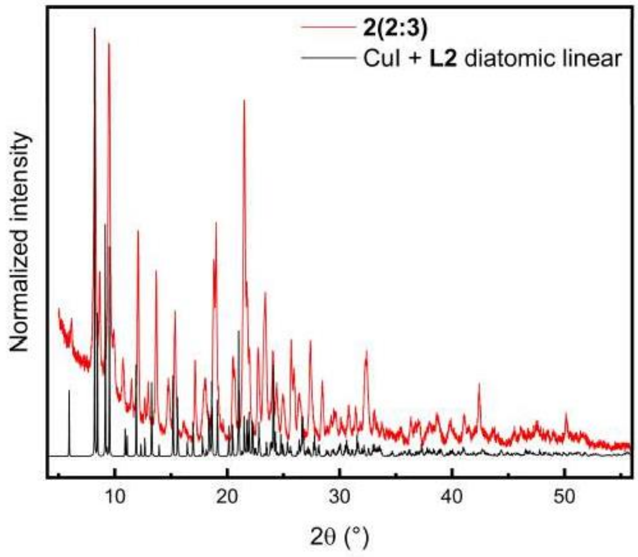

Figure S26. PXRD pattern of **2(2:3)**, compared to the calculated pattern for the diatomic linear motif as reported by references [9,10].

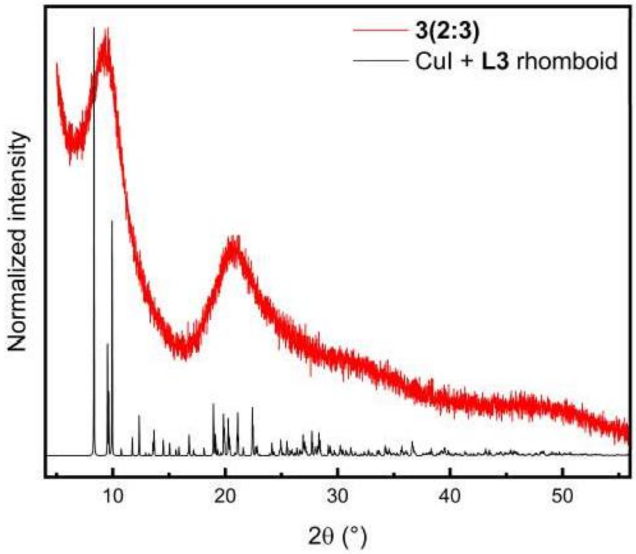

Figure S27. PXRD pattern of **3(2:3)**, compared to the calculated pattern for the rhomboid motif as reported by references [6,8].

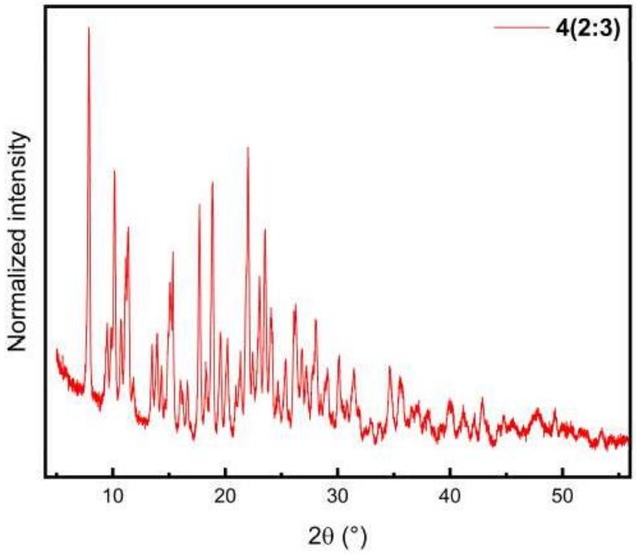

Figure S28. PXRD pattern of **4(2:3)**.

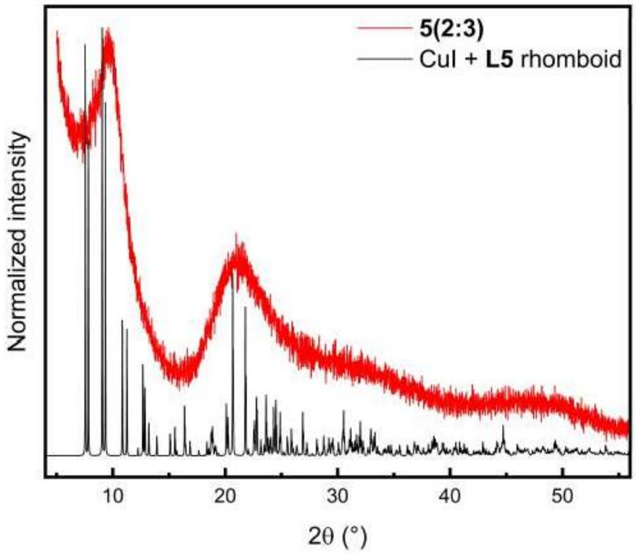

Figure S29. PXRD pattern of **5(2:3)**, compared to the calculated pattern for the rhomboid motif as reported by reference [5,6].

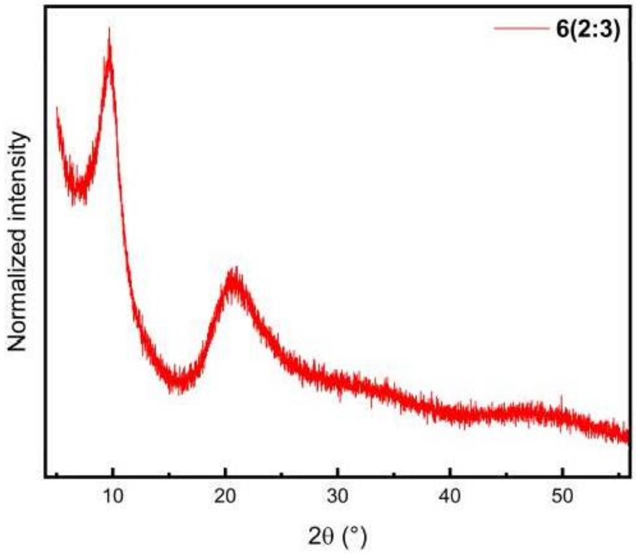

Figure S30. PXRD pattern of **6(2:3)**.

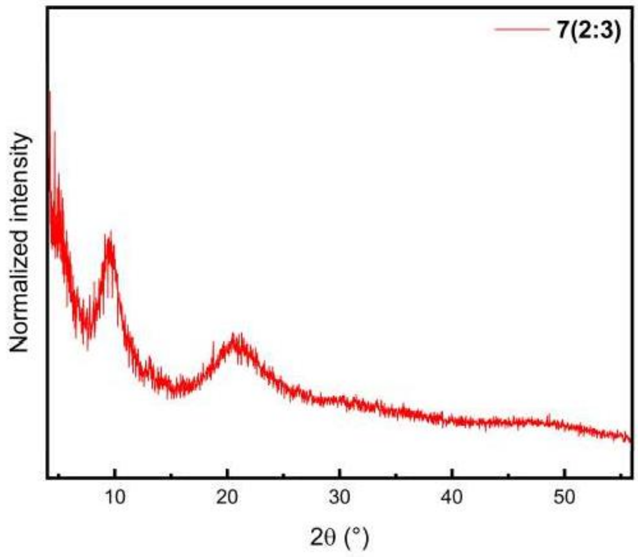

Figure S31. PXRD pattern of **7(2:3)**.

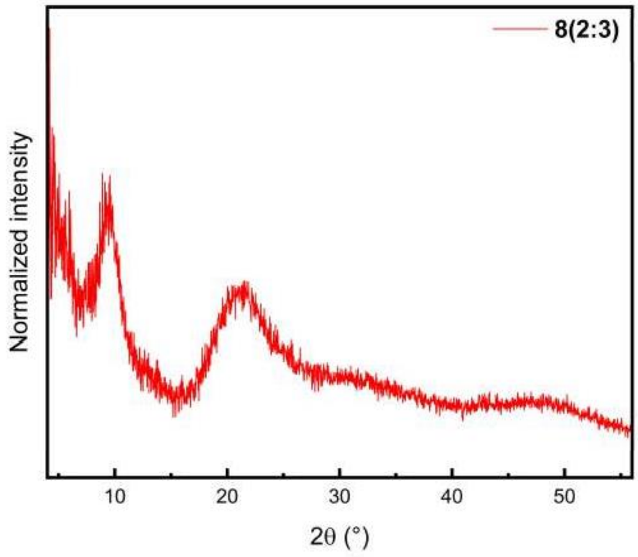

Figure S32. PXRD pattern of **8(2:3)**.

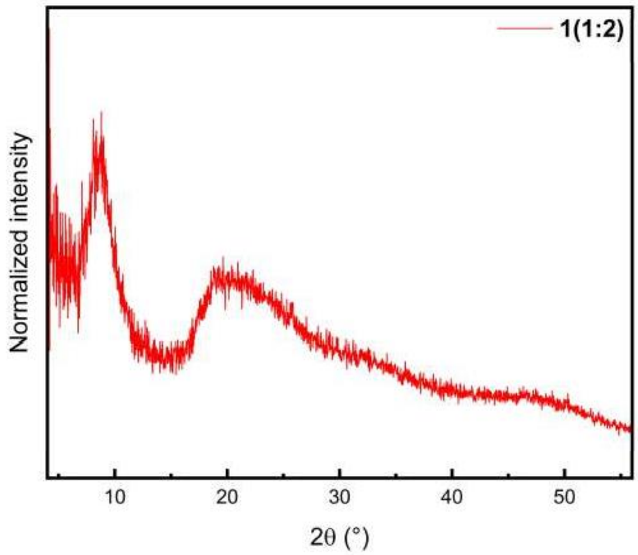

Figure S33. PXRD pattern of **1(1:2)**.

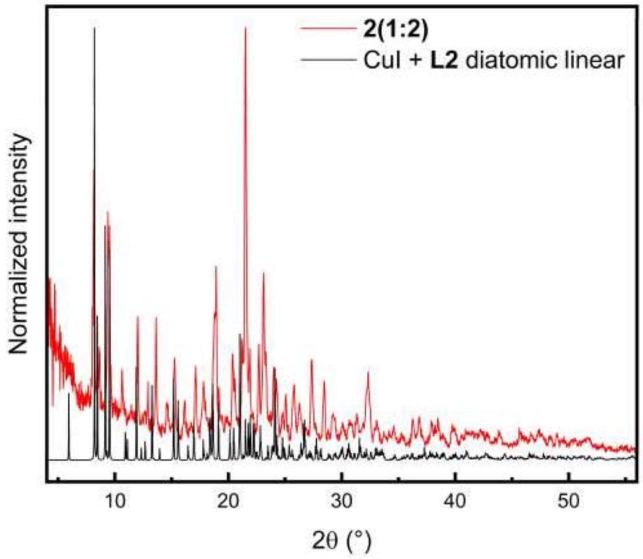

Figure S34. PXRD pattern of **2(1:2)**, compared to th calculated pattern for the diatomic linear motif as reported by references [9,10].

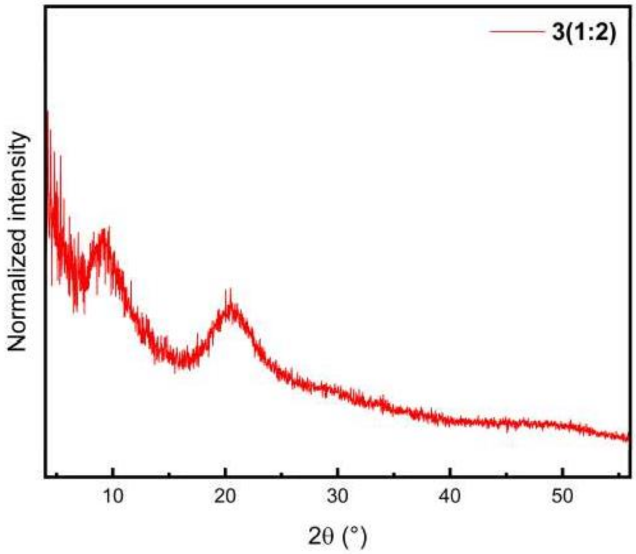

Figure S35. PXRD pattern of **3(1:2)**.

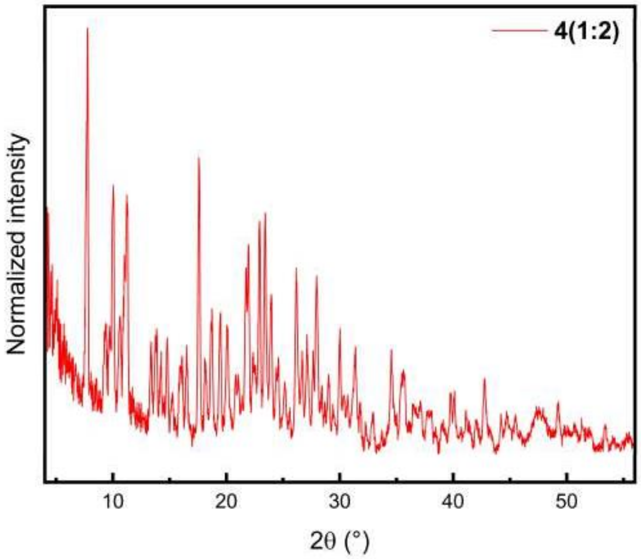

Figure S36. PXRD pattern of **4(1:2)**.

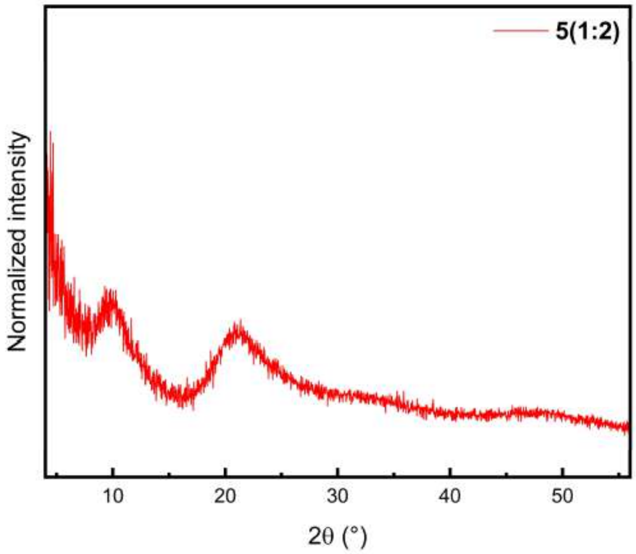

Figure S37. PXRD pattern of **5(1:2)**.

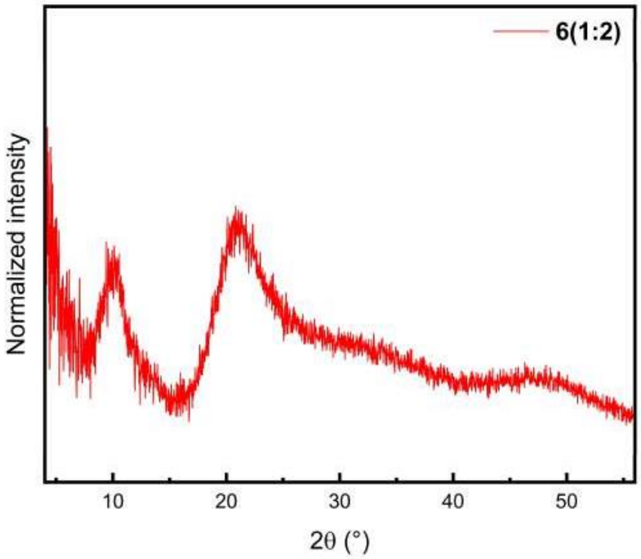

Figure S38. PXRD pattern of **6(1:2)**.

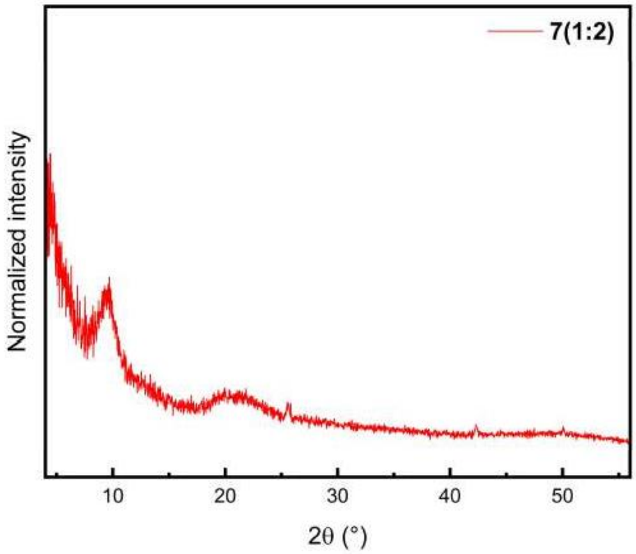

Figure S39. PXRD pattern of **7(1:2)**.

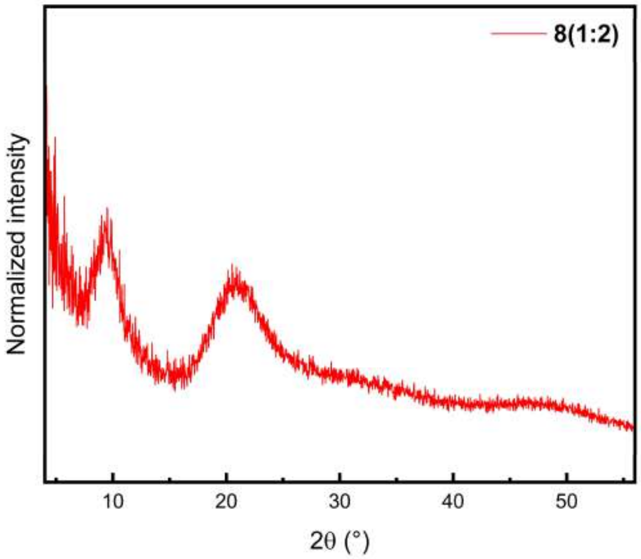

Figure S40. PXRD pattern of **8(1:2)**.

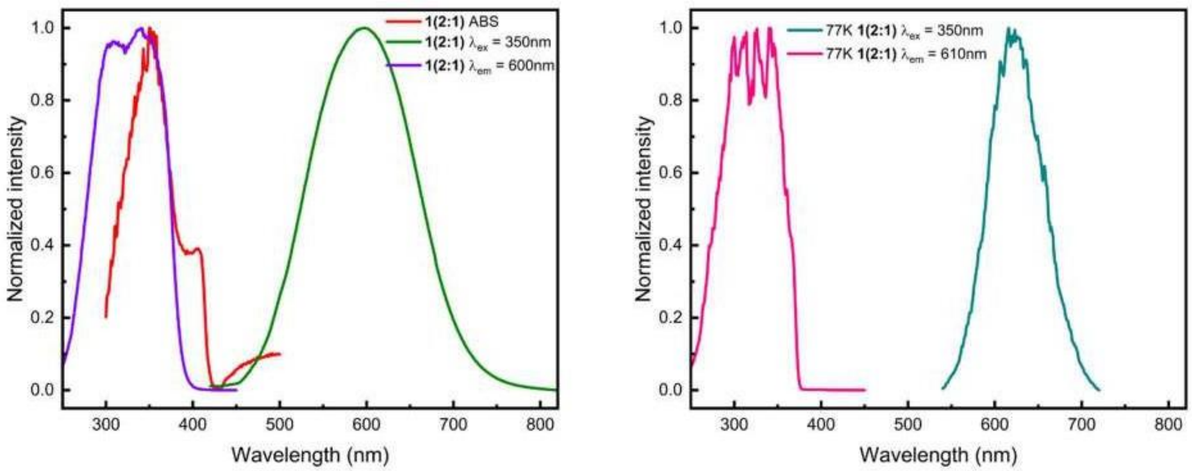

Figure S41. UV-Vis absorption and PL spectra of **1(2:1)**. Left : 293K. Right : 77K.

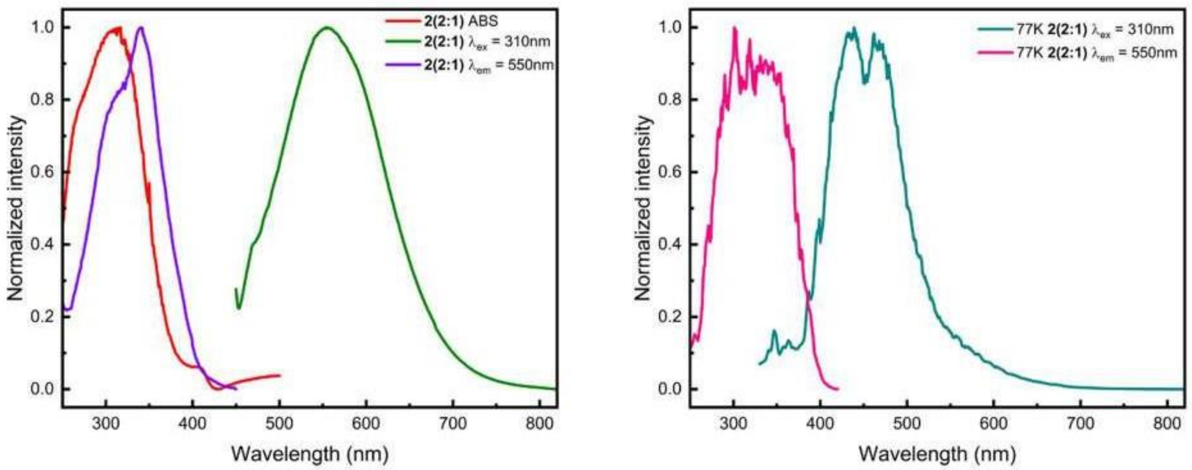

Figure S42. UV-Vis absorption and PL spectra of **2(2:1)**. Left : 293K. Right : 77K.

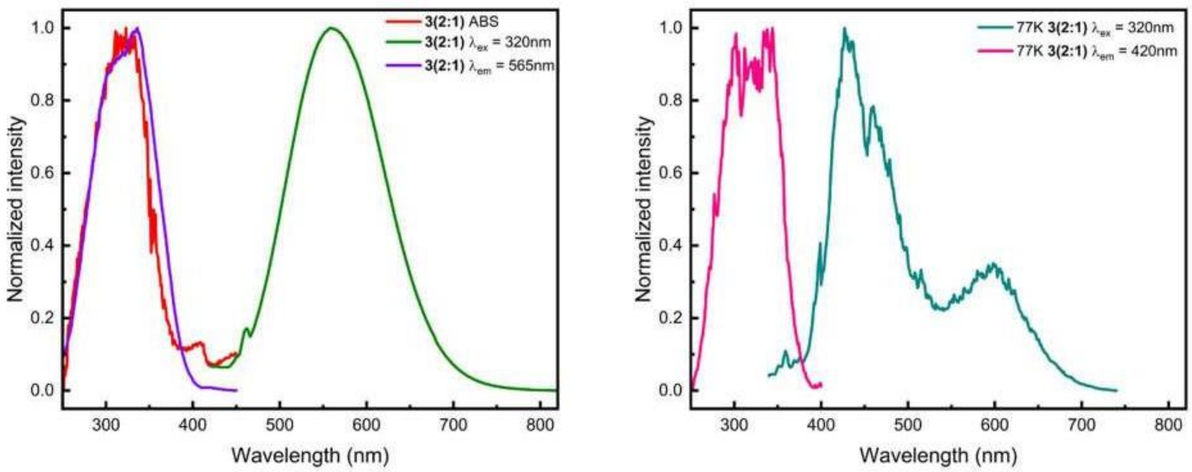

Figure S43. UV-Vis absorption and PL spectra of **3(2:1)**. Left : 293K. Right : 77K

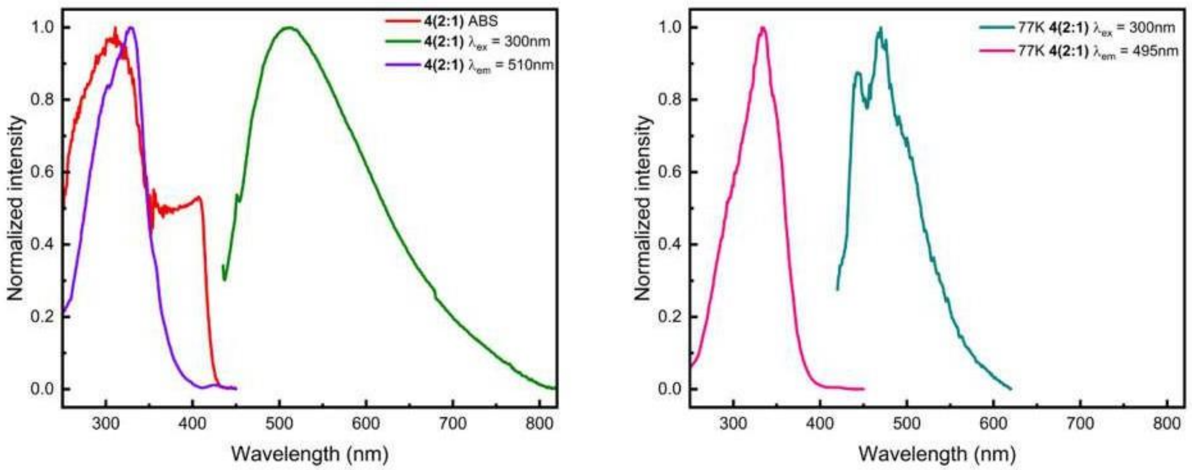

Figure S44. UV-Vis absorption and PL spectra of **4(2:1)**. Left : 293K. Right : 77K

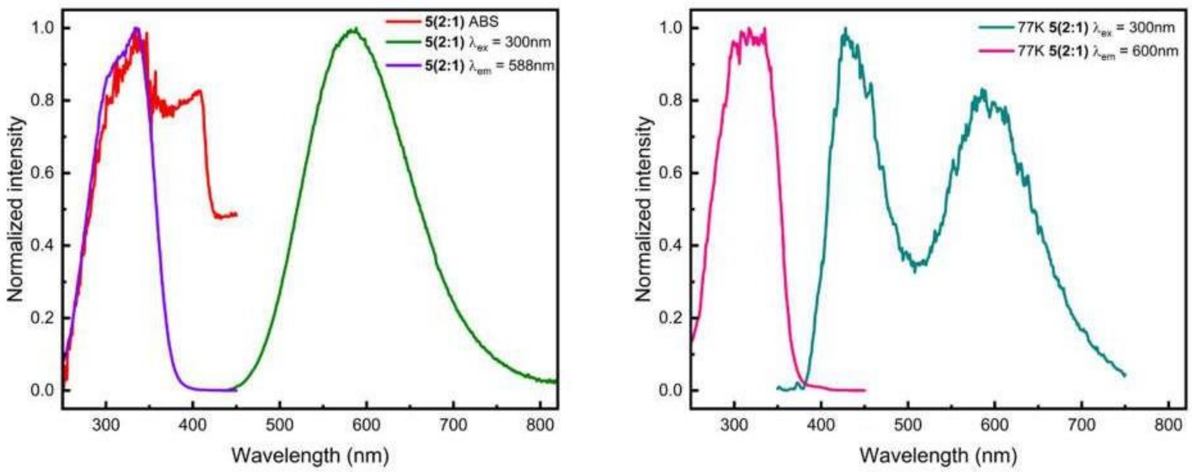

Figure S45. UV-Vis absorption and PL spectra of **5(2:1)**. Left : 293K. Right : 77K

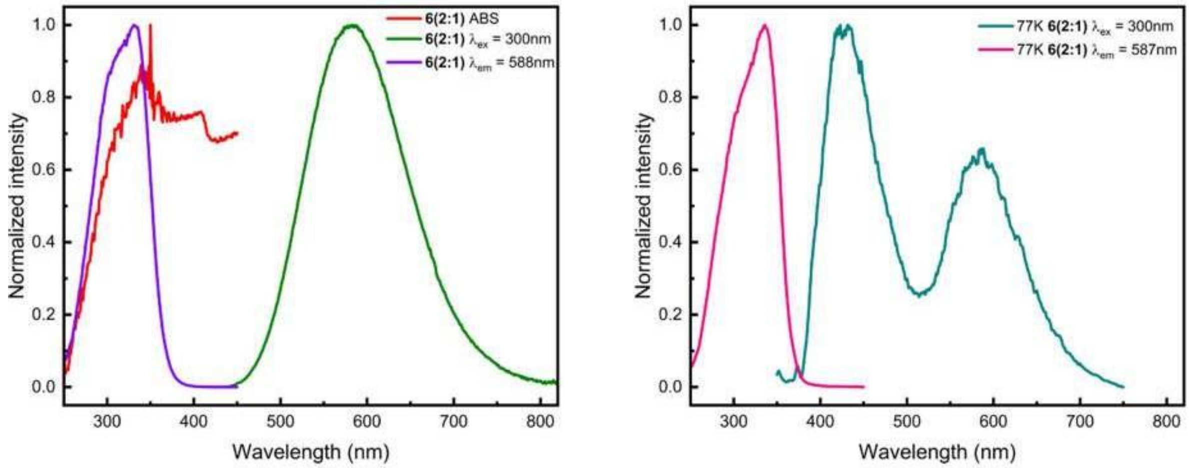

Figure S46. UV-Vis absorption and PL spectra of **6(2:1)**. Left : 293K. Right : 77K

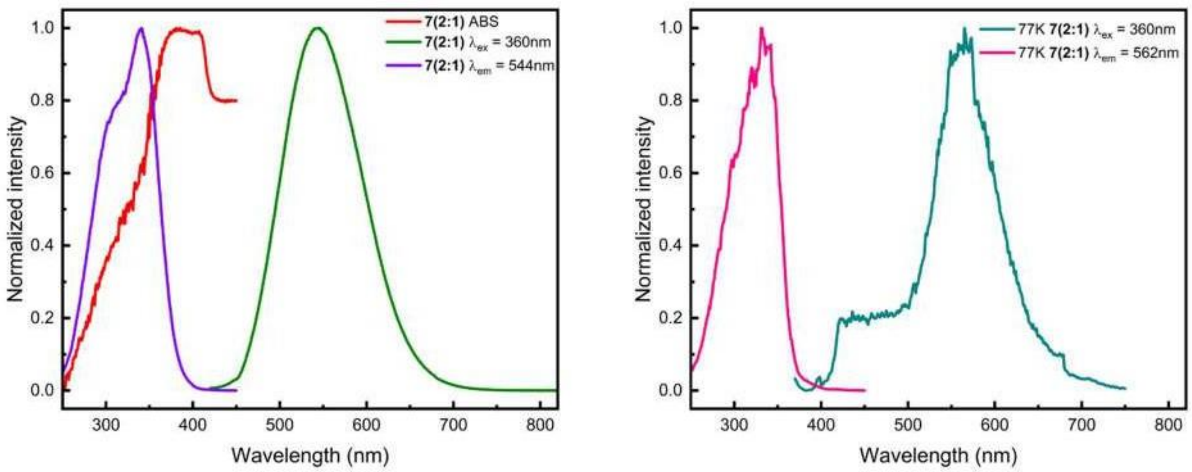

Figure S47. UV-Vis absorption and PL spectra of **7(2:1)**. Left : 293K. Right : 77K

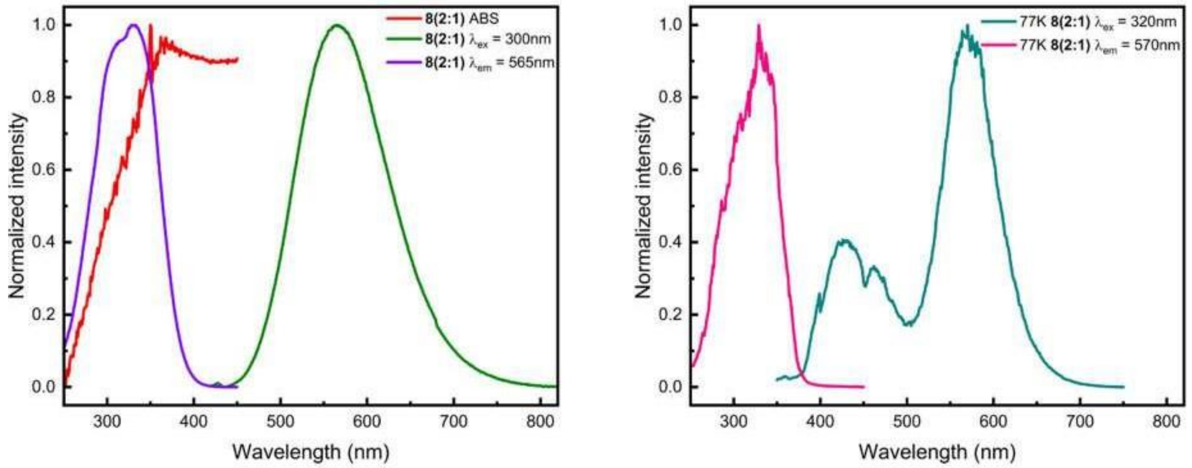

Figure S48. UV-Vis absorption and PL spectra of **8(2:1)**. Left : 293K. Right : 77K

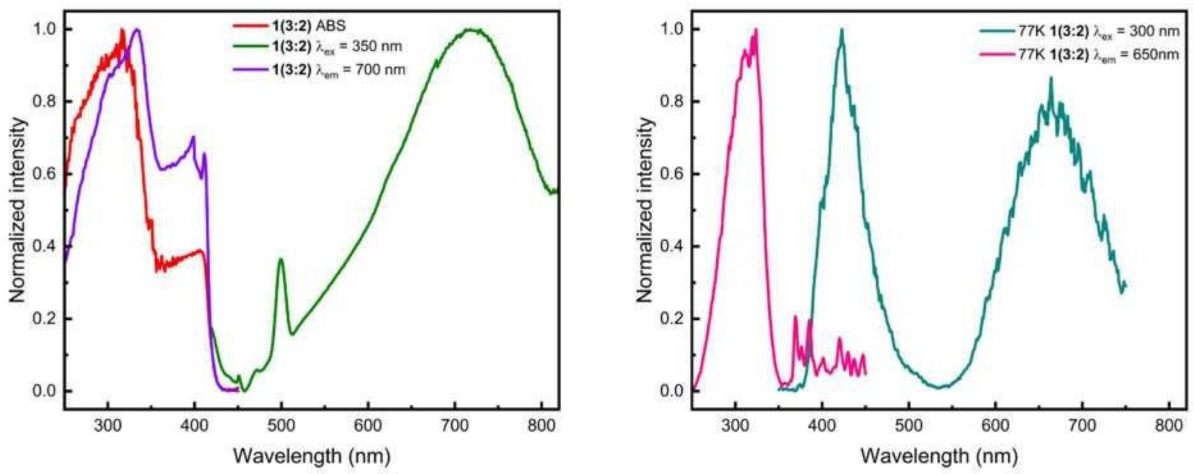

Figure S49. UV-Vis absorption and PL spectra of **1(3:2)**. Left : 293K. Right : 77K

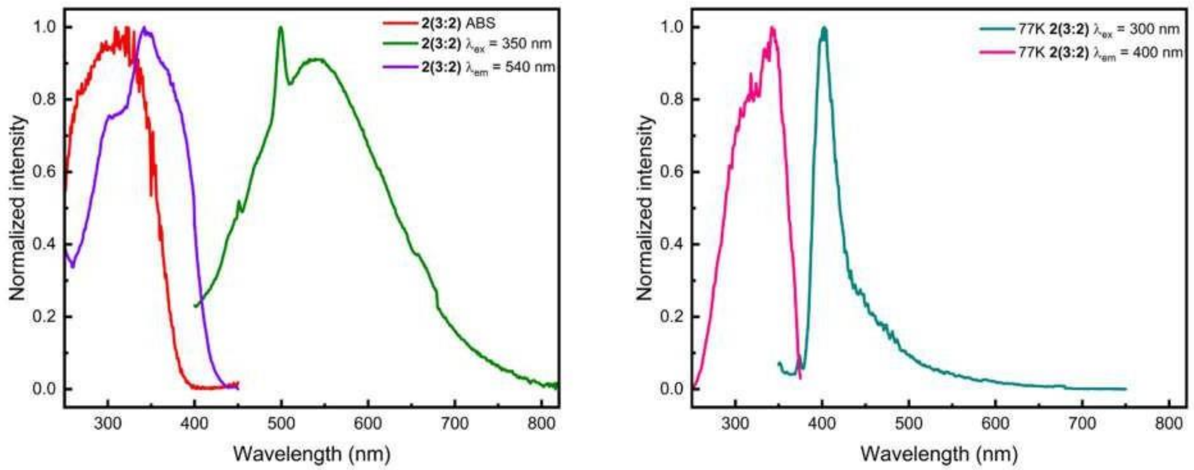

Figure S50. UV-Vis absorption and PL spectra of **2(3:2)**. Left : 293K. Right : 77K

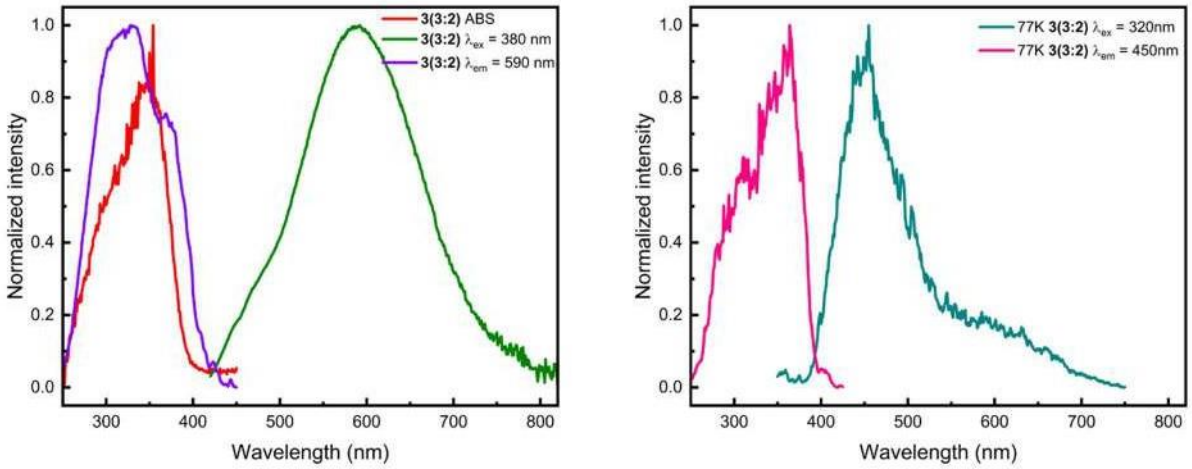

Figure S51. UV-Vis absorption and PL spectra of **3(3:2)**. Left : 293K. Right : 77K

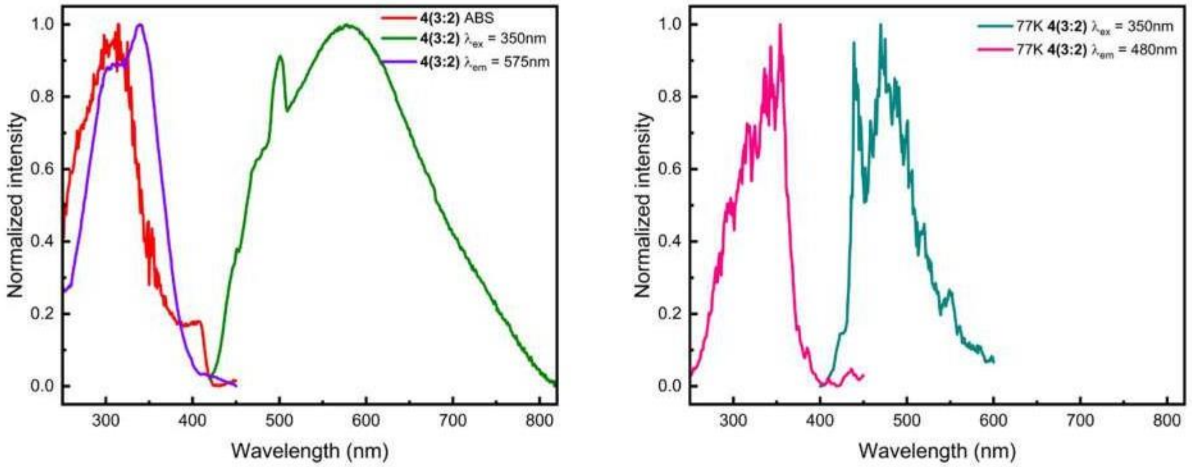

Figure S52. UV-Vis absorption and PL spectra of **4(3:2)**. Left : 293K. Right : 77K

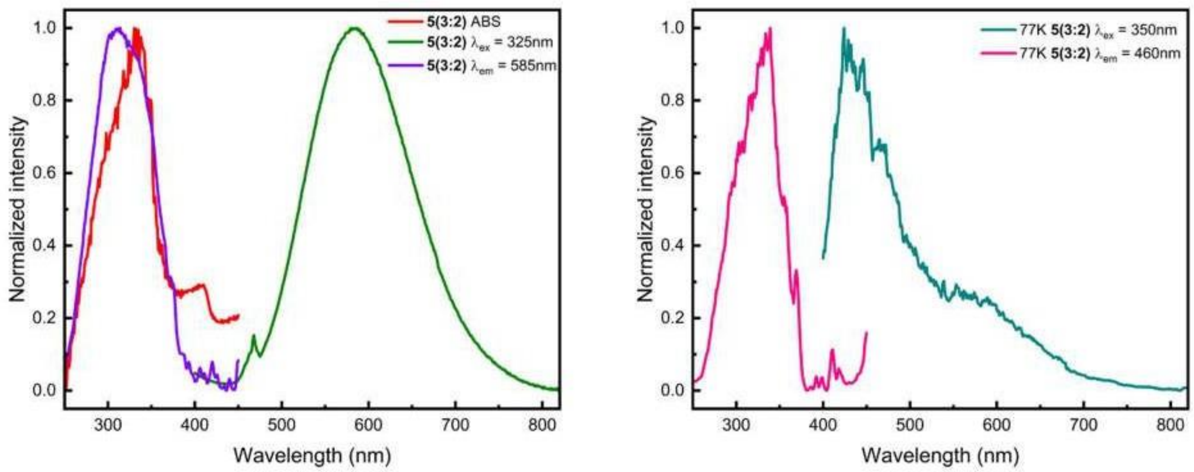

Figure S53. UV-Vis absorption and PL spectra of **5(3:2)**. Left : 293K. Right : 77K

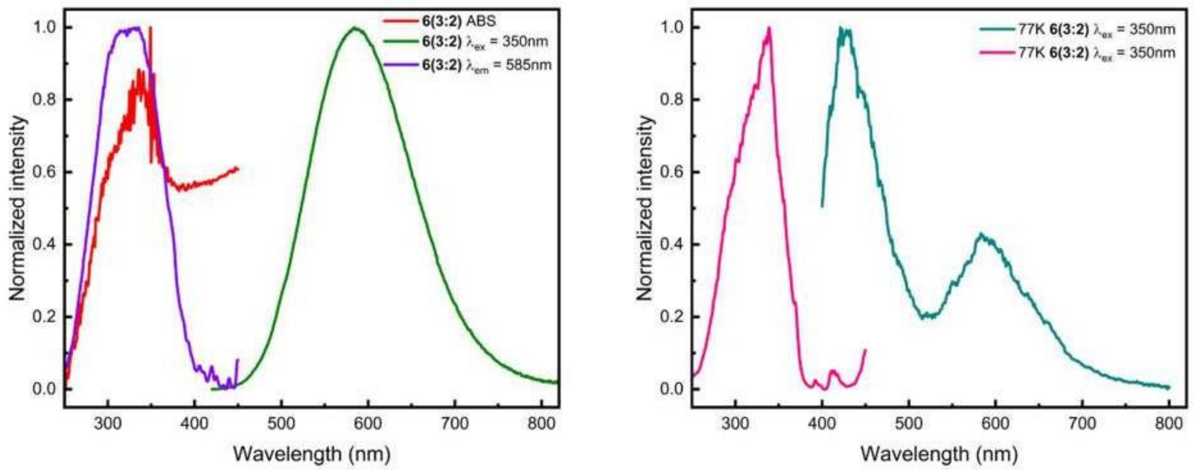

Figure S54. UV-Vis absorption and PL spectra of **6(3:2)**. Left : 293K. Right : 77K

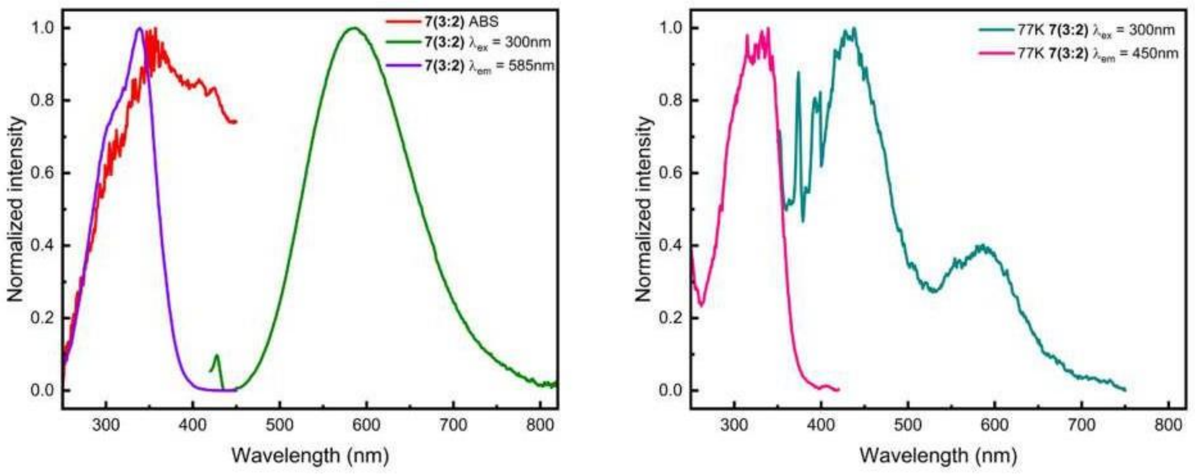

Figure S55. UV-Vis absorption and PL spectra of **7(3:2)**. Left : 293K. Right : 77K

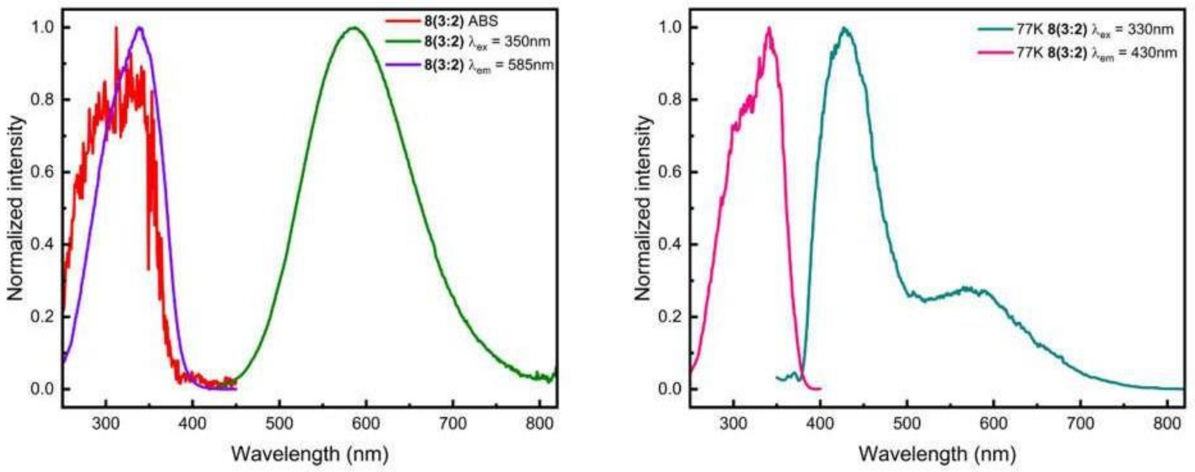

Figure S56. UV-Vis absorption and PL spectra of **8(3:2)**. Left : 293K. Right : 77K

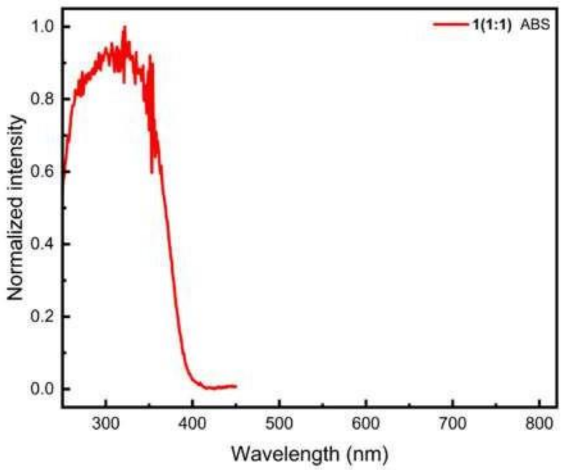

Figure S57. UV-Vis absorption and PL spectra of **1(1:1)**. Left : 293K. Right : 77K

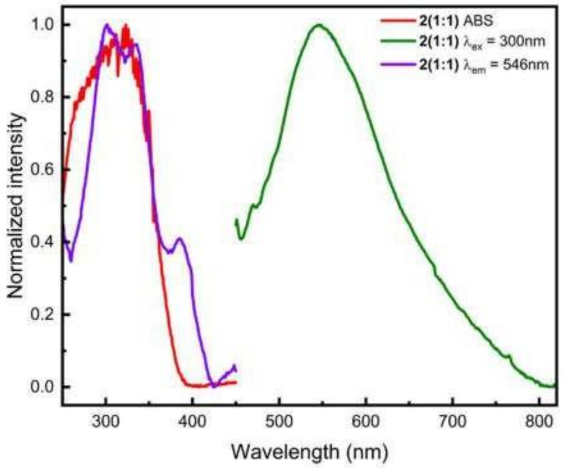

Figure S58. UV-Vis absorption and PL spectra of **2(1:1)**. Left : 293K. Right : 77K

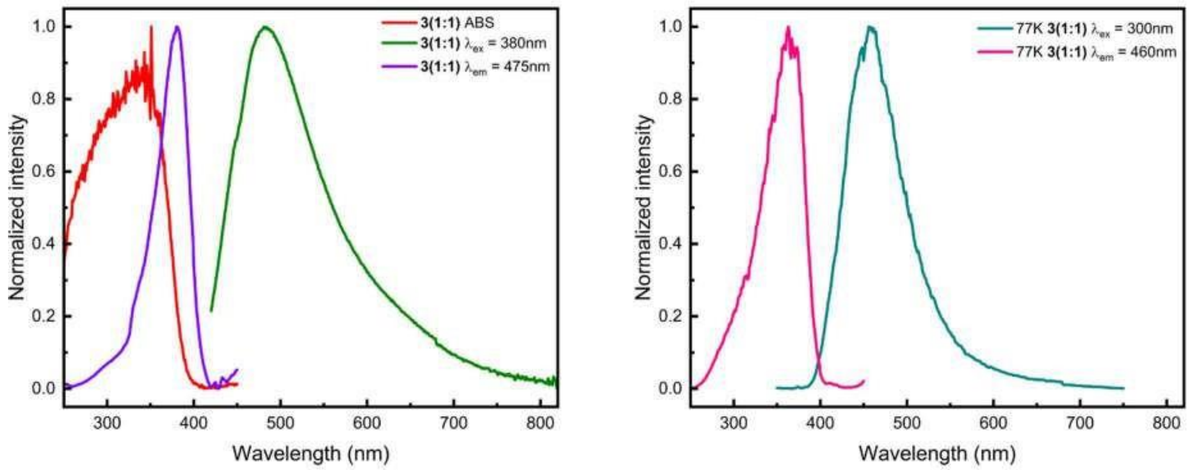

Figure S59. UV-Vis absorption and PL spectra of **3(1:1)**. Left : 293K. Right : 77K

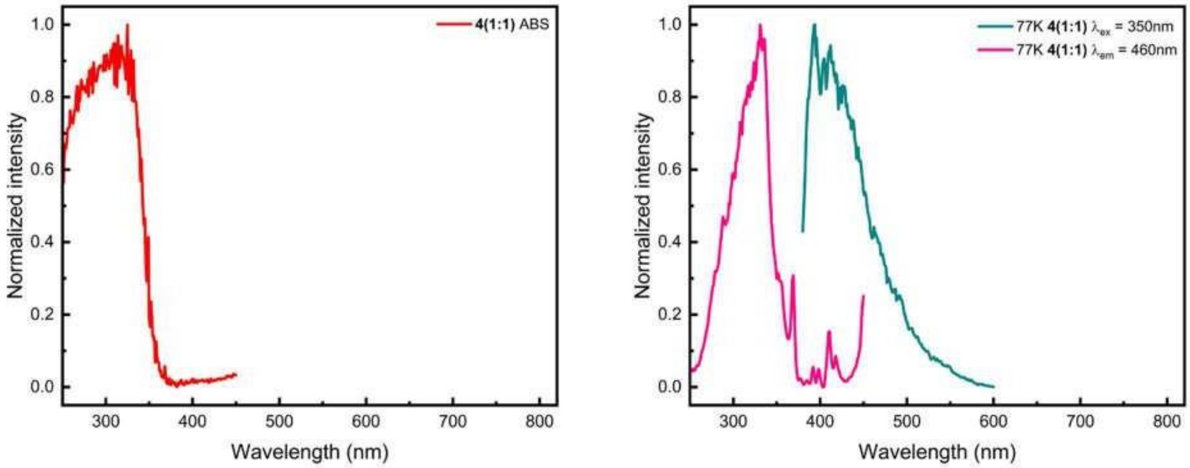

Figure S60. UV-Vis absorption and PL spectra of **4(1:1)**. Left : 293K. Right : 77K

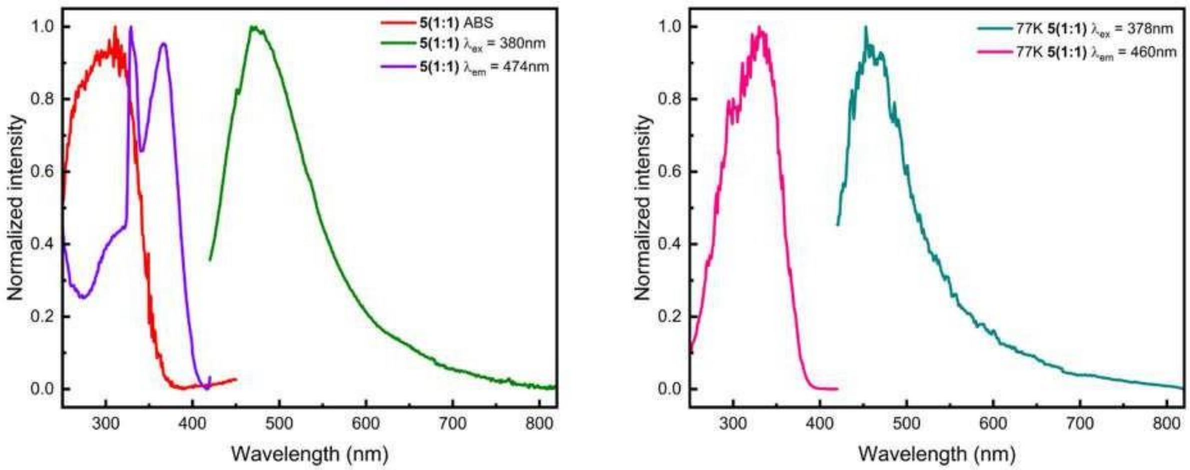

Figure S61. UV-Vis absorption and PL spectra of **5(1:1)**. Left : 293K. Right : 77K

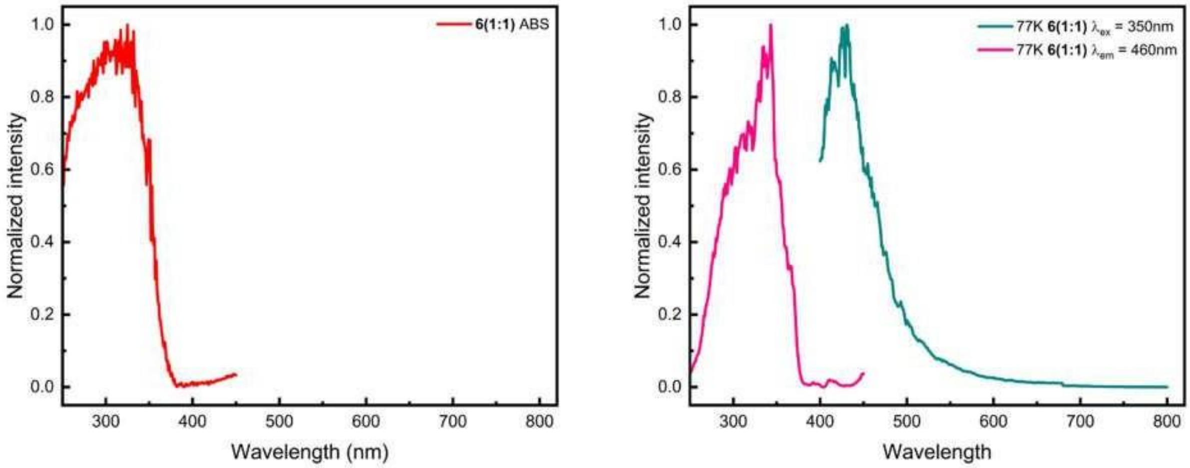

Figure S62. UV-Vis absorption and PL spectra of **6(1:1)**. Left : 293K. Right : 77K

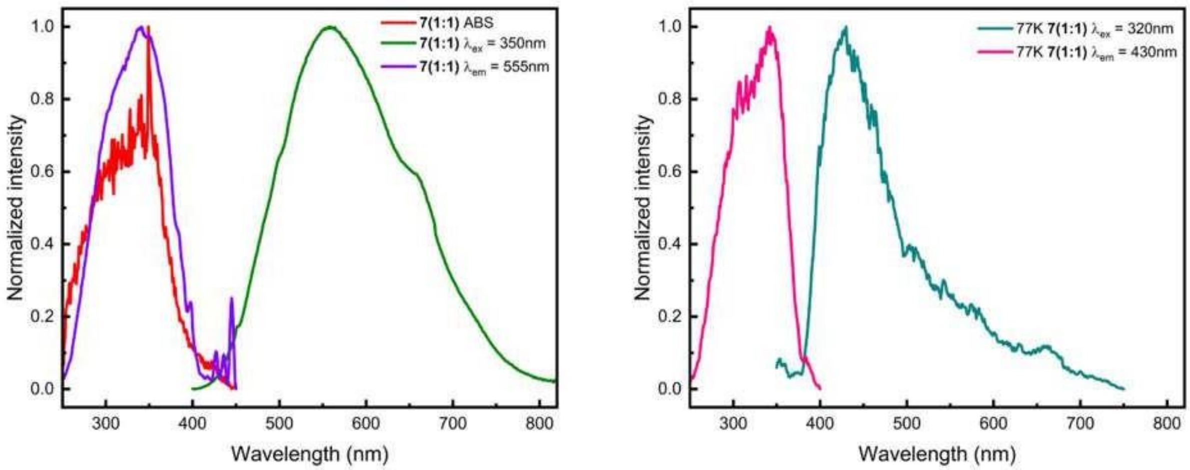

Figure S63. UV-Vis absorption and PL spectra of **7(1:1)**. Left : 293K. Right : 77K

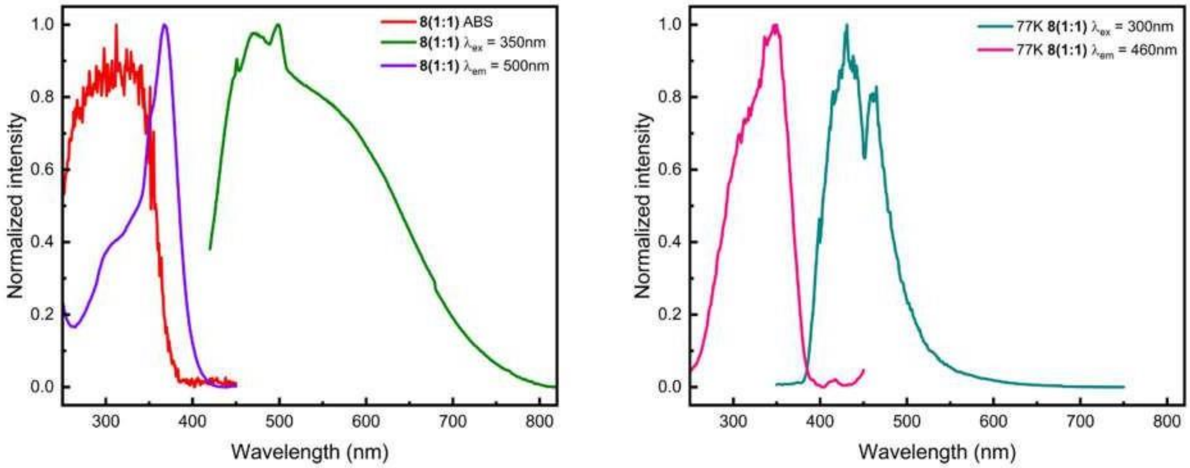

Figure S64. UV-Vis absorption and PL spectra of **8(1:1)**. Left : 293K. Right : 77K

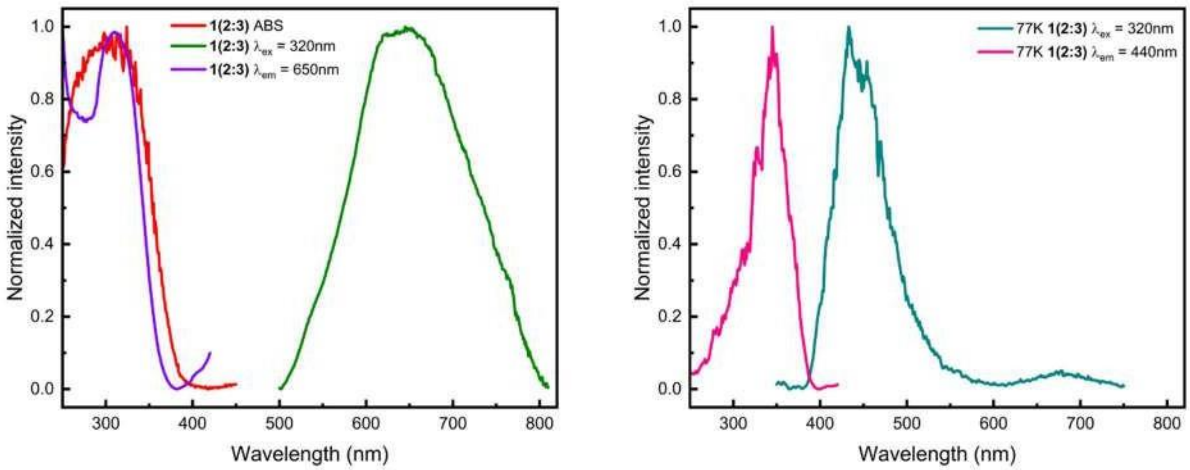

Figure S65. UV-Vis absorption and PL spectra of **1(2:3)**. Left : 293K. Right : 77K

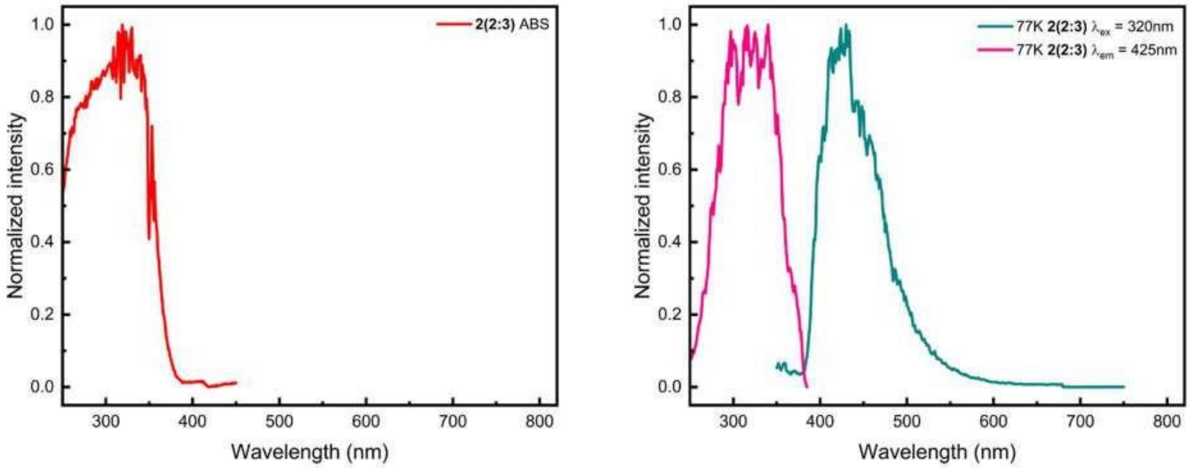

Figure S66. UV-Vis absorption and PL spectra of **2(2:3)**. Left : 293K. Right : 77K

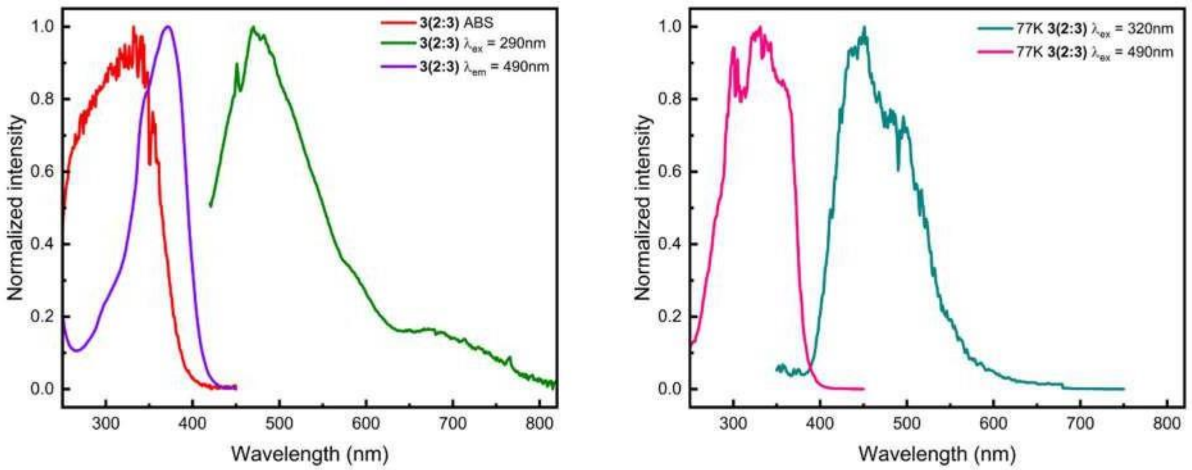

Figure S67. UV-Vis absorption and PL spectra of **3(2:3)**. Left : 293K. Right : 77K

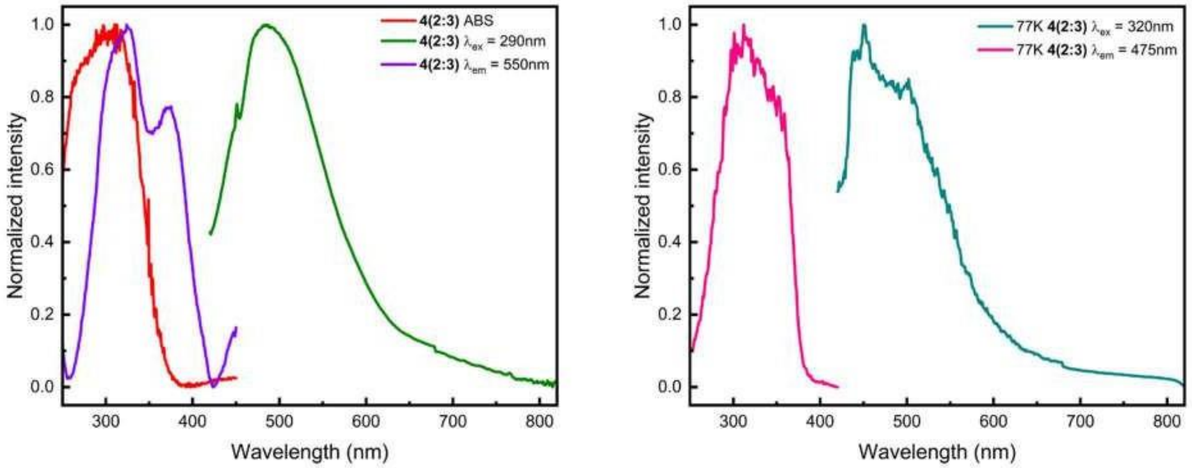

Figure S68. UV-Vis absorption and PL spectra of **4(2:3)**. Left : 293K. Right : 77K

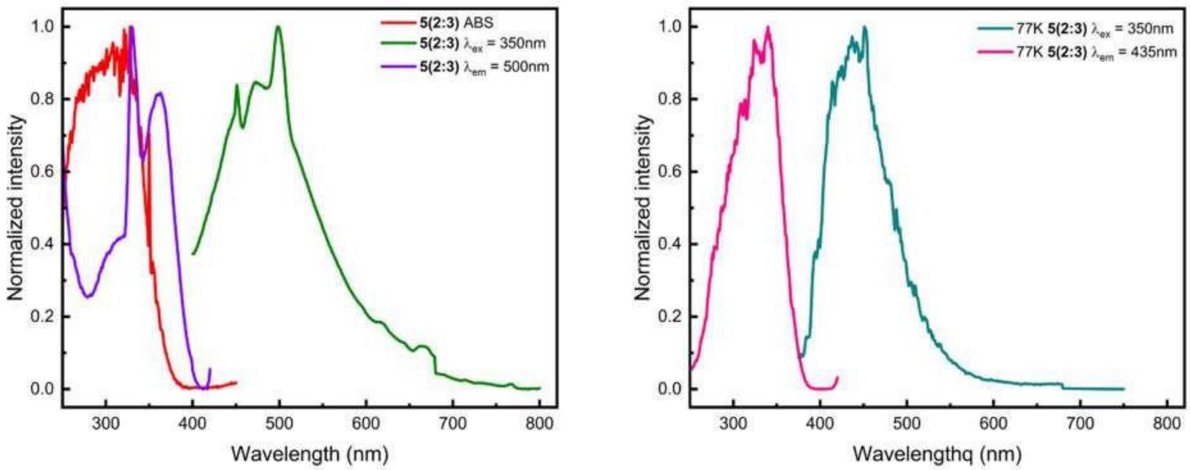

Figure S69. UV-Vis absorption and PL spectra of **5(2:3)**. Left : 293K. Right : 77K

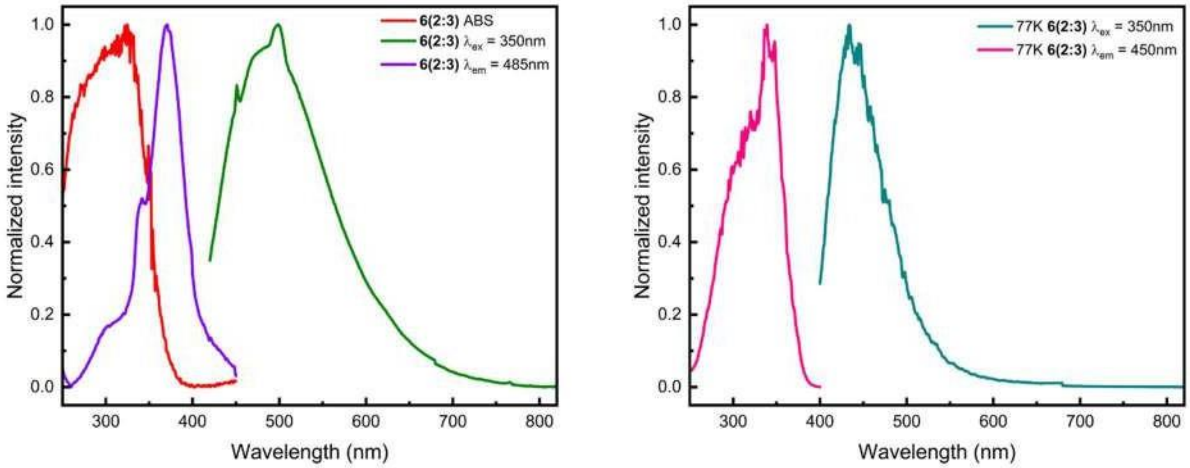

Figure S70. UV-Vis absorption and PL spectra of **6(2:3)**. Left : 293K. Right : 77K

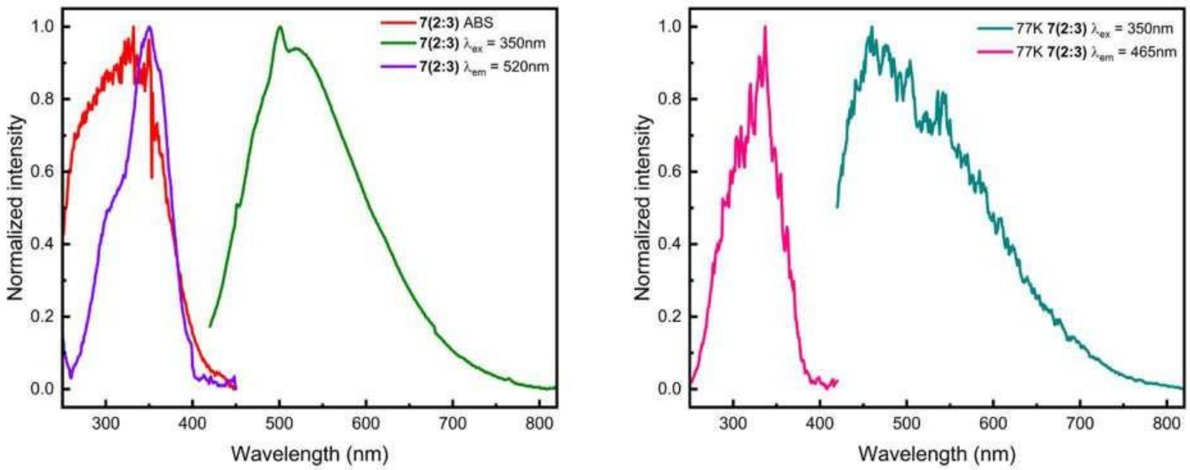

Figure S71. UV-Vis absorption and PL spectra of **7(2:3)**. Left : 293K. Right : 77K

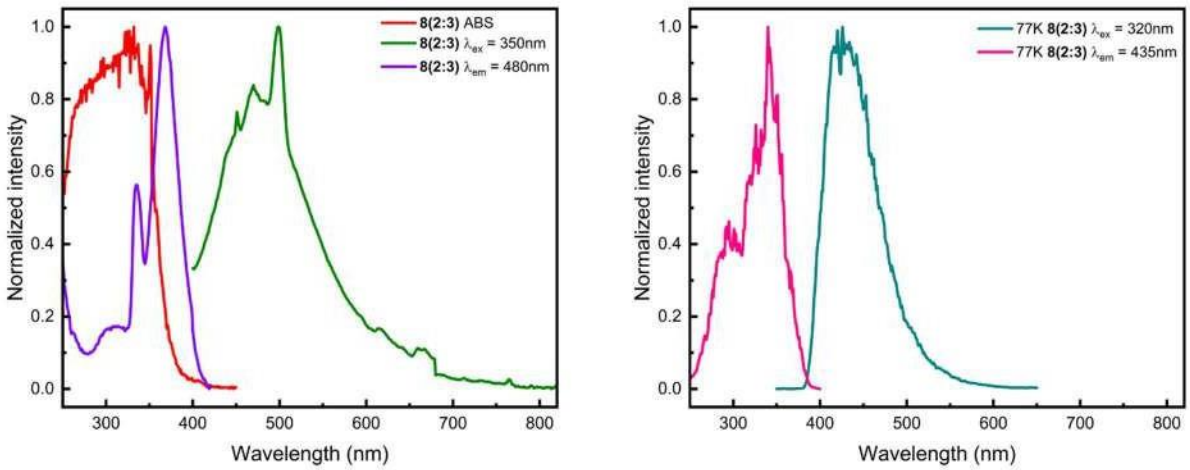

Figure S72. UV-Vis absorption and PL spectra of **8(2:3)**. Left : 293K. Right : 77K

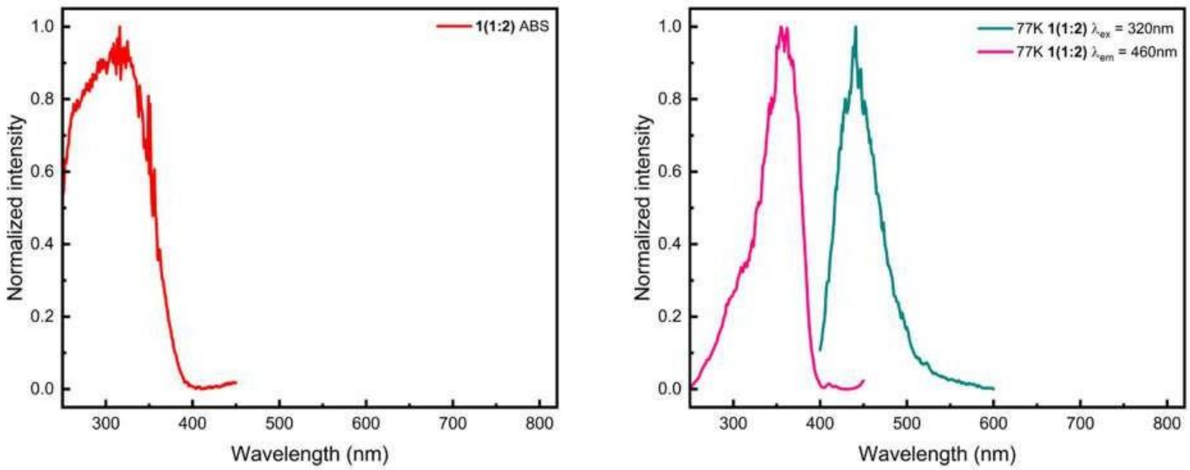

Figure S73. UV-Vis absorption and PL spectra of **1(1:2)**. Left : 293K. Right : 77K

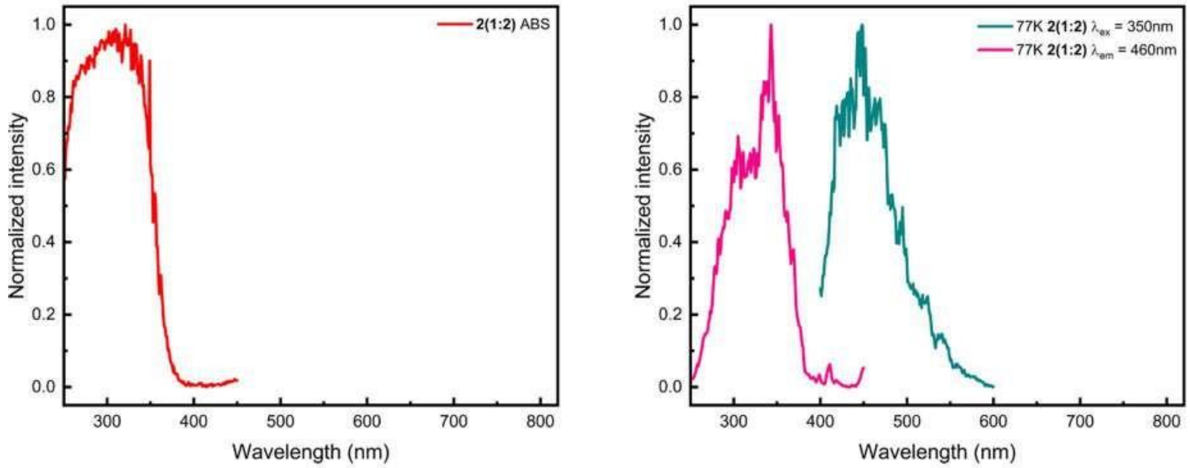

Figure S74. UV-Vis absorption and PL spectra of **2(1:2)**. Left : 293K. Right : 77K

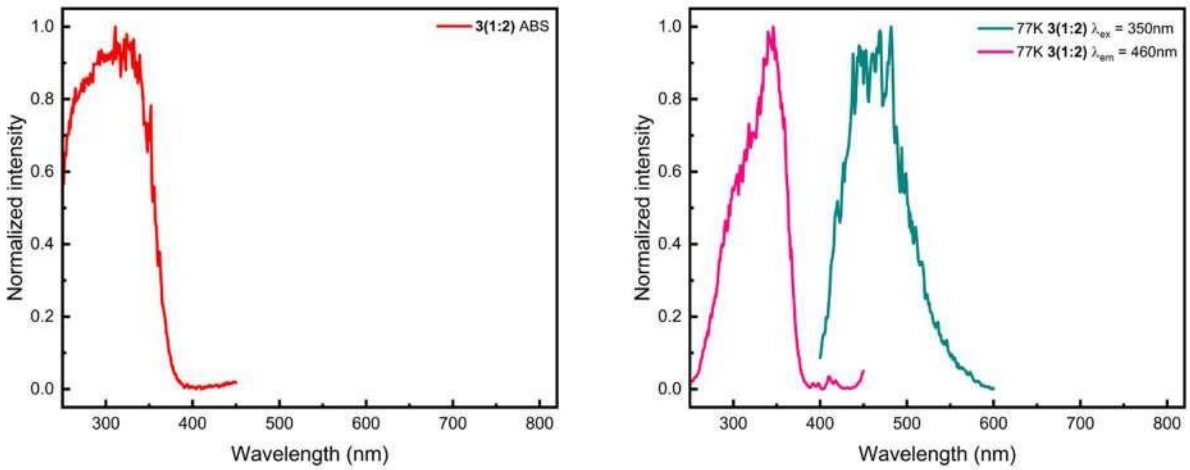

Figure S75. UV-Vis absorption and PL spectra of **3(1:2)**. Left : 293K. Right : 77K

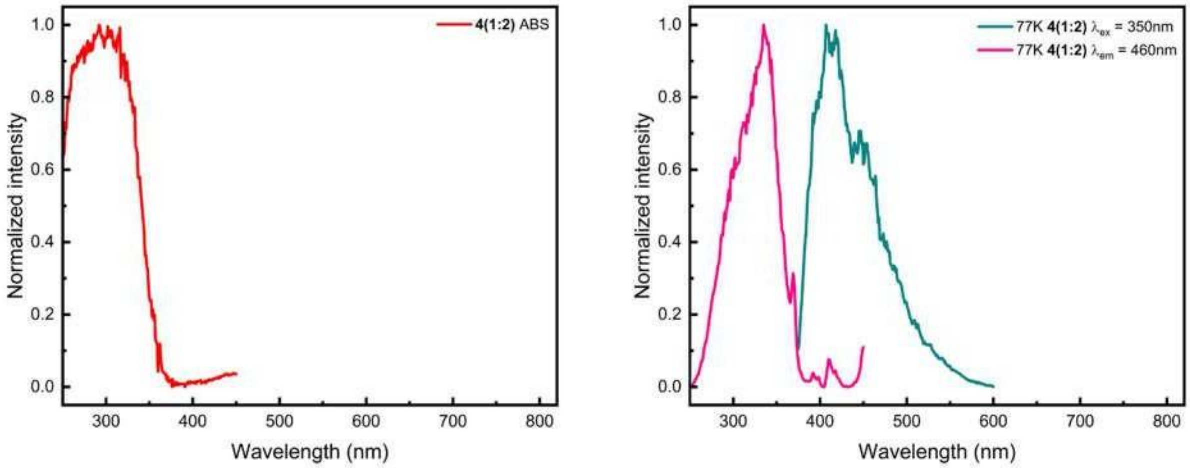

Figure S76. UV-Vis absorption and PL spectra of **4(1:2)**. Left : 293K. Right : 77K

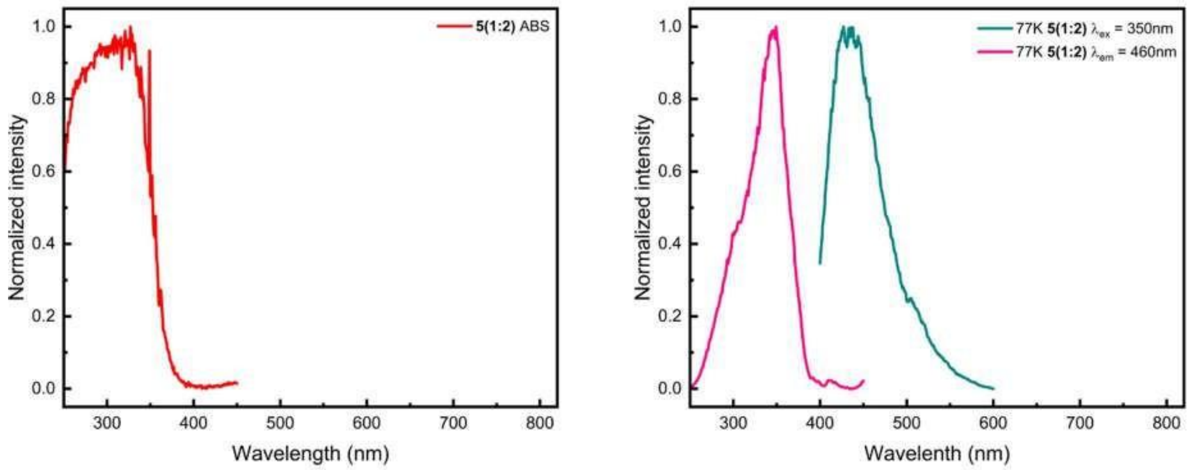

Figure S77. UV-Vis absorption and PL spectra of **5(1:2)**. Left : 293K. Right : 77K

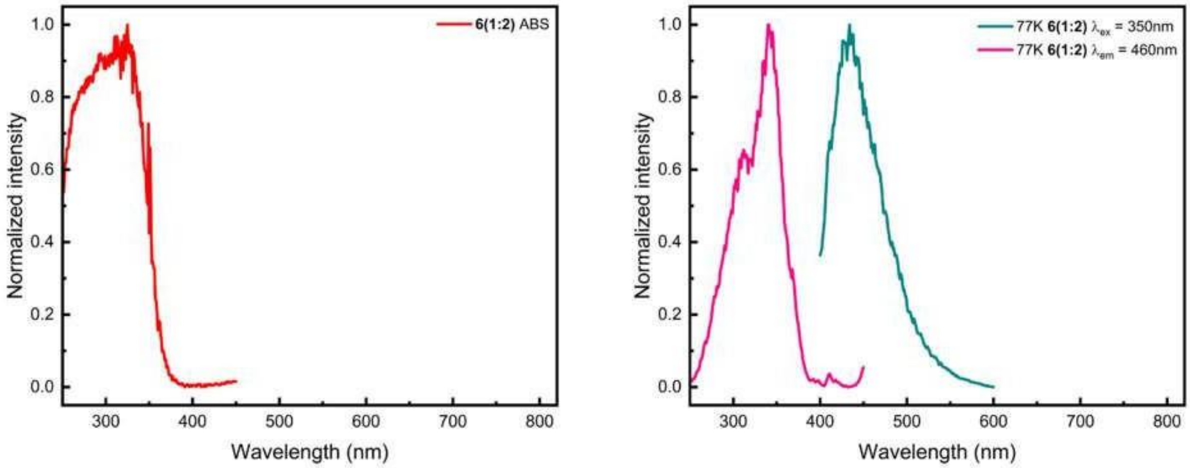

Figure S78. UV-Vis absorption and PL spectra of **6(1:2)**. Left : 293K. Right : 77K

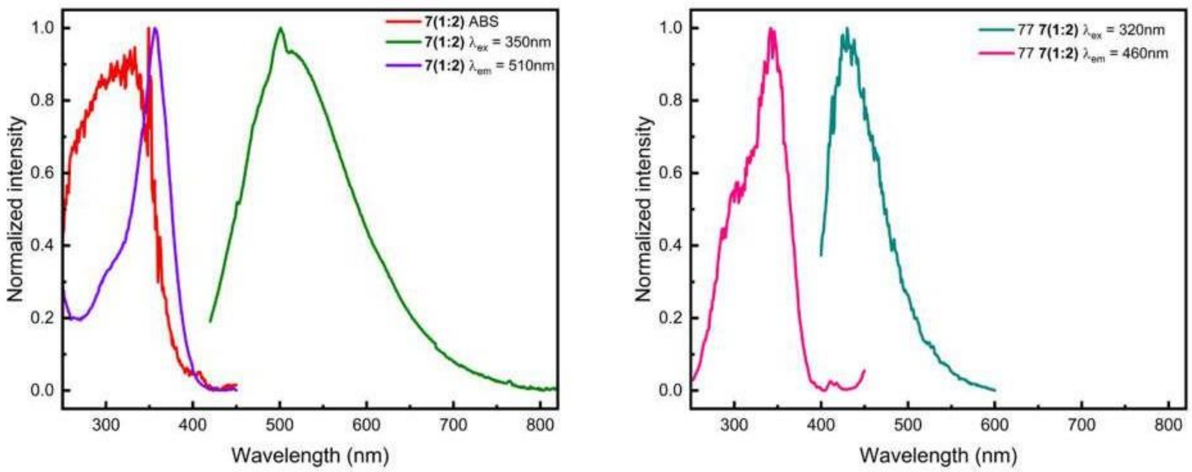

Figure S79. UV-Vis absorption and PL spectra of **7(1:2)**. Left : 293K. Right : 77K

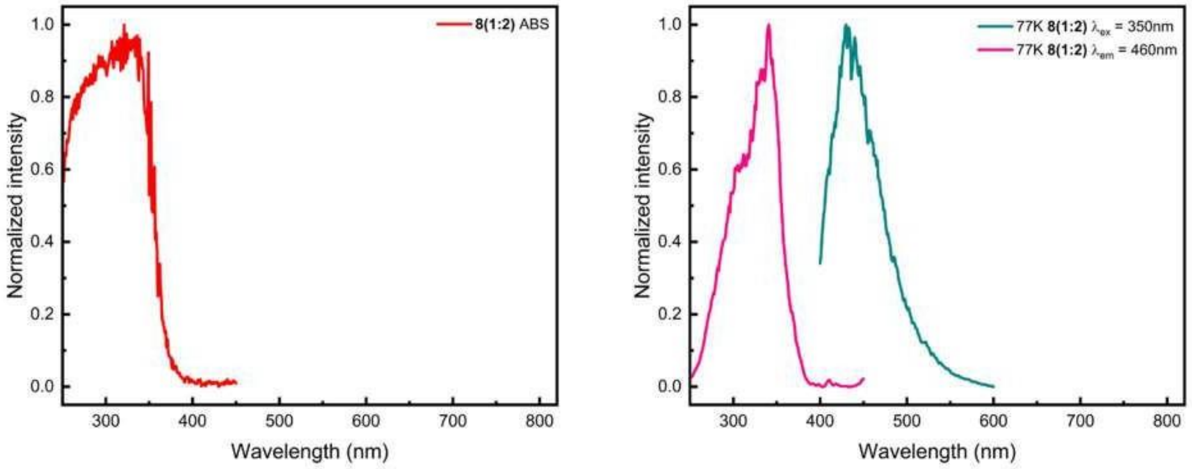

Figure S80. UV-Vis absorption and PL spectra of **8(1:2)**. Left : 293K. Right : 77K

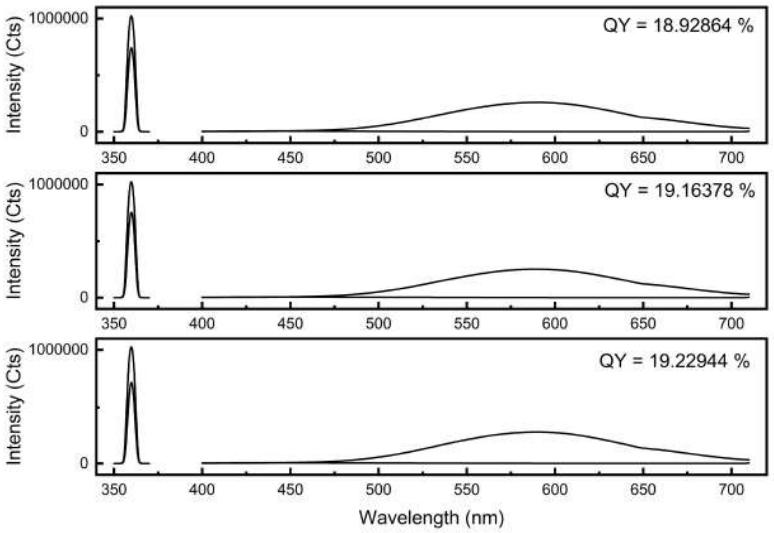

Figure S81. Photoluminescence and quantum yield of **1(2:1).**

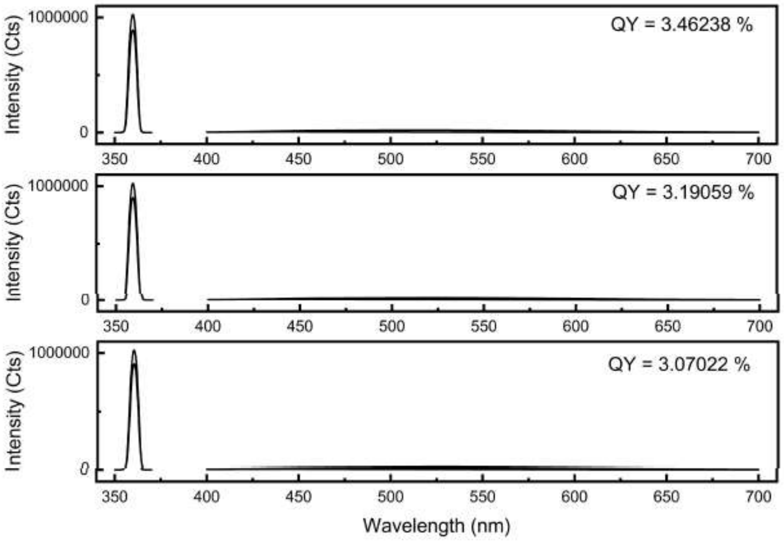

Figure S82. Photoluminescence and quantum yield of **2(2:1)**.

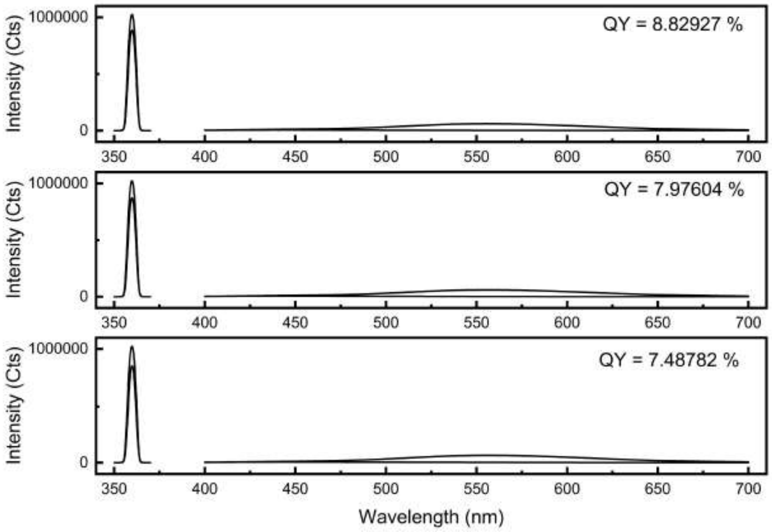

Figure S83. Photoluminescence and quantum yield of **3(2:1)**.

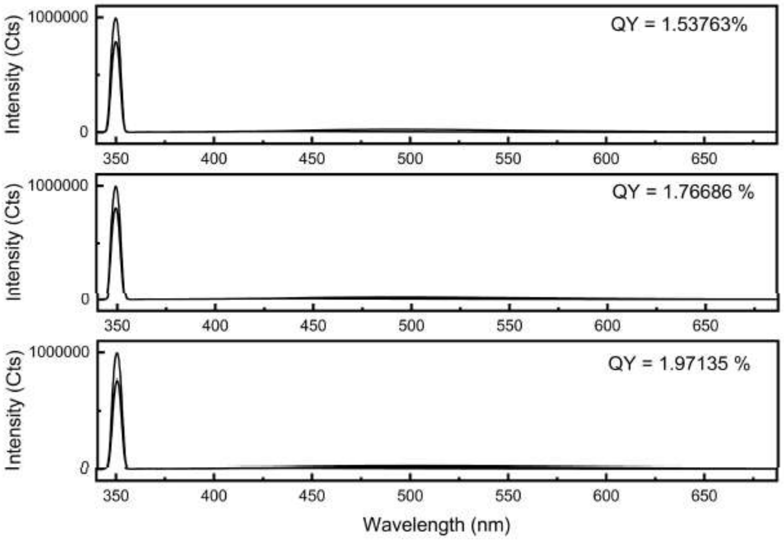

Figure S84. Photoluminescence and quantum yield of **4(2:1)**.

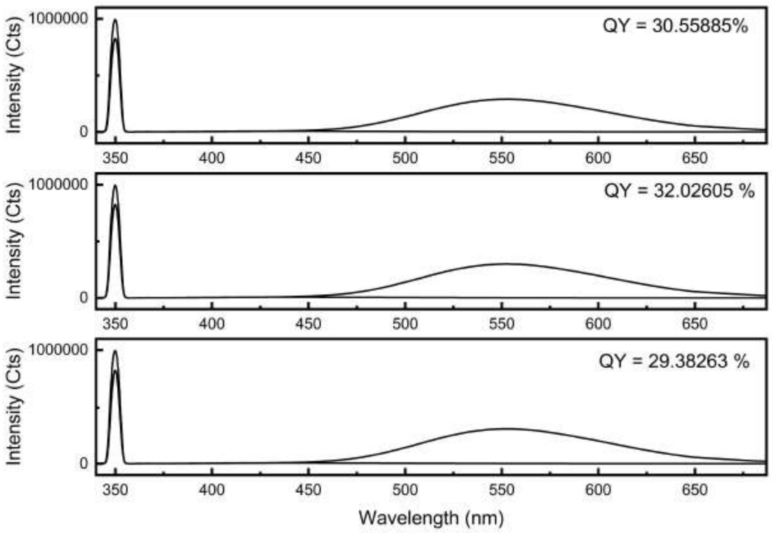

Figure S85. Photoluminescence and quantum yield of **5(2:1)**.

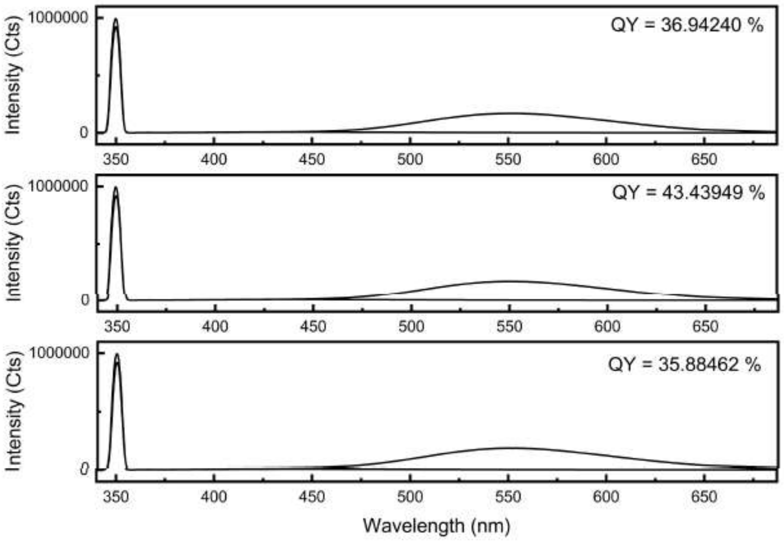

Figure S86. Photoluminescence and quantum yield of **6(2:1)**.

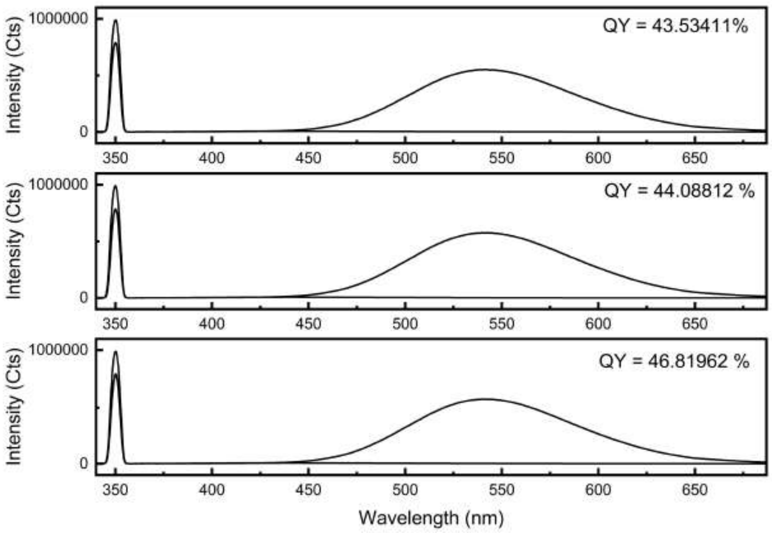

Figure S87. Photoluminescence and quantum yield of **7(2:1)**.

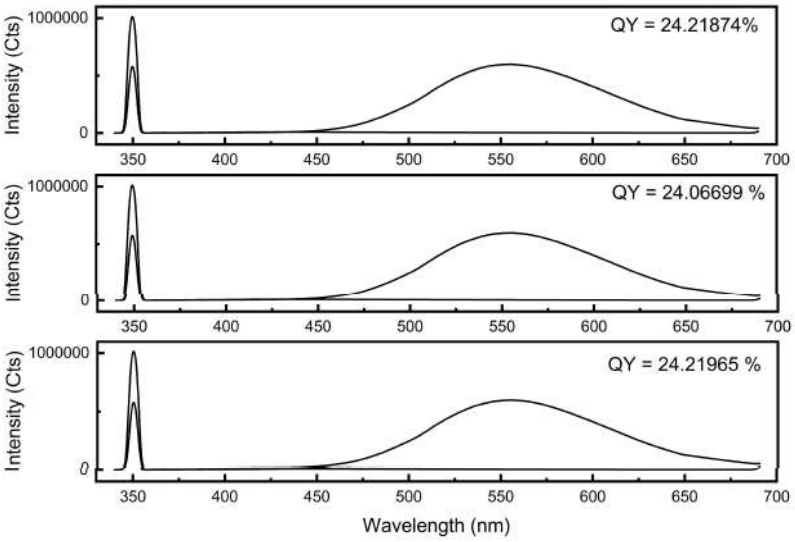

Figure S88. Photoluminescence and quantum yield of **8(2:1)**.

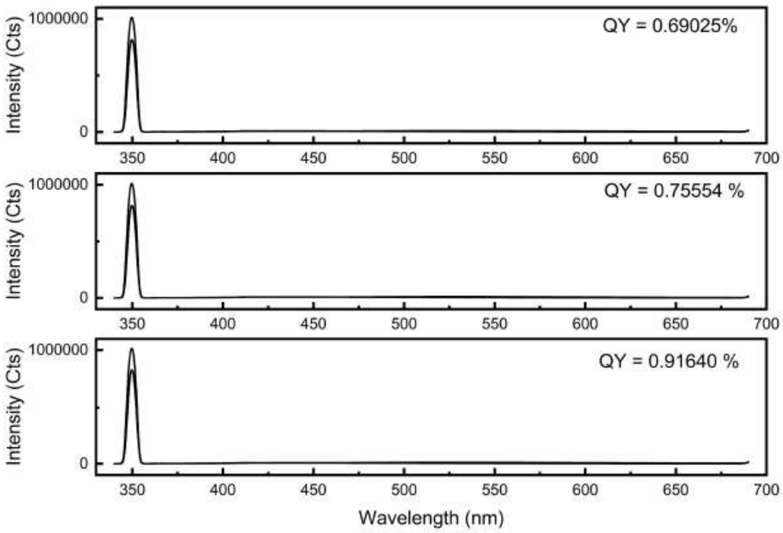

Figure S89. Photoluminescence and quantum yield of **1(3:2)**.

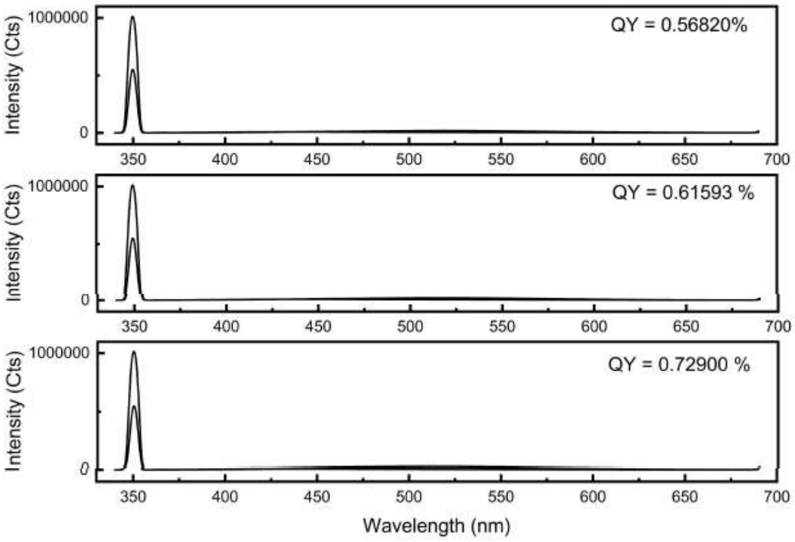

Figure S90. Photoluminescence and quantum yield of **2(3:2)**.

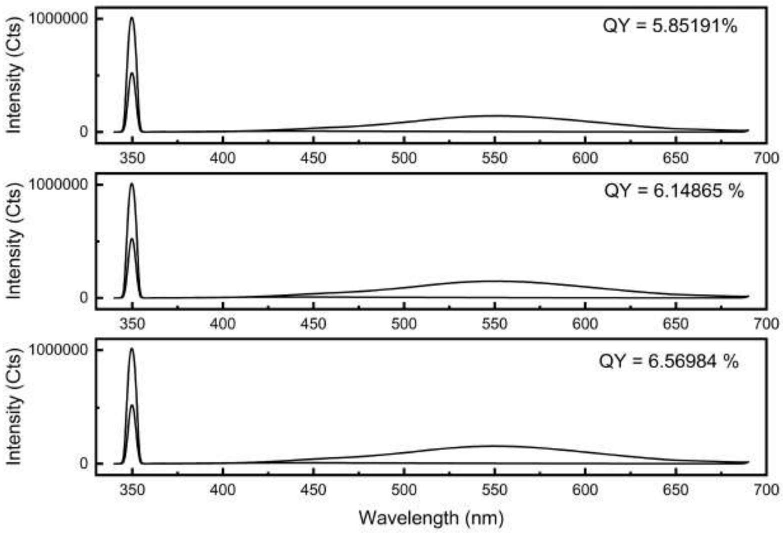

Figure S91. Photoluminescence and quantum yield of **3(3:2)**.

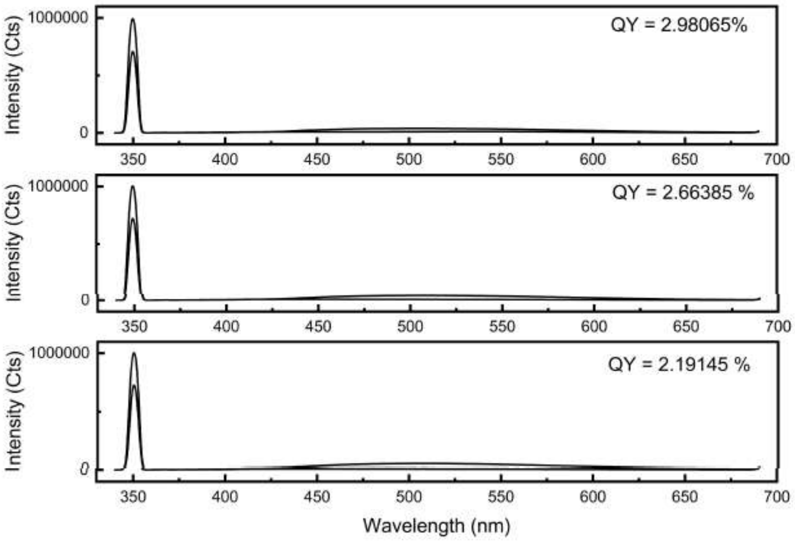

Figure S92. Photoluminescence and quantum yield of **4(3:2)**.

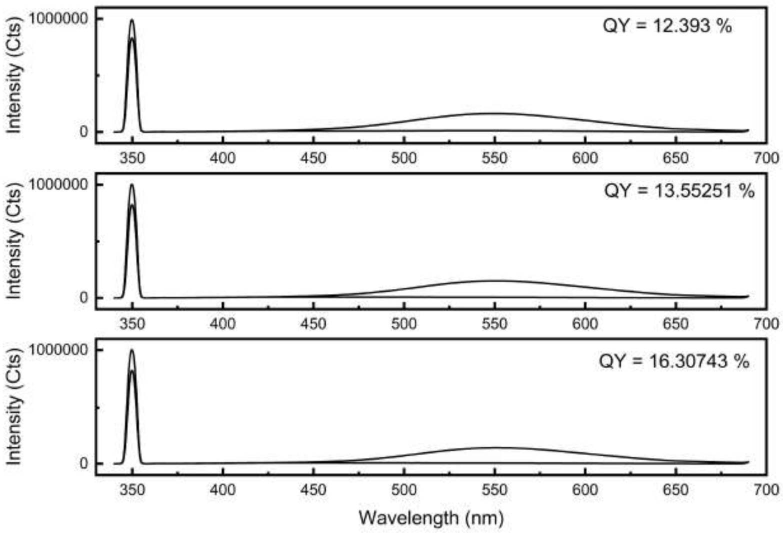

Figure S93. Photoluminescence and quantum yield of **5(3:2)**.

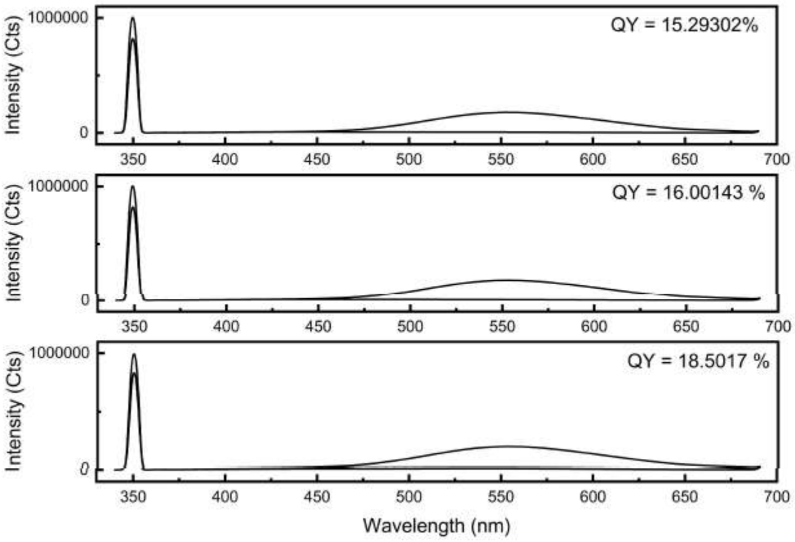

Figure S94. Photoluminescence and quantum yield of **6(3:2)**.

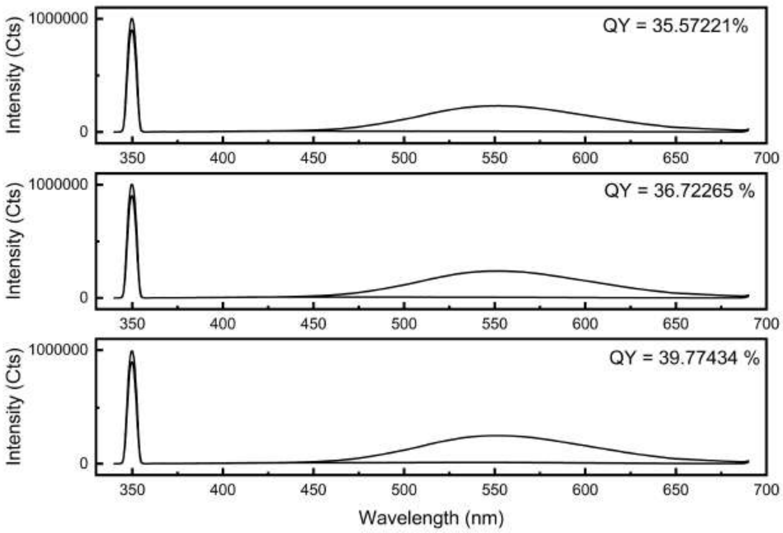

Figure S95. Photoluminescence and quantum yield of **7(3:2)**.

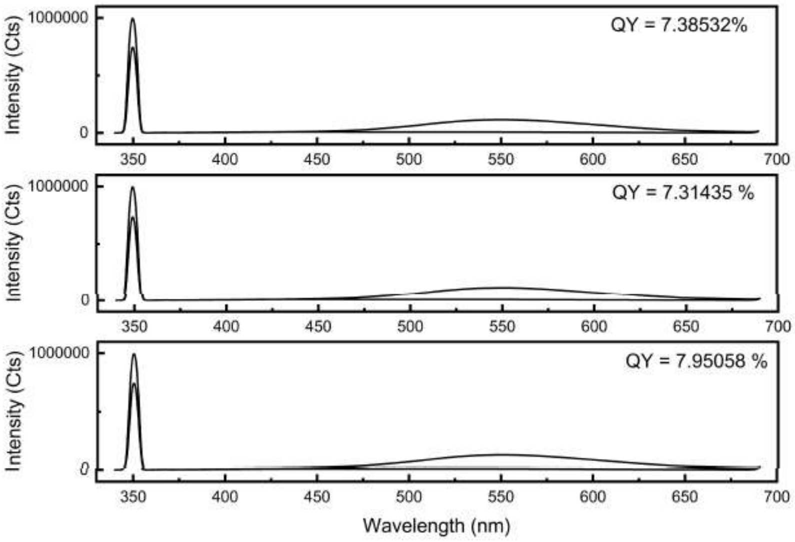

Figure S96. Photoluminescence and quantum yield of **8(3:2)**.

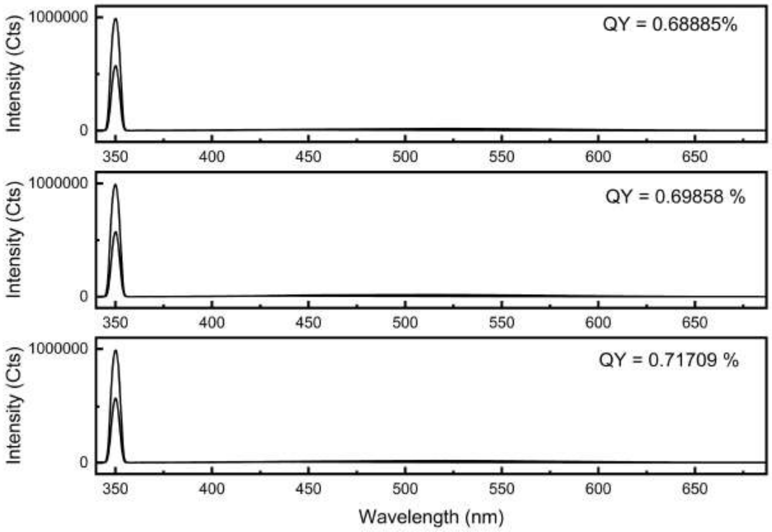

Figure S97. Photoluminescence and quantum yield of **2(1:1)**.

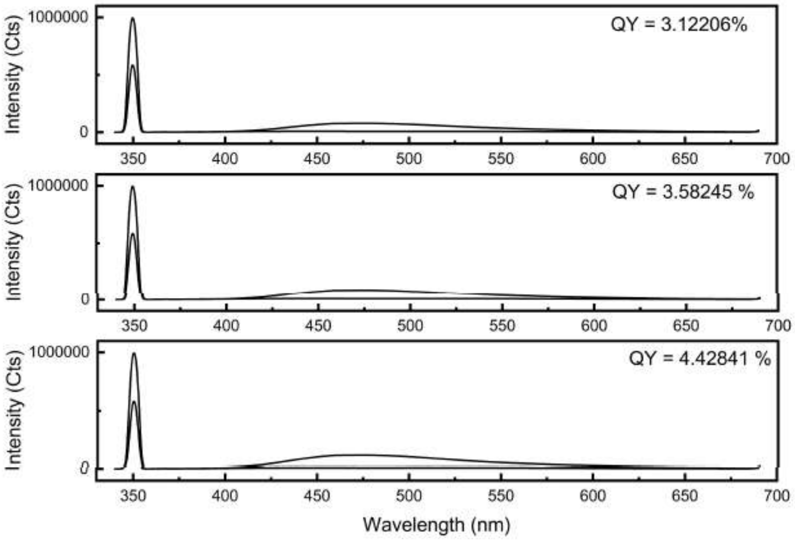

Figure S98. Photoluminescence and quantum yield of **3(1:1)**.

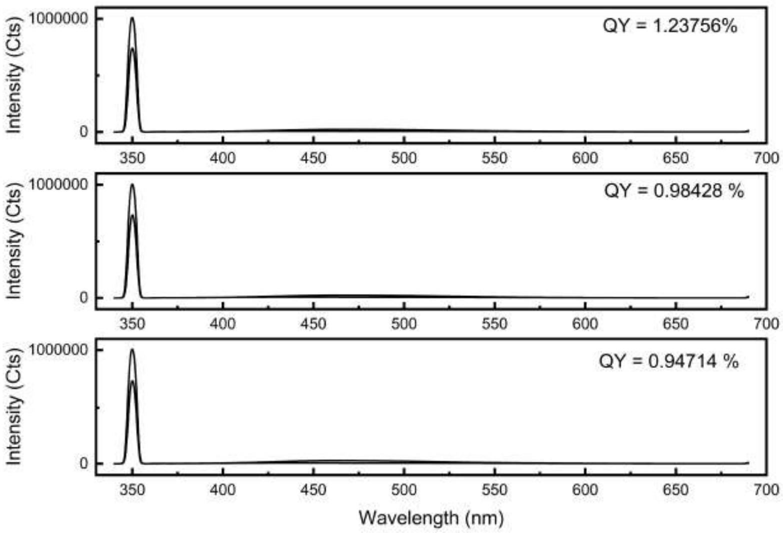

Figure S99. Photoluminescence and quantum yield of **5(1:1)**.

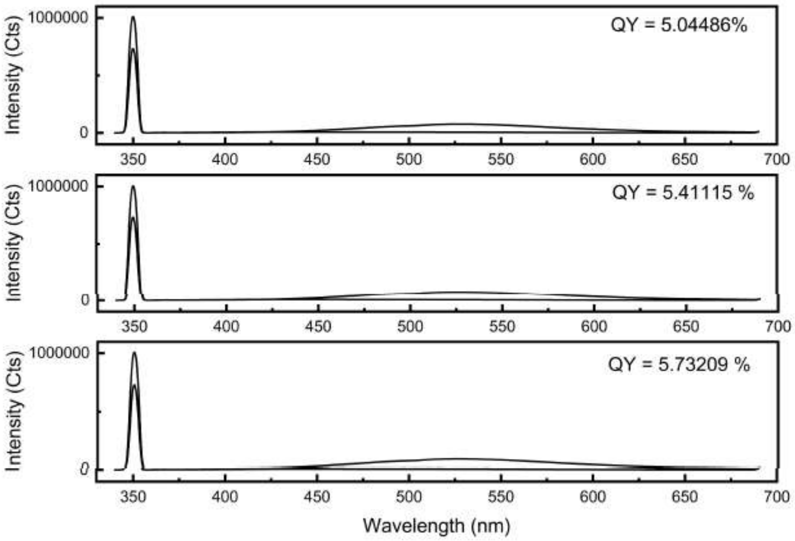

Figure S100. Photoluminescence and quantum yield of **7(1:1)**.

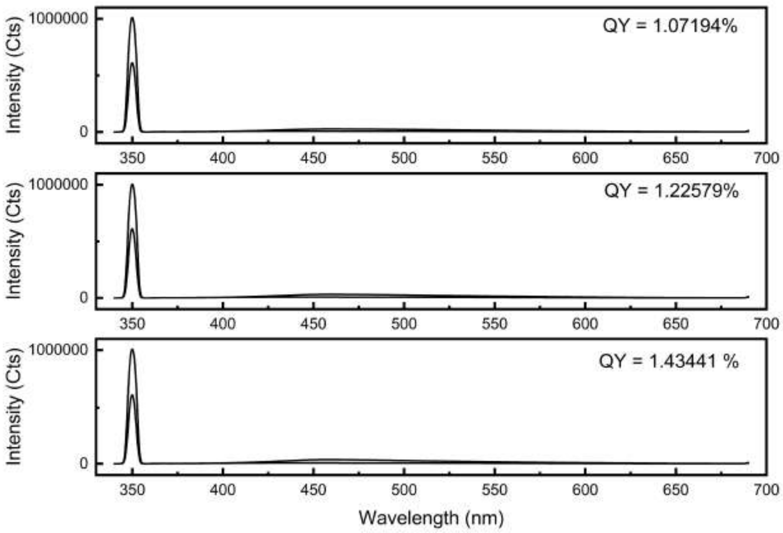

Figure S101. Photoluminescence and quantum yield of **8(1:1)**.

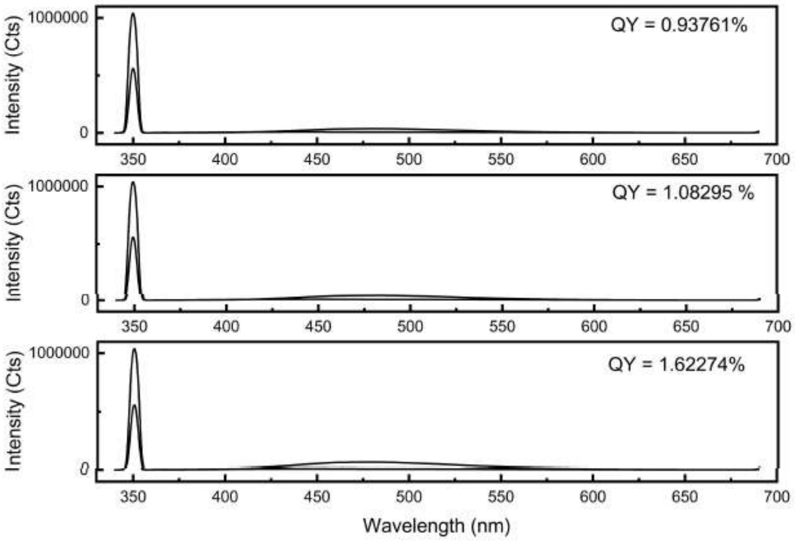

Figure S102. Photoluminescence and quantum yield of **3(2:3)**.

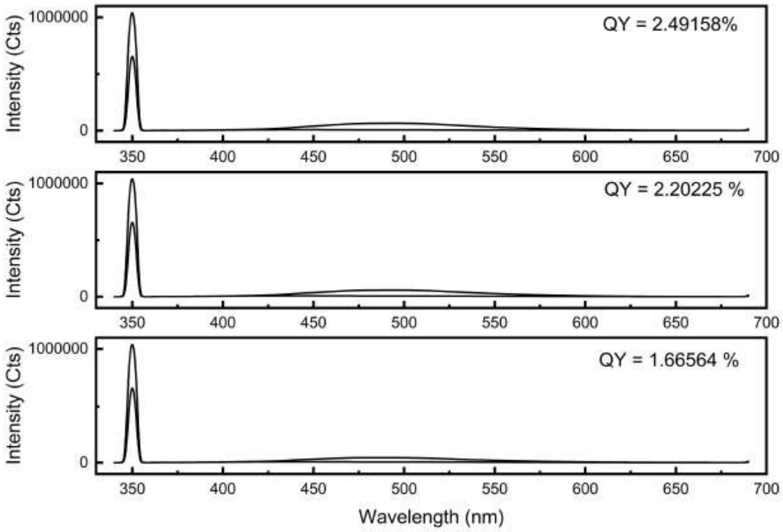

Figure S103. Photoluminescence and quantum yield of **4(2:3)**.

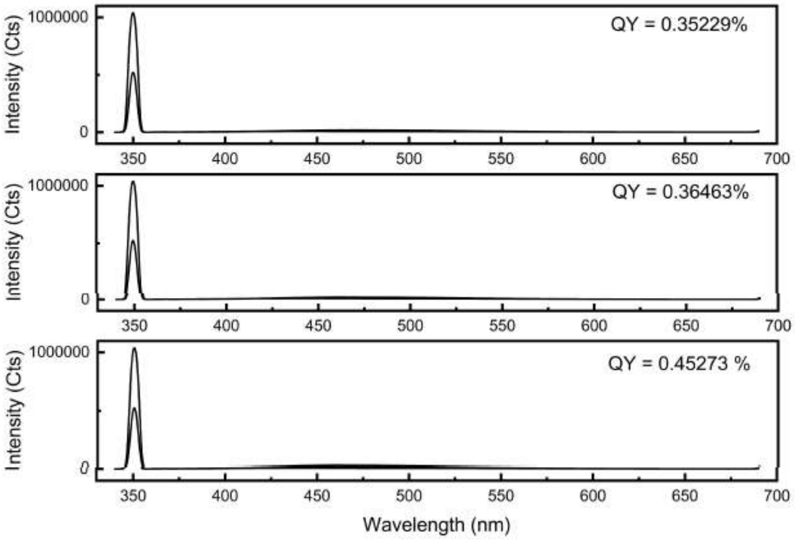

Figure S104. Photoluminescence and quantum yield of **5(2:3)**.

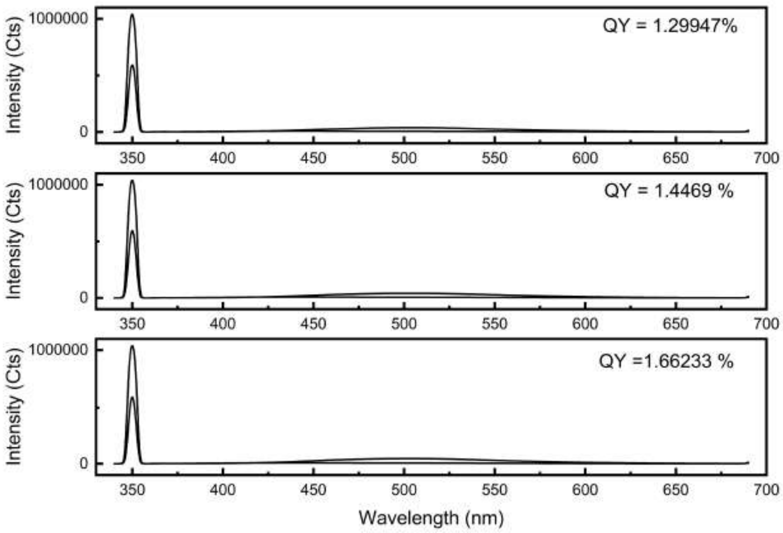

Figure S105. Photoluminescence and quantum yield of **7(2:3)**.

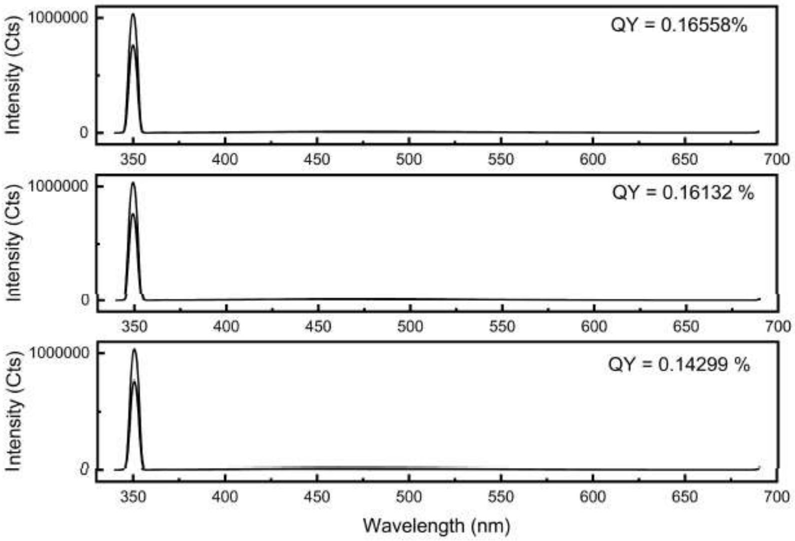

Figure S106. Photoluminescence and quantum yield of **8(2:3)**.

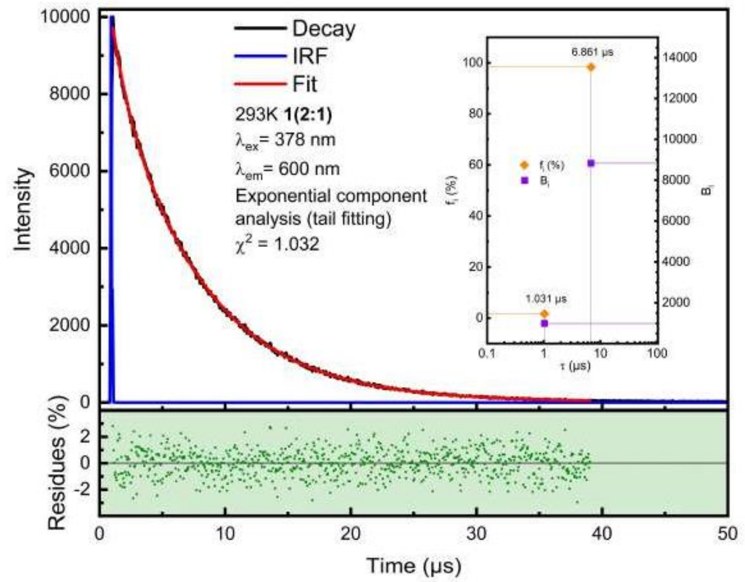

Figure S107. PL decay of **1(2:1)** at 293K.

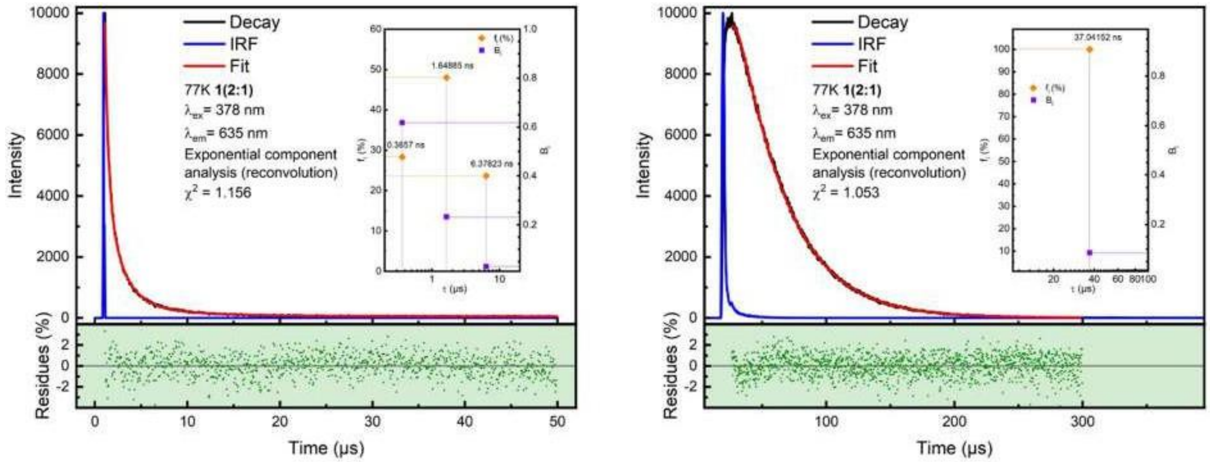

Figure S108. PL decay of **1(2:1)** at 77K. Left : short lifetimes. Right : long lifetimes.

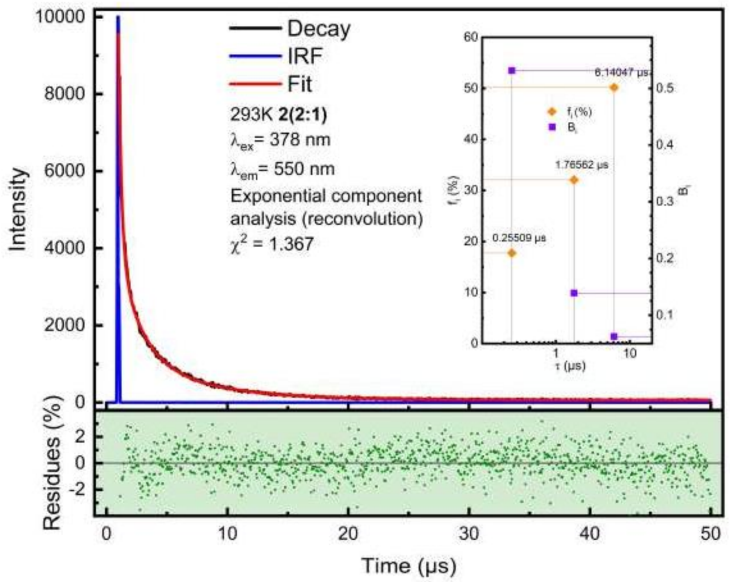

Figure S109. PL decay of **2(2:1)** at 293K.

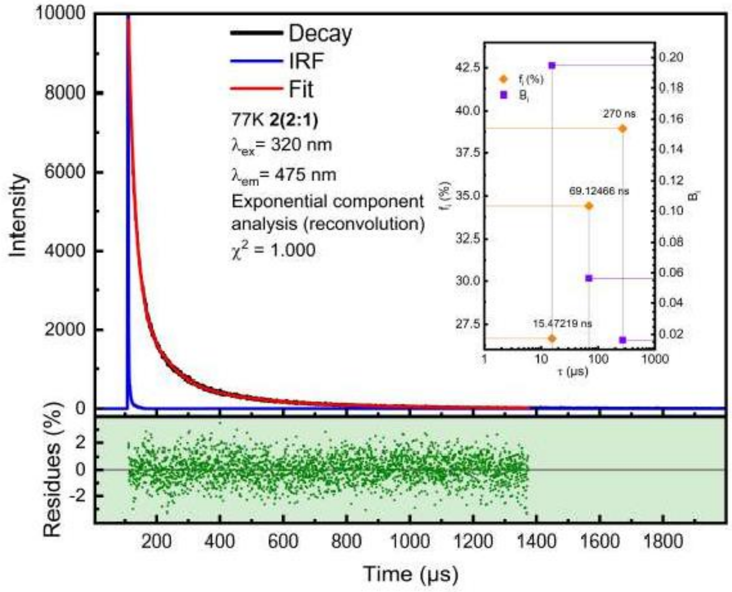

Figure S110. PL decay of **2(2:1)** at 77K.

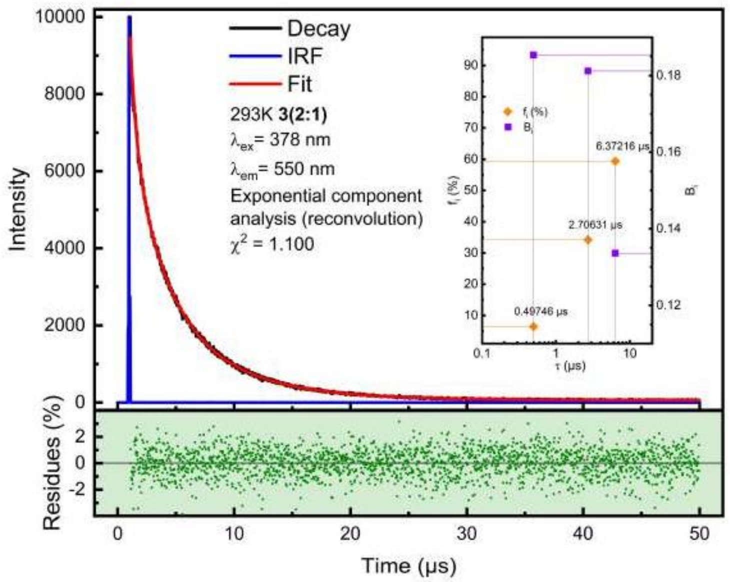

Figure S111. PL decay of **3(2:1)** at 293K.

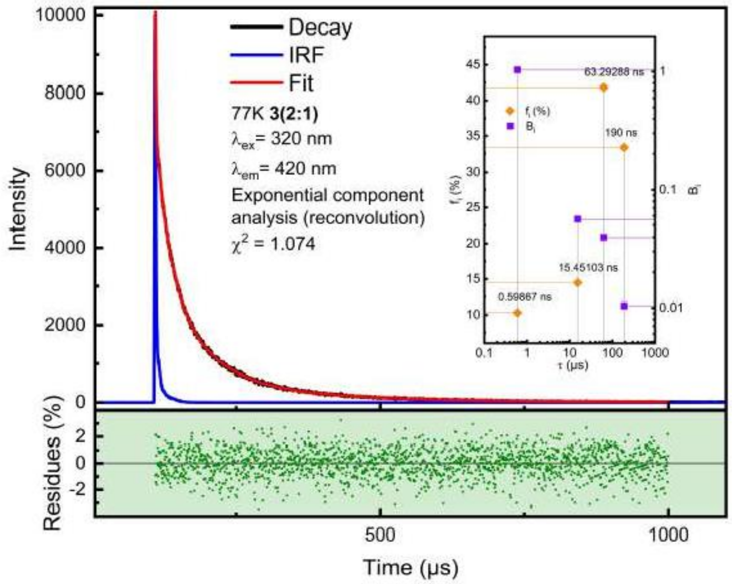

Figure S112. PL decay of **3(2:1)** at 77K.

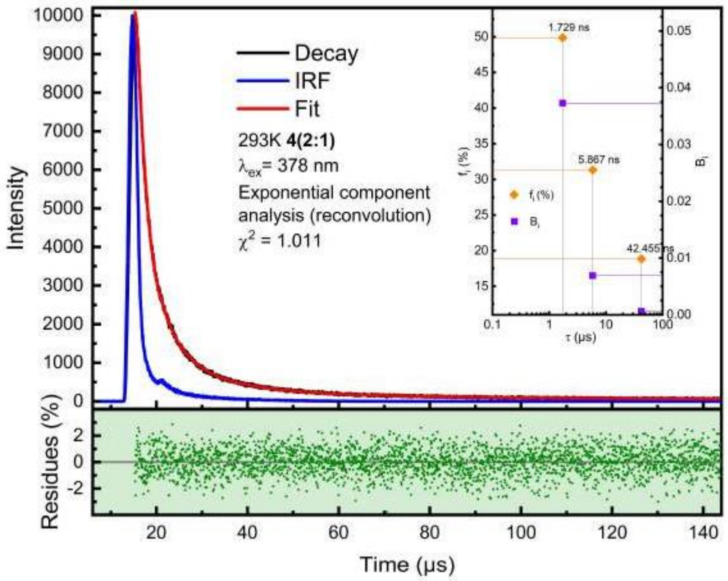

Figure S113. PL decay of **4(2:1)** at 293K.

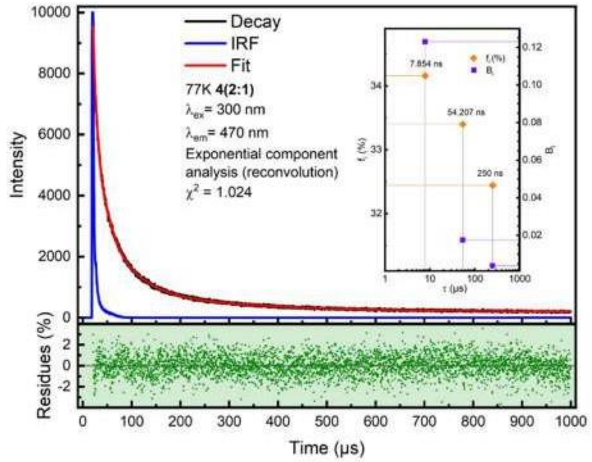

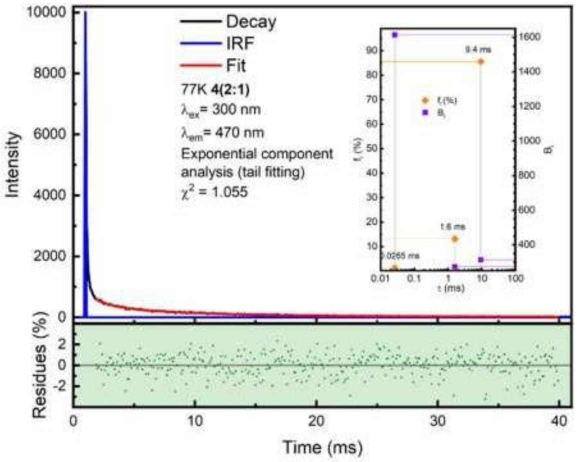

Figure S114. PL decay of **4(2:1)** at 77K. Left : short lifetimes. Right : long lifetimes.

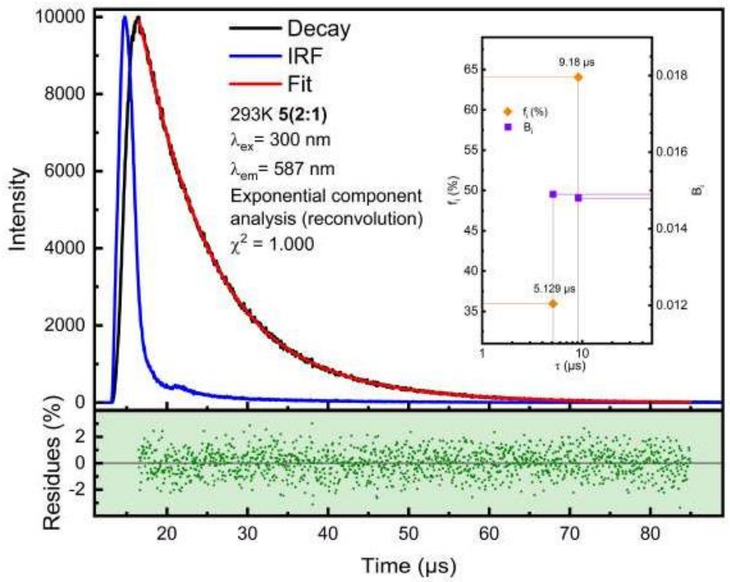

Figure S115. PL decay of **5(2:1)** at 293K.

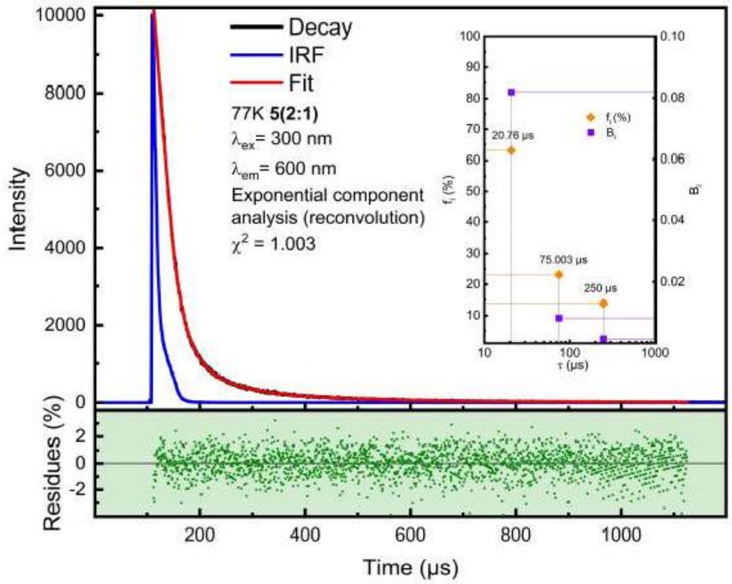

Figure S116. PL decay of **5(2:1)** at 77K.

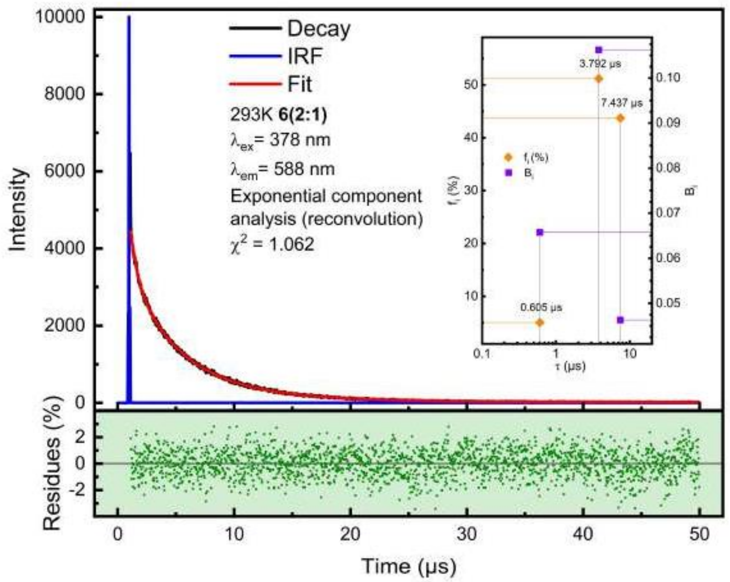

Figure S117. PL decay of **6(2:1)** at 293K.

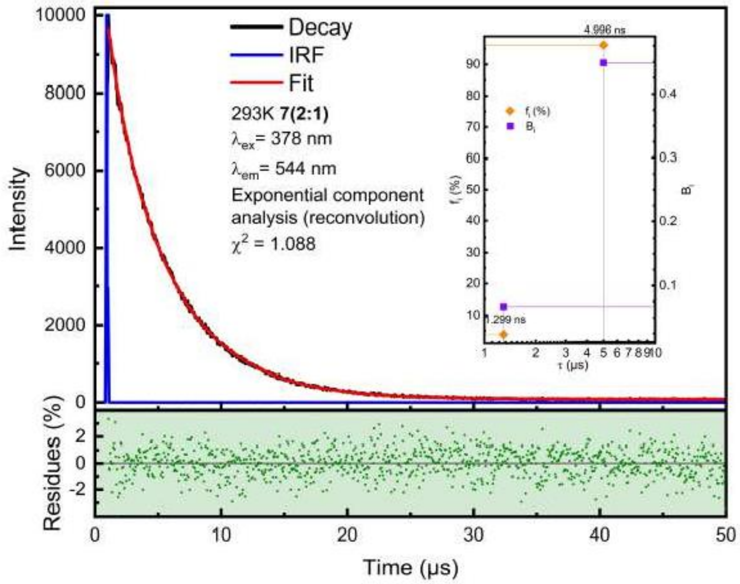

Figure S118. PL decay of **7(2:1)** at 293K.

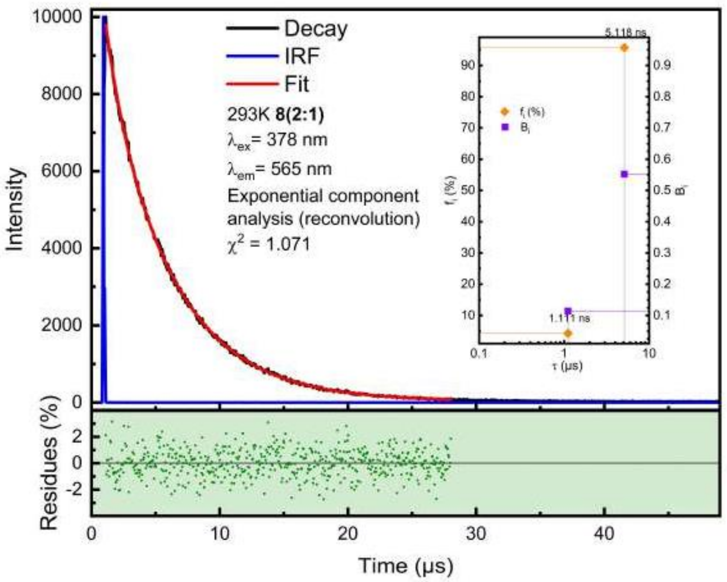

Figure S119. PL decay of **8(2:1)** at 293K.

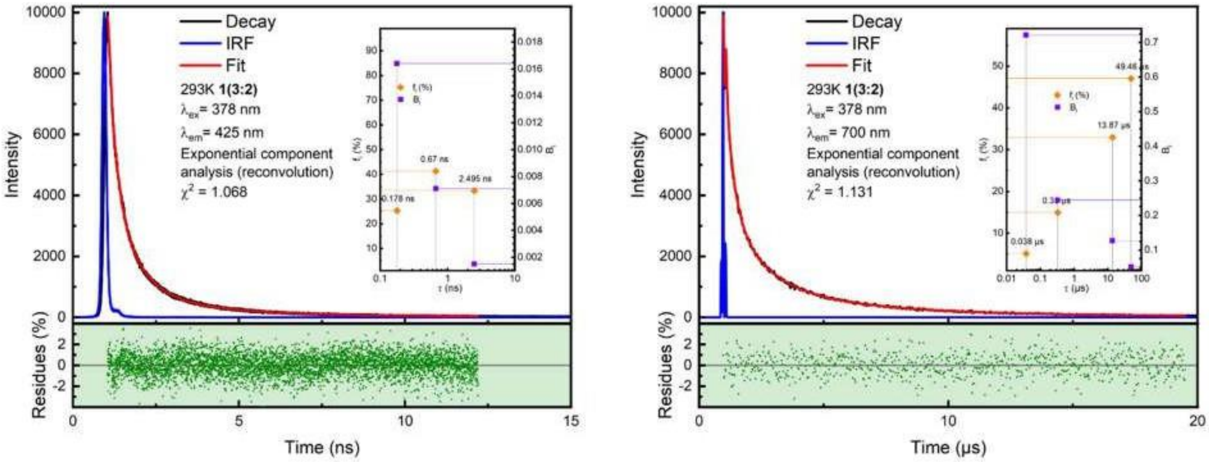

Figure S120. PL decay of **1(3:2)** at 293K. Left : short lifetimes. Right : long lifetimes.

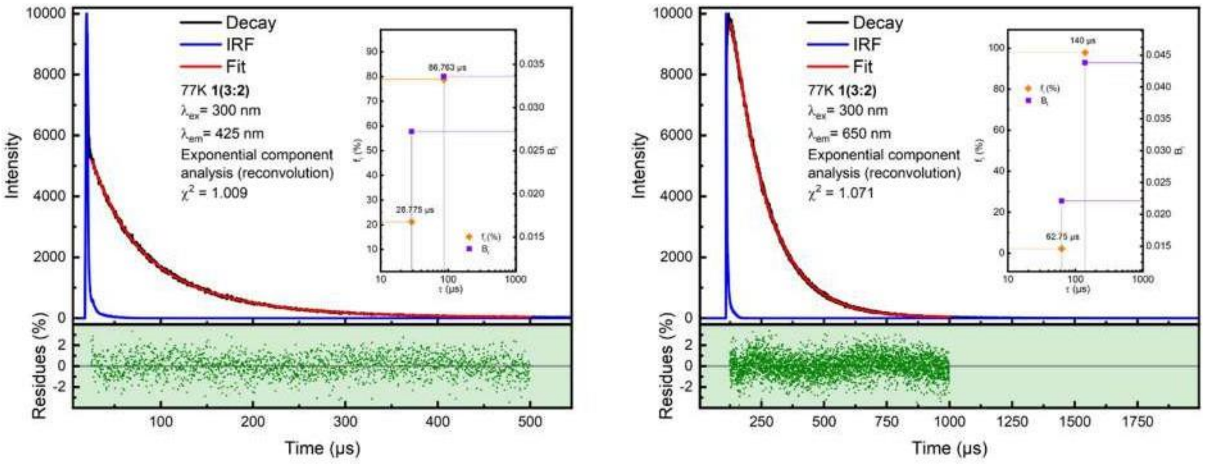

Figure S121. PL decay of **1(3:2)** at 77K. Left : short lifetimes. Right : long lifetimes.

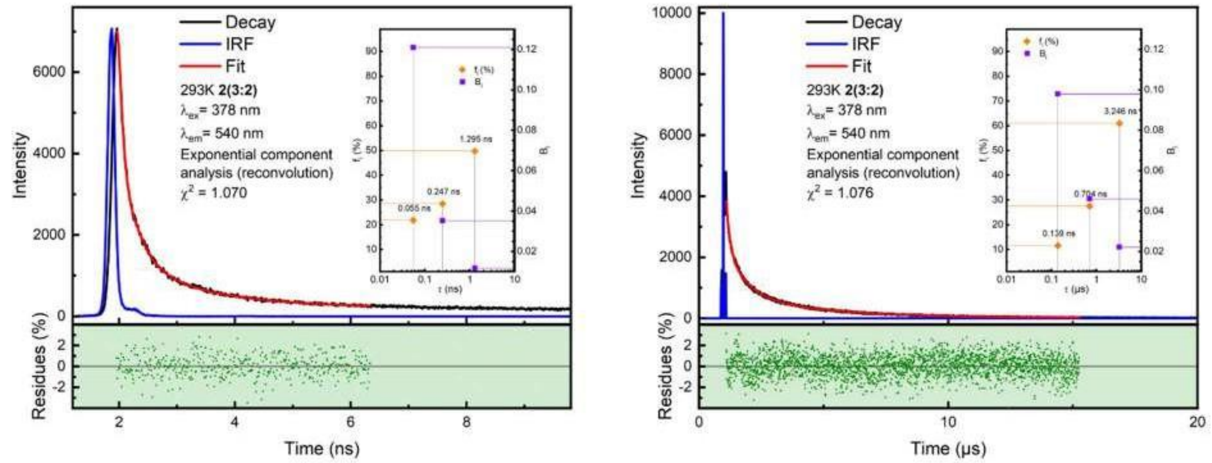

Figure S122. PL decay of **2(3:2)** at 293K. Left : short lifetimes. Right : long lifetimes.

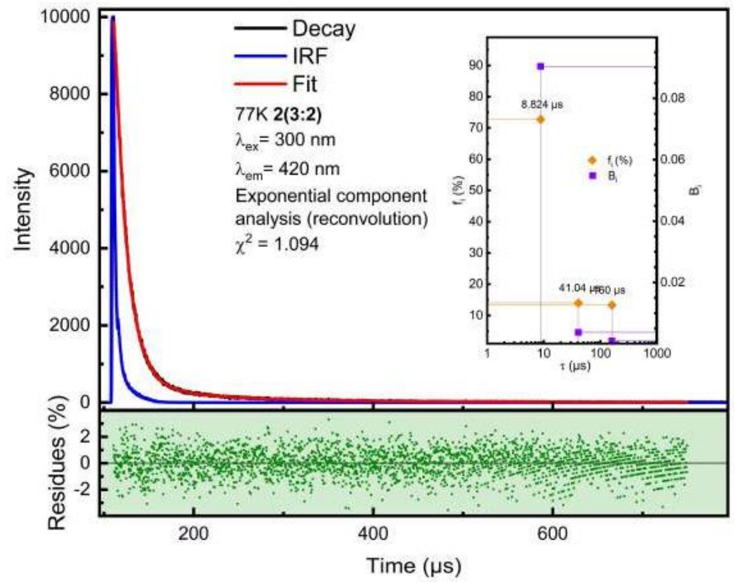

Figure S123. PL decay of **2(3:2)** at 77K.

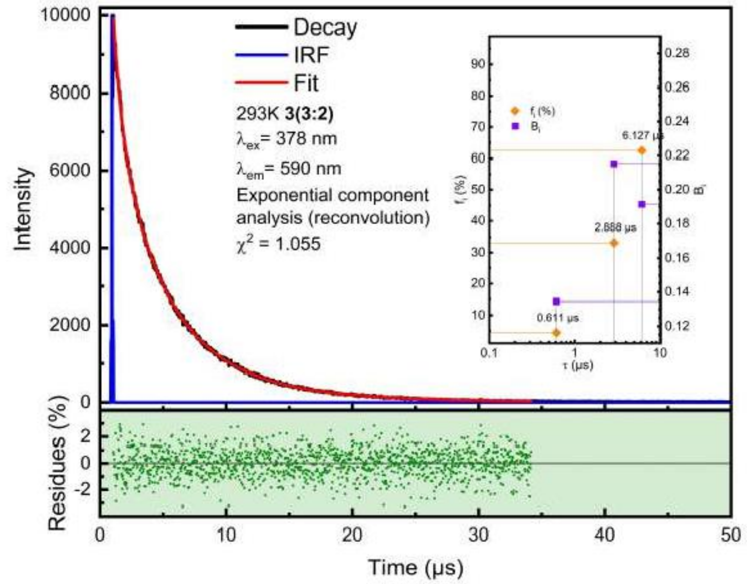

Figure S124. PL decay of **3(3:2)** at 293K.

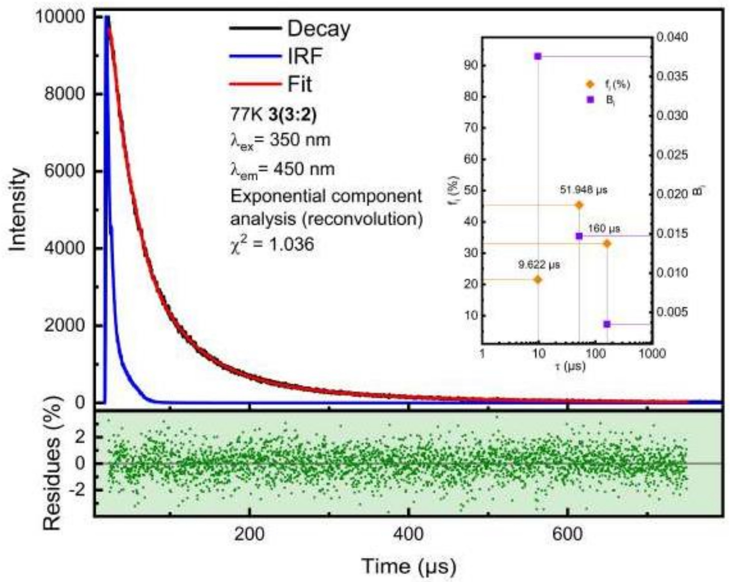

Figure S125. PL decay of **3(3:2)** at 77K.

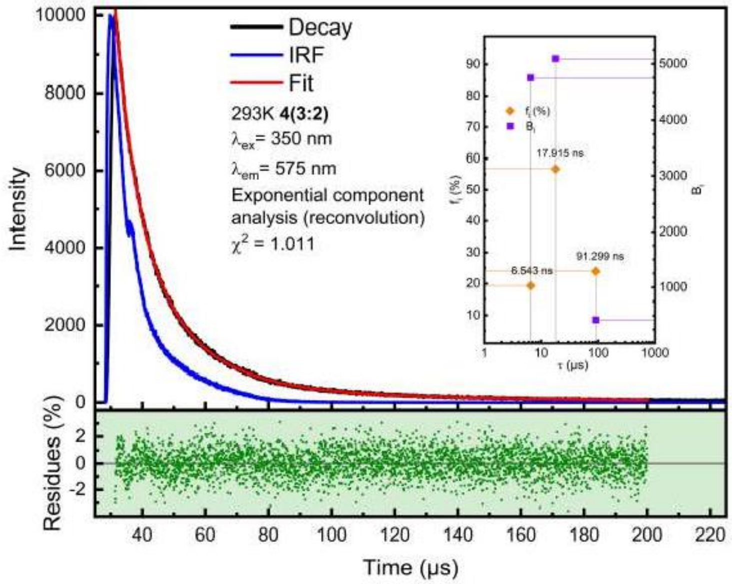

Figure S126. PL decay of **4(3:2)** at 293K.

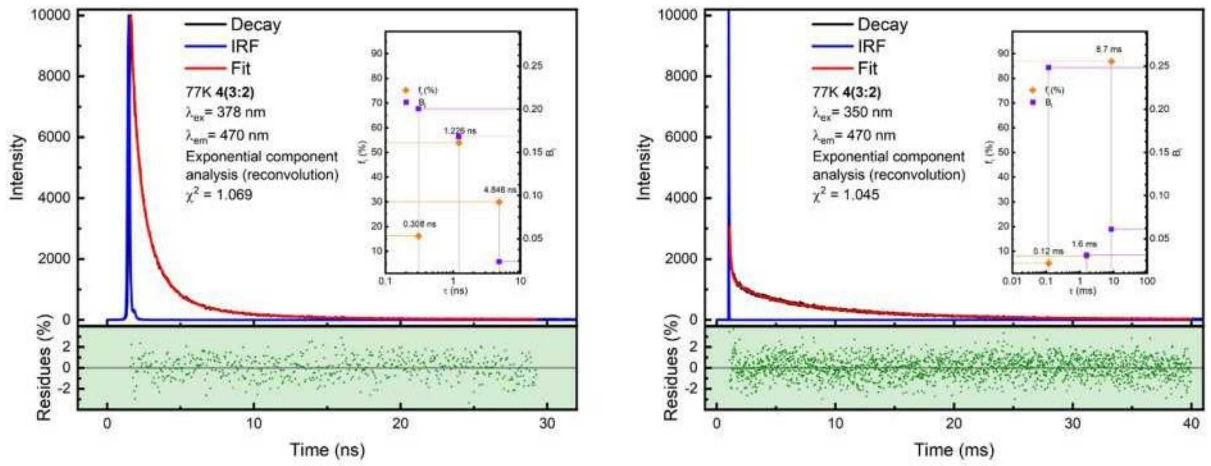

Figure S127. PL decay of **4(3:2)** at 77K. Left : short lifetimes. Right : long lifetimes.

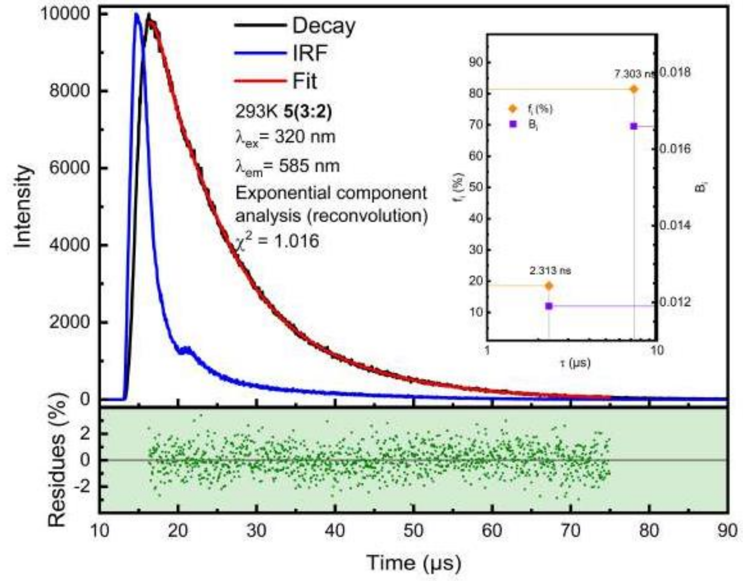

Figure S128. PL decay of **5(3:2)** at 293K.

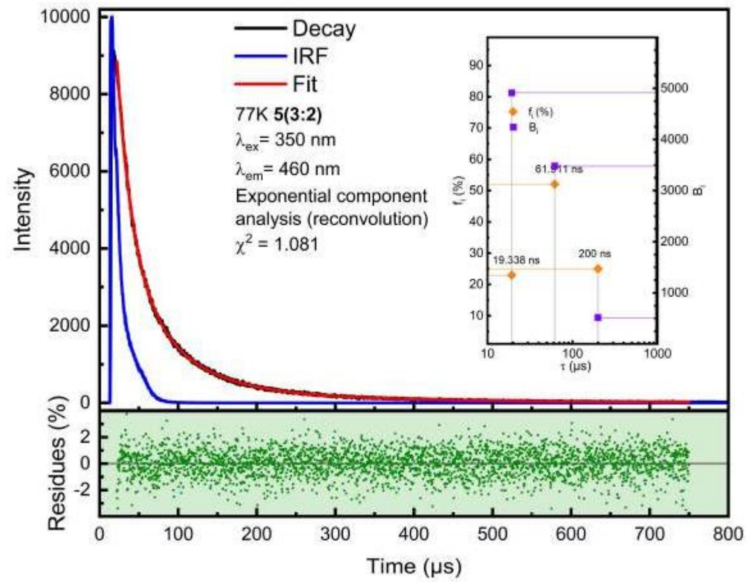

Figure S129. PL decay of **5(3:2)** at 77K.

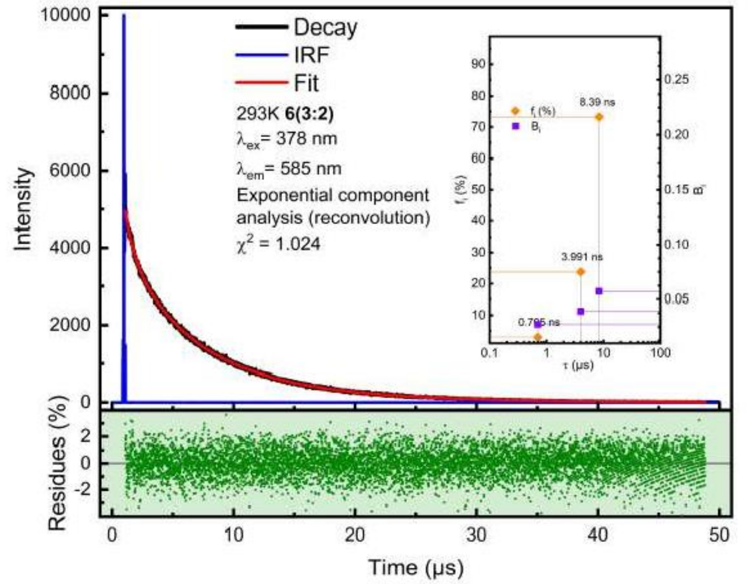

Figure S130. PL decay of **6(3:2)** at 293K.

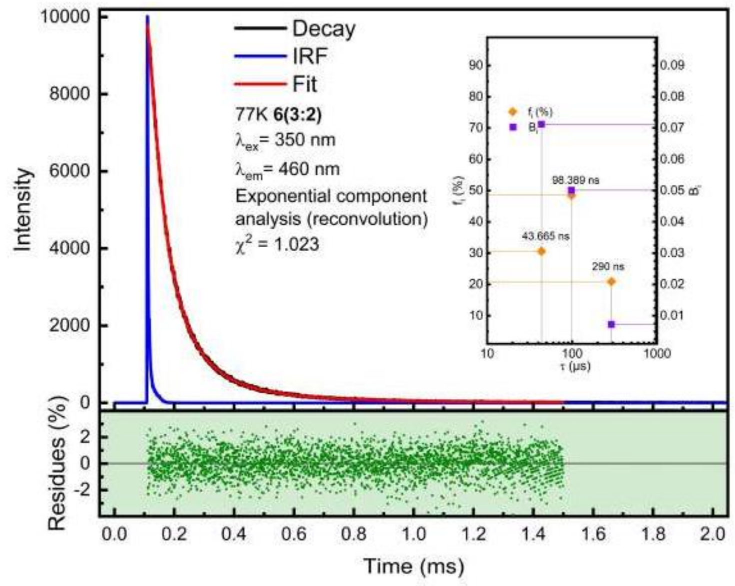

Figure S131. PL decay of **6(3:2)** at 77K.

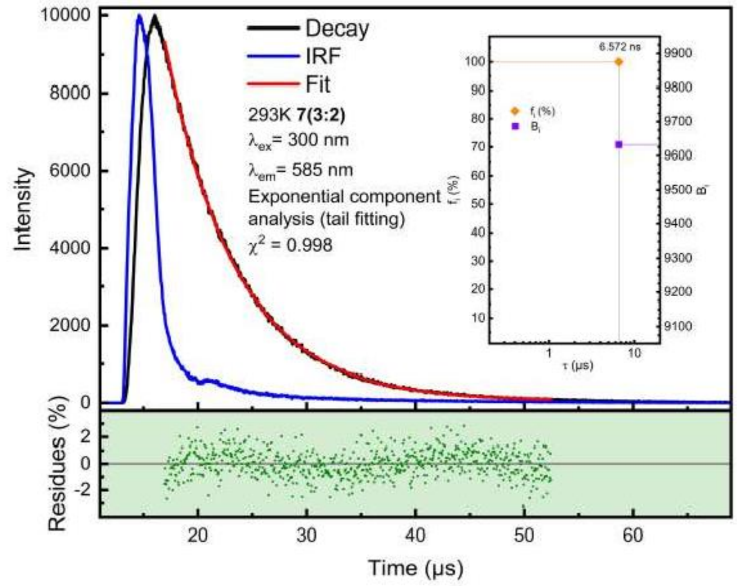

Figure S132. PL decay of **7(3:2)** at 293K.

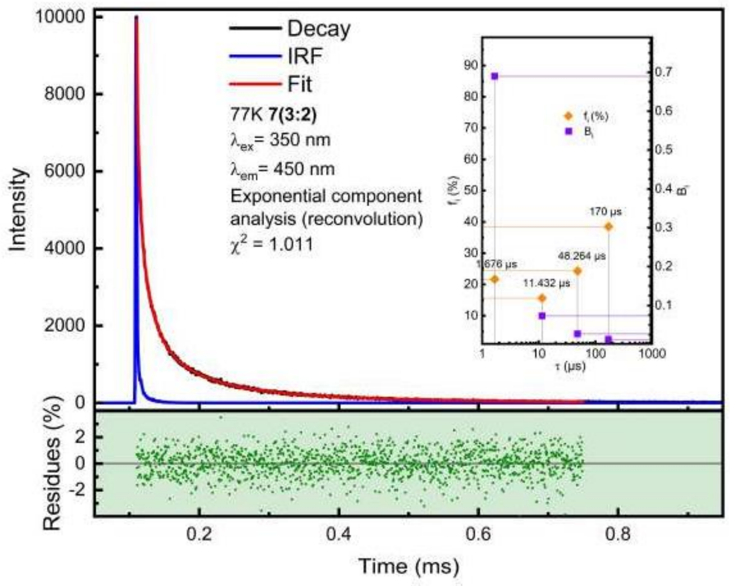

Figure S133. PL decay of **7(3:2)** at 77K.

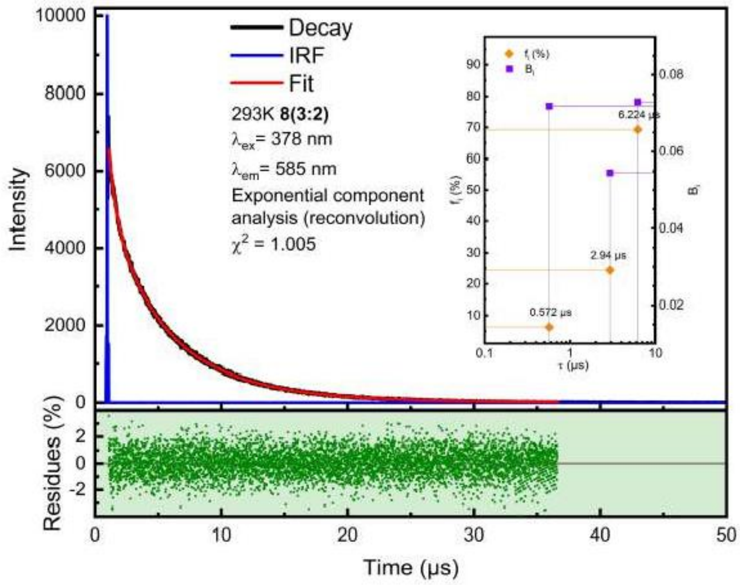

Figure S134. PL decay of **8(3:2)** at 293K.

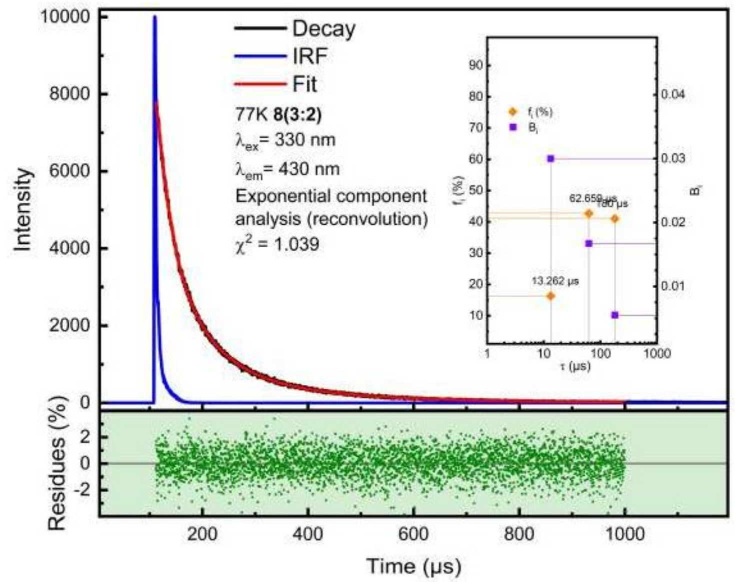

Figure S135. PL decay of **8(3:2)** at 77K.

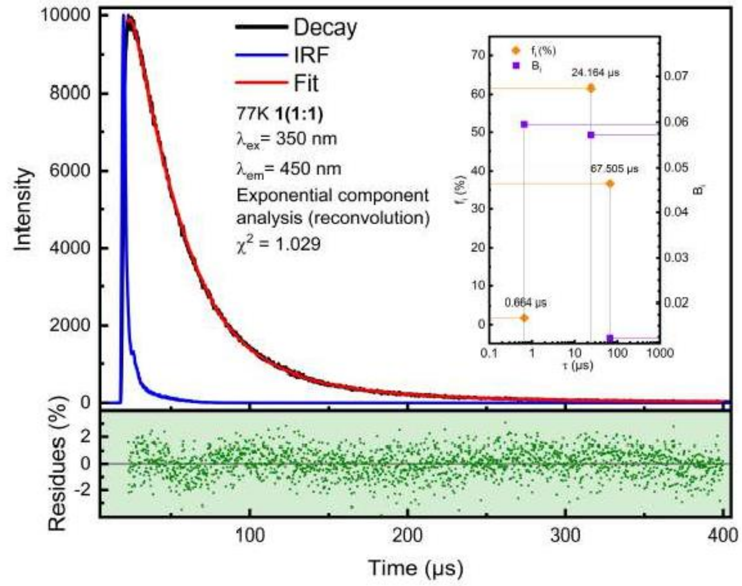

Figure S136. PL decay of **1(1:1)** at 77K.

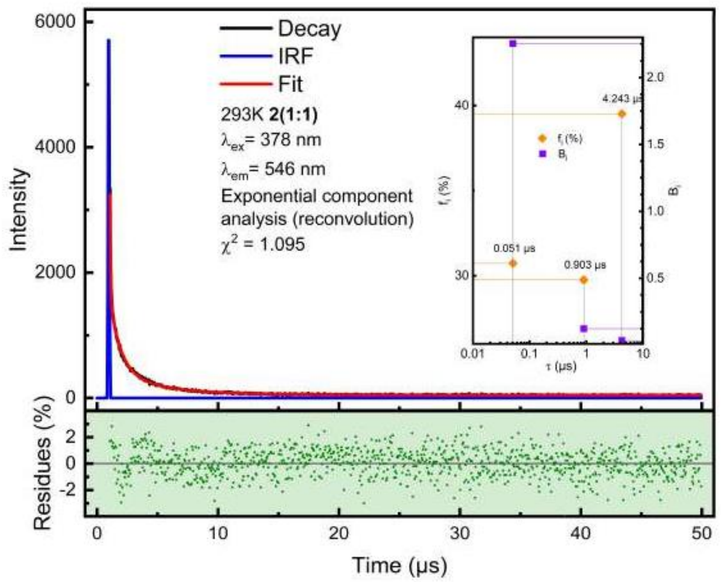

Figure S137. PL decay of **2(1:1)** at 293K.

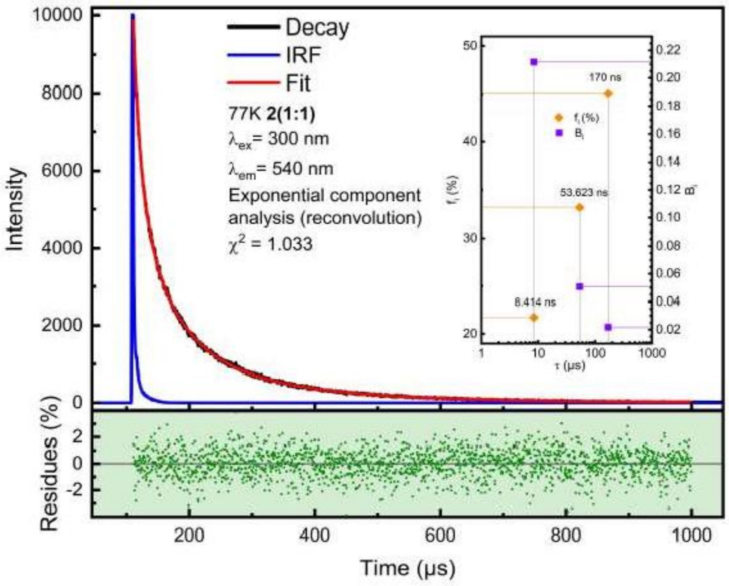

Figure S138. PL decay of **2(1:1)** at 77K.

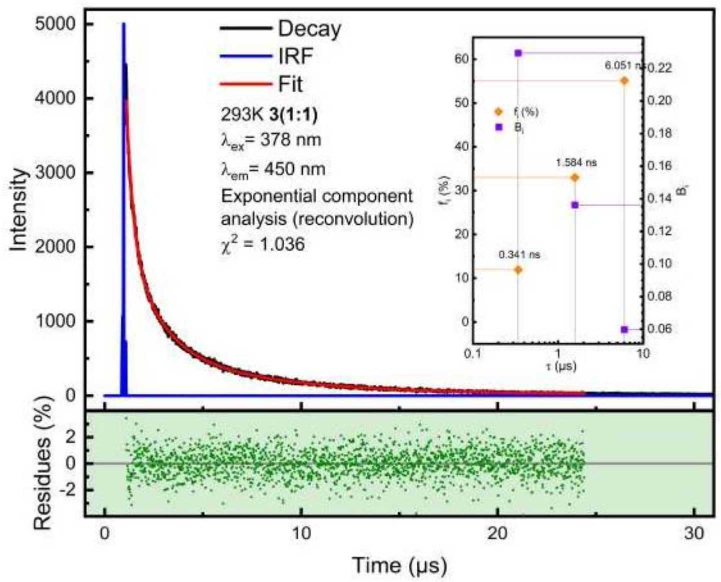

Figure S139. PL decay of **3(1:1)** at 293K.

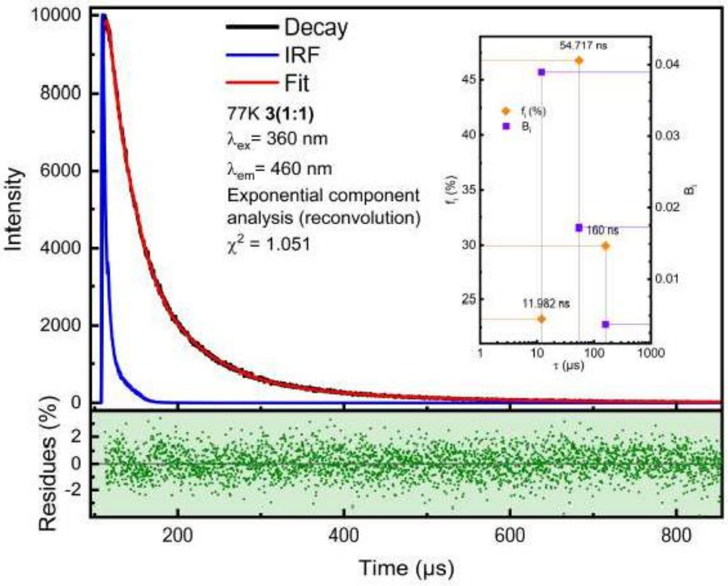

Figure S140. PL decay of **3(1:1)** at 77K.

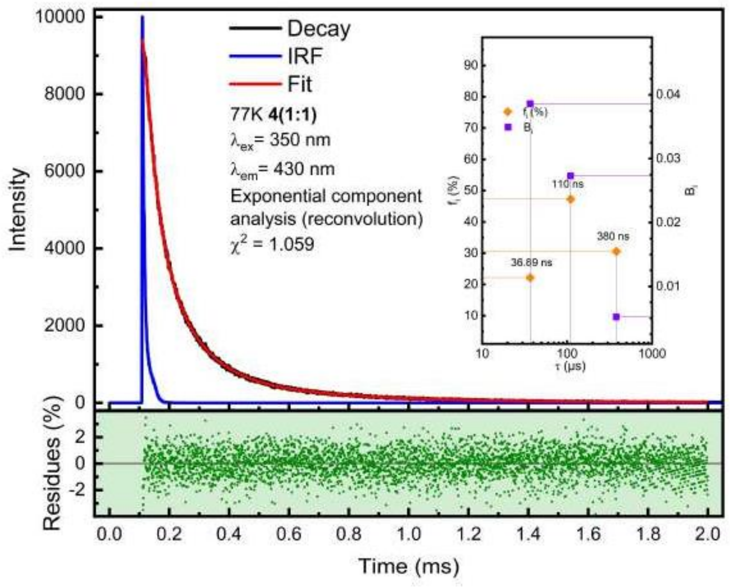

Figure S141. PL decay of **4(1:1)** at 77K.

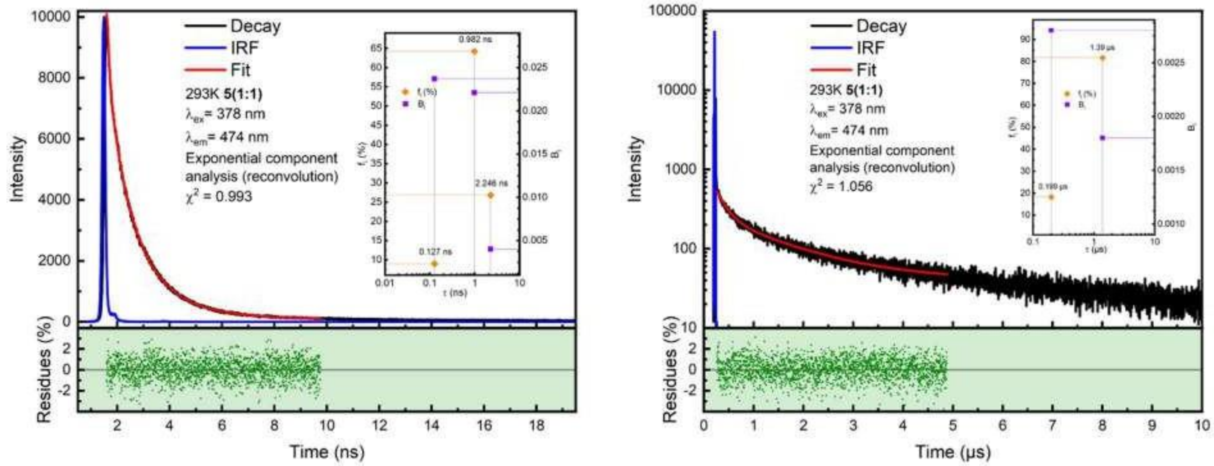

Figure S142. PL decay of **5(1:1)** at 293K. Left : short lifetimes. Right : long lifetimes.

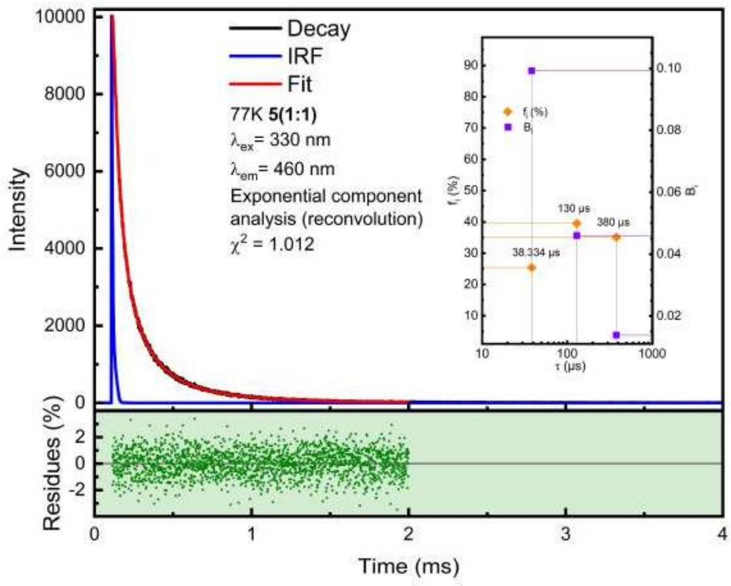

Figure S143. PL decay of **5(1:1)** at 77K.

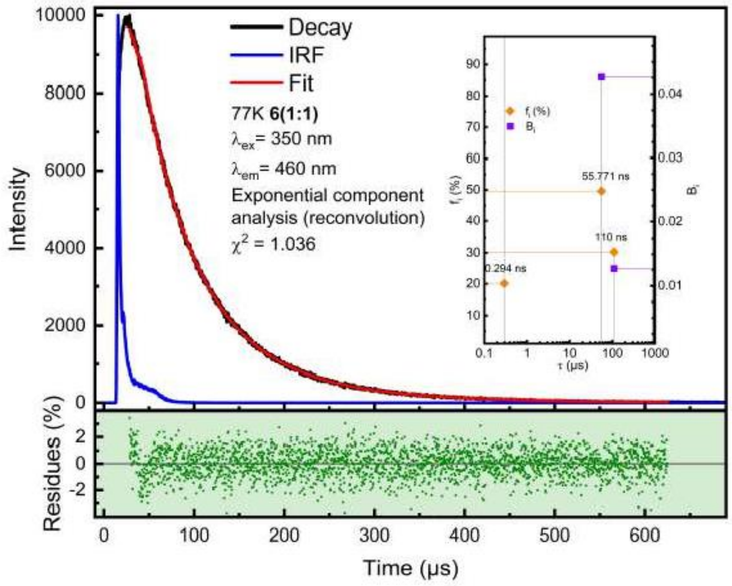

Figure S144. PL decay of **6(1:1)** at 77K.

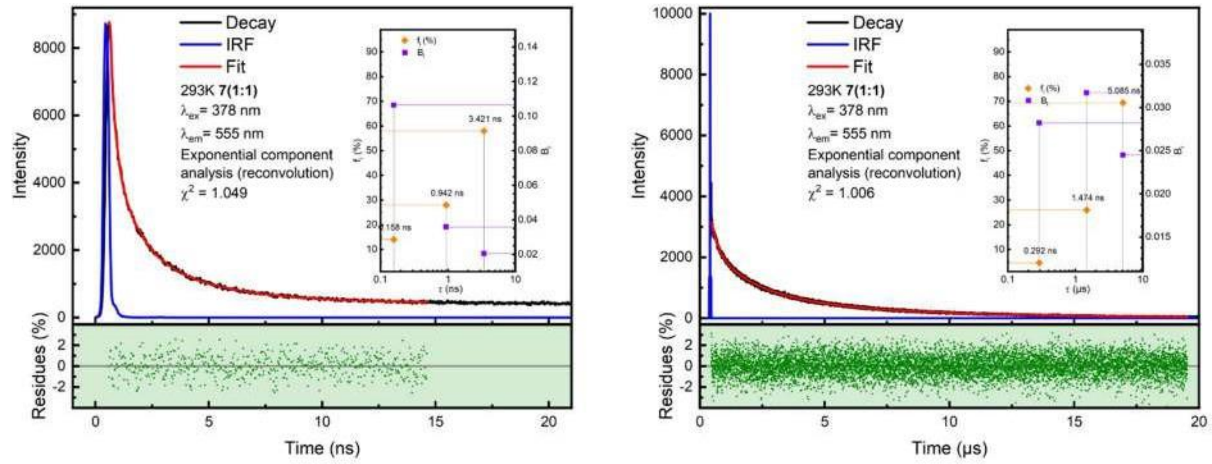

Figure S145. PL decay of **7(1:1)** at 293K. Left : short lifetimes. Right : long lifetimes.

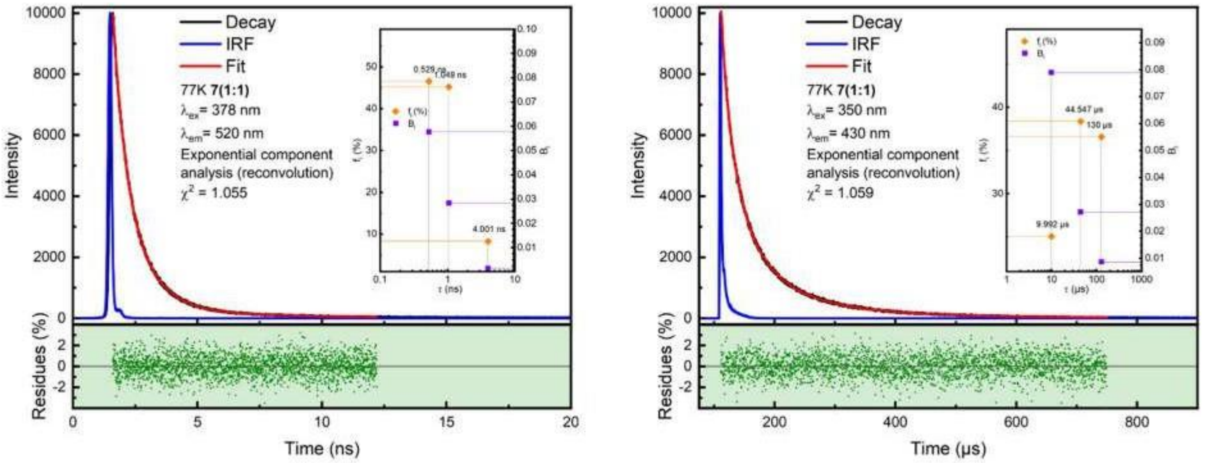

Figure S146. PL decay of **7(1:1)** at 77K. Left : short lifetimes. Right : long lifetimes.

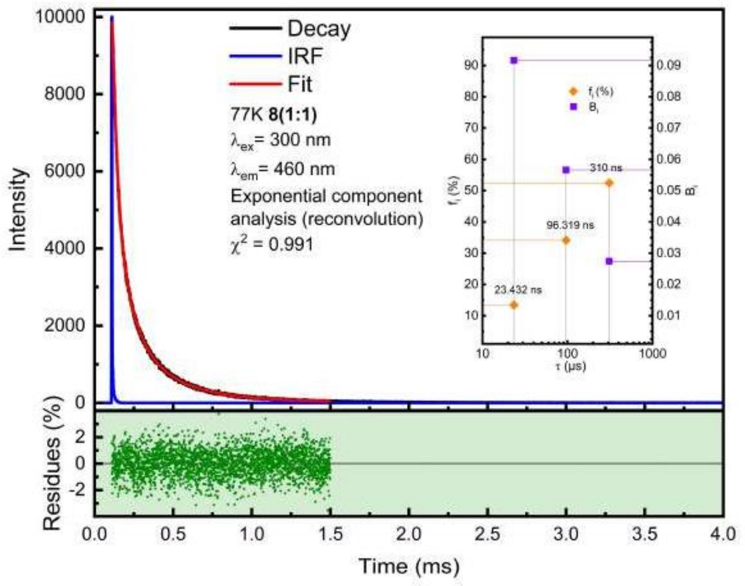

Figure S147. PL decay of **8(1:1)** at 77K.

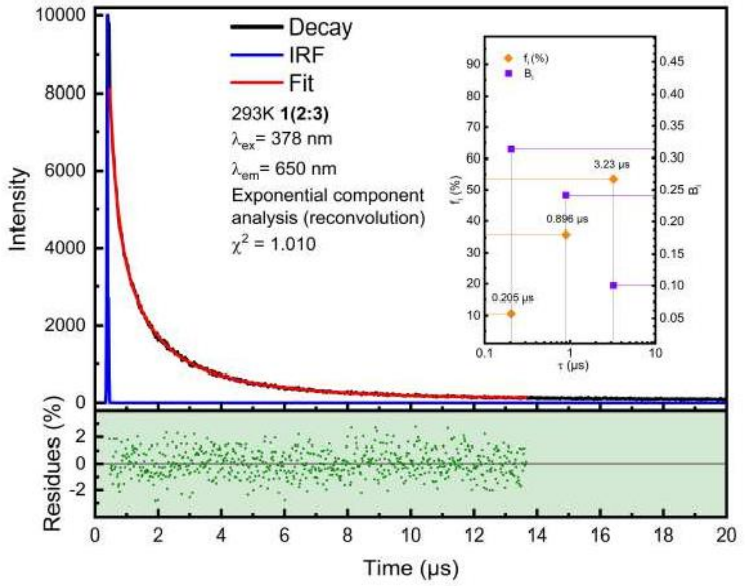

Figure S148. PL decay of **1(2:3)** at 293K.

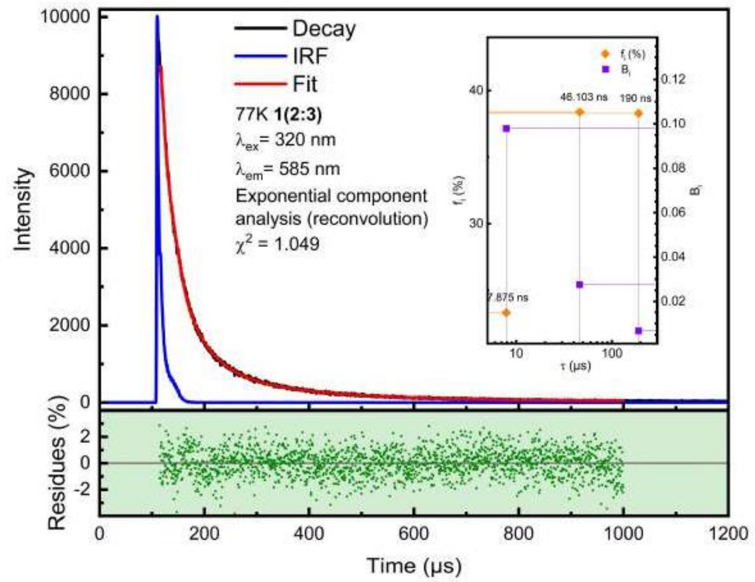

Figure S149. PL decay of **1(2:3)** at 77K.

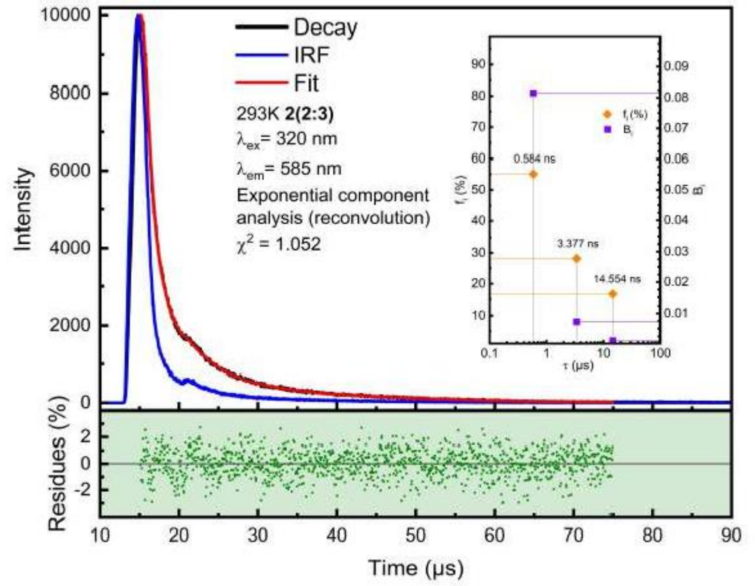

Figure S150. PL decay of **2(2:3)** at 293K.

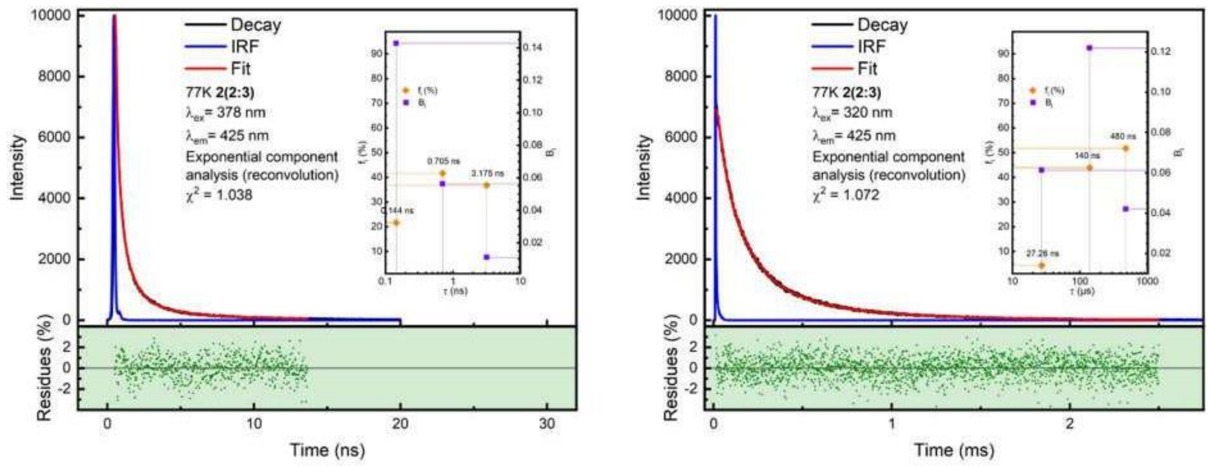

Figure S151. PL decay of **2(2:3)** at 77K. Left : short lifetimes. Right : long lifetimes.

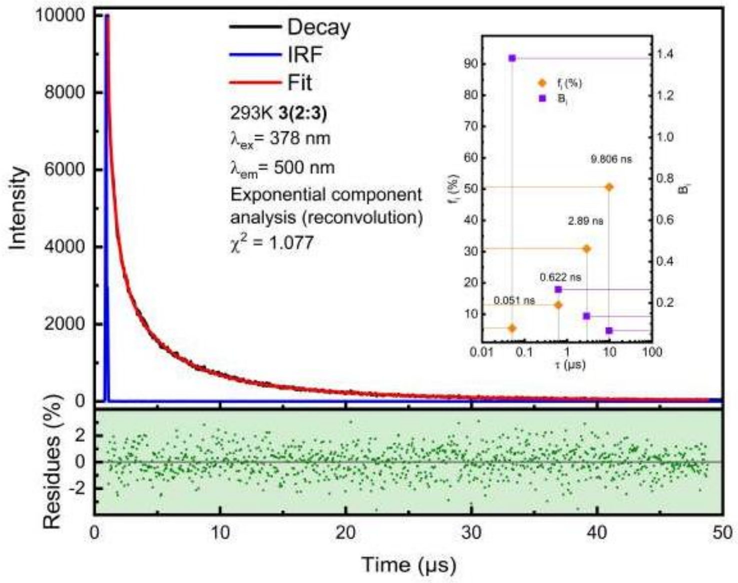

Figure S152. PL decay of **3(2:3)** at 293K.

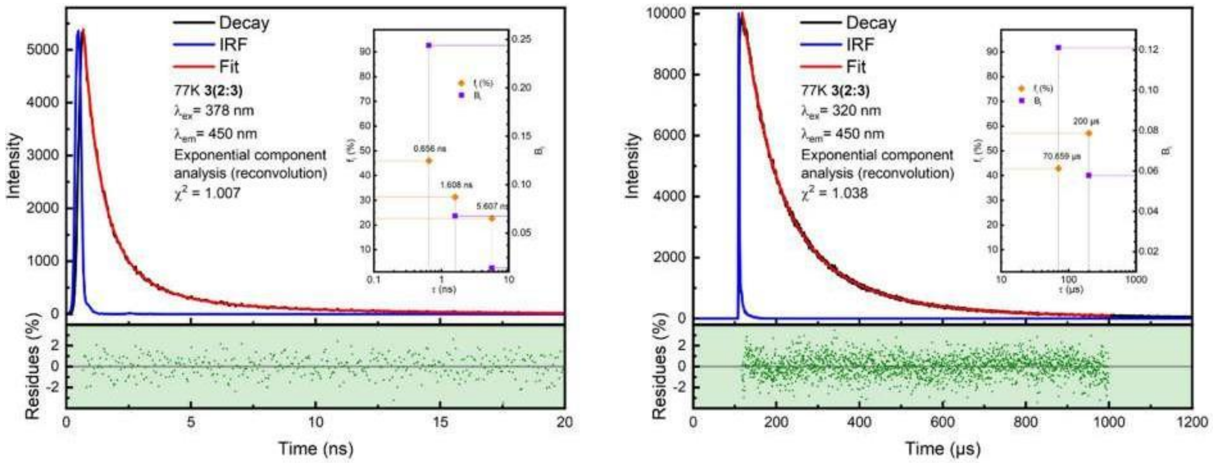

Figure S153. PL decay of **3(2:3)** at 77K.

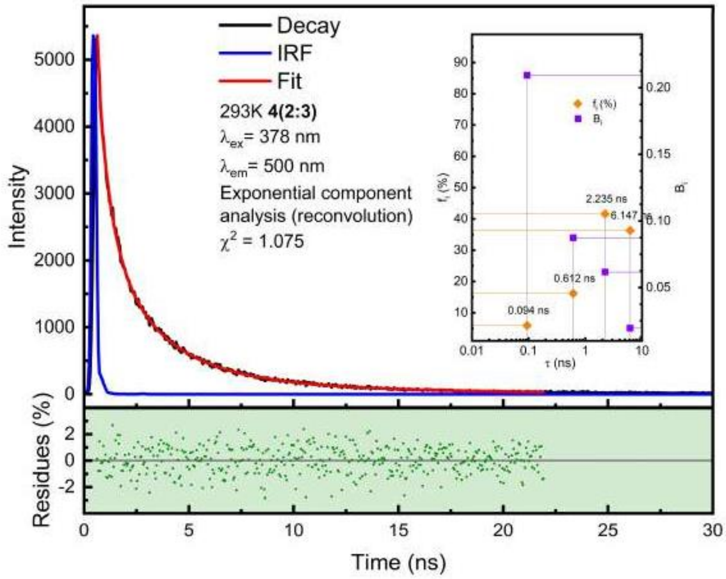

Figure S154. PL decay of **4(2:3)** at 293K.

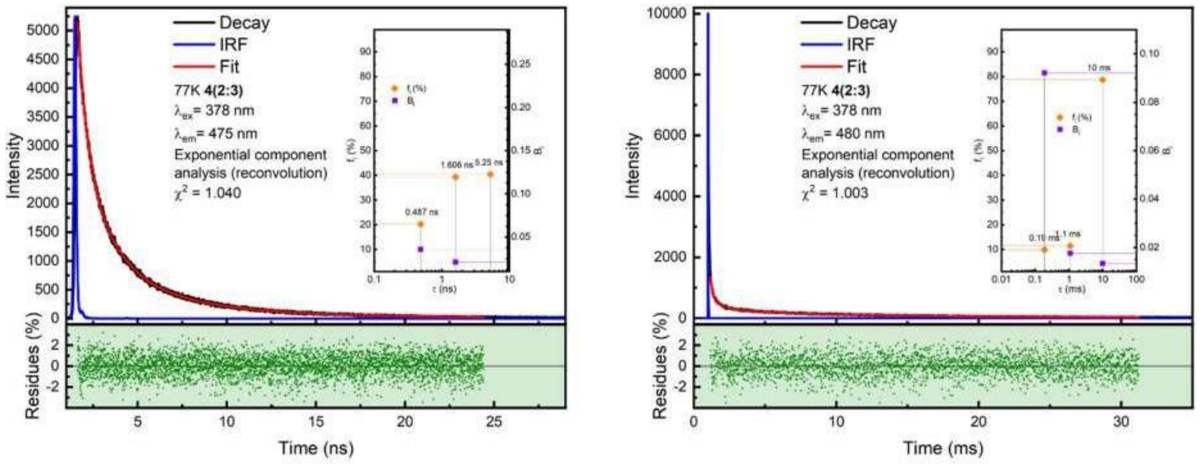

Figure S155. PL decay of **4(2:3)** at 77K. Left : short lifetimes. Right : long lifetimes.

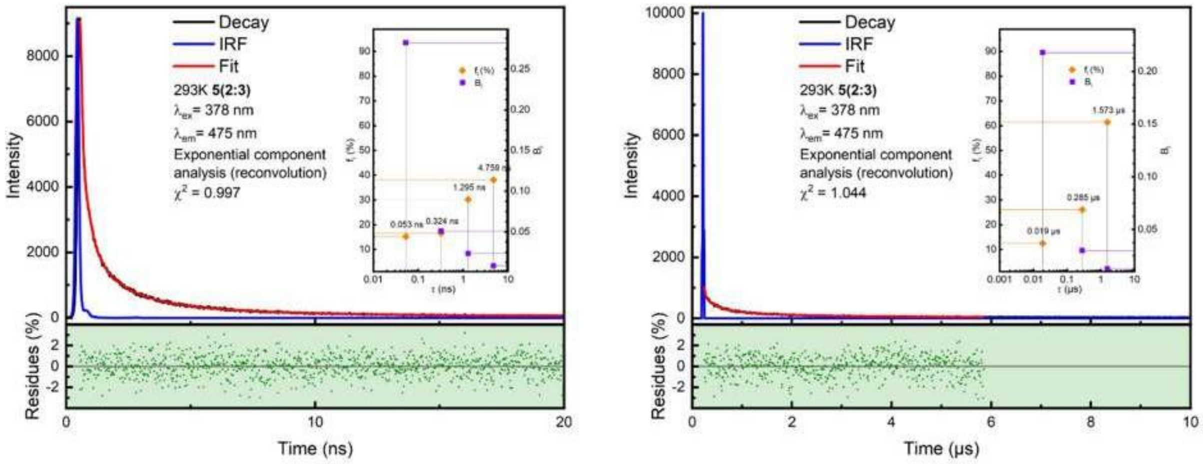

Figure S156. PL decay of **5(2:3)** at 293K. Left : short lifetimes. Right : long lifetimes.

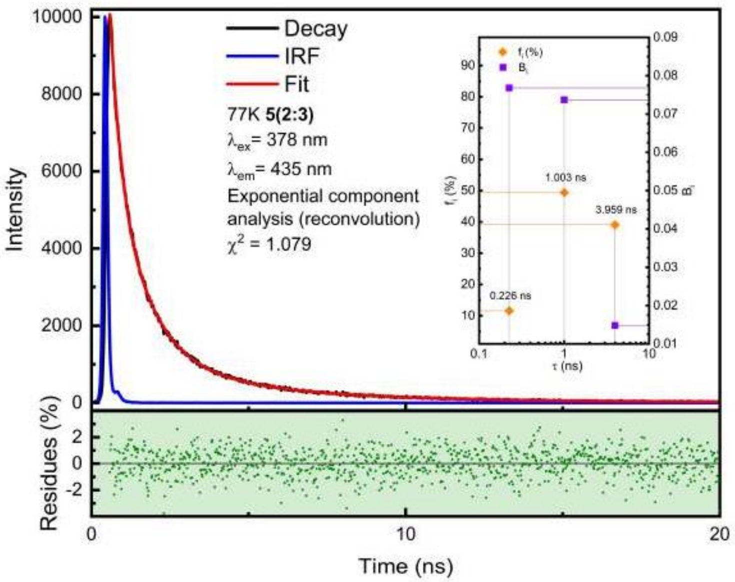

Figure S157. PL decay of **5(2:3)** at 77K.

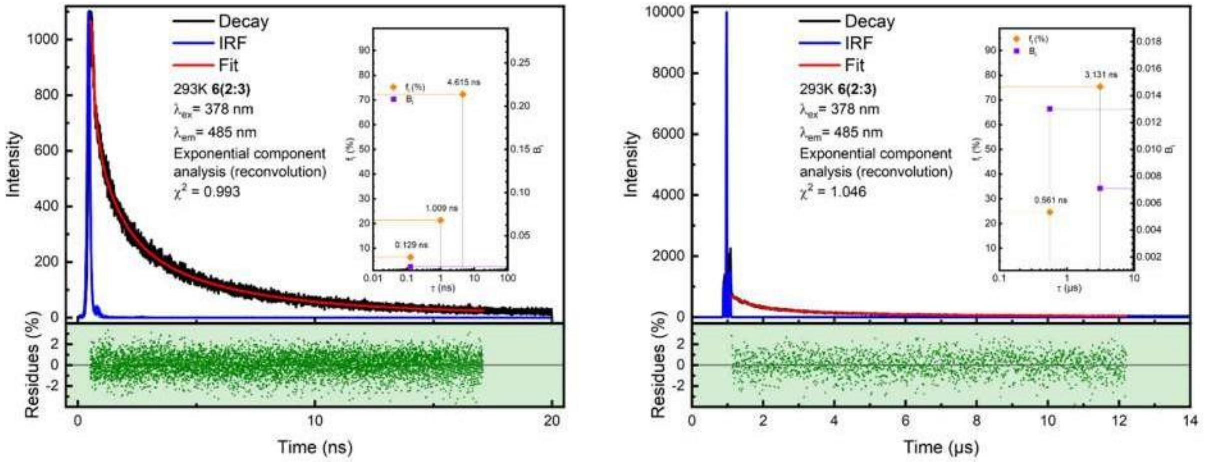

Figure S158. PL decay of **6(2:3)** at 293K. Left : short lifetimes. Right : long lifetimes.

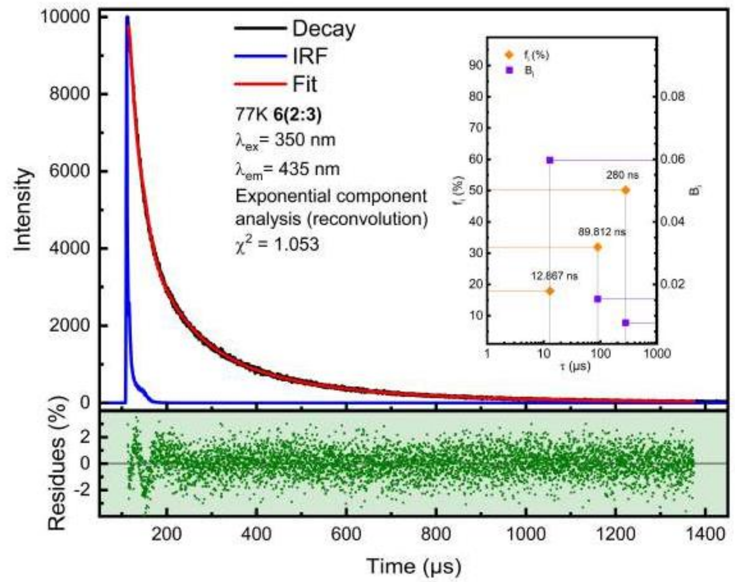

Figure S159. PL decay of **6(2:3)** at 77K.

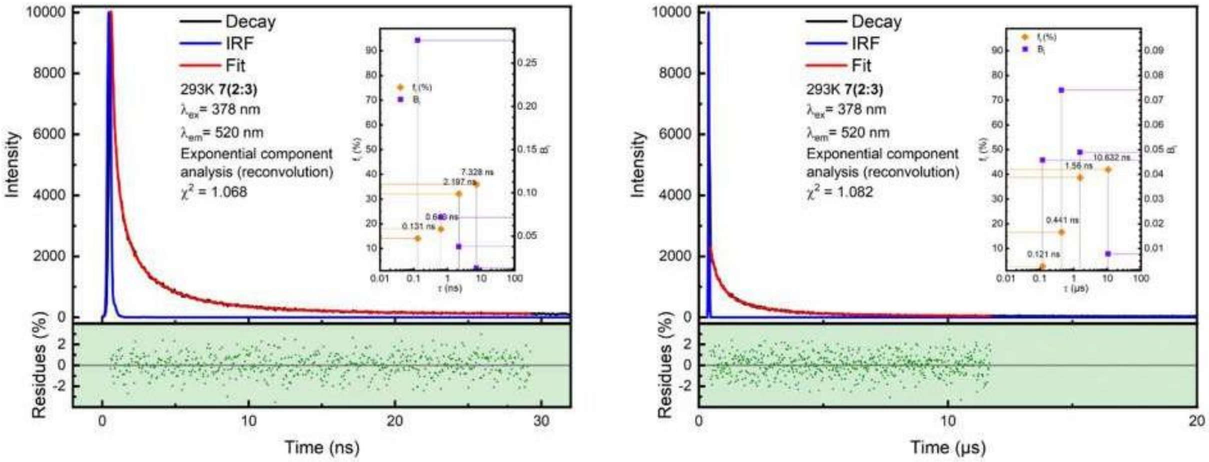

Figure S160. PL decay of **7(2:3)** at 77K. Left : short lifetimes. Right : long lifetimes.

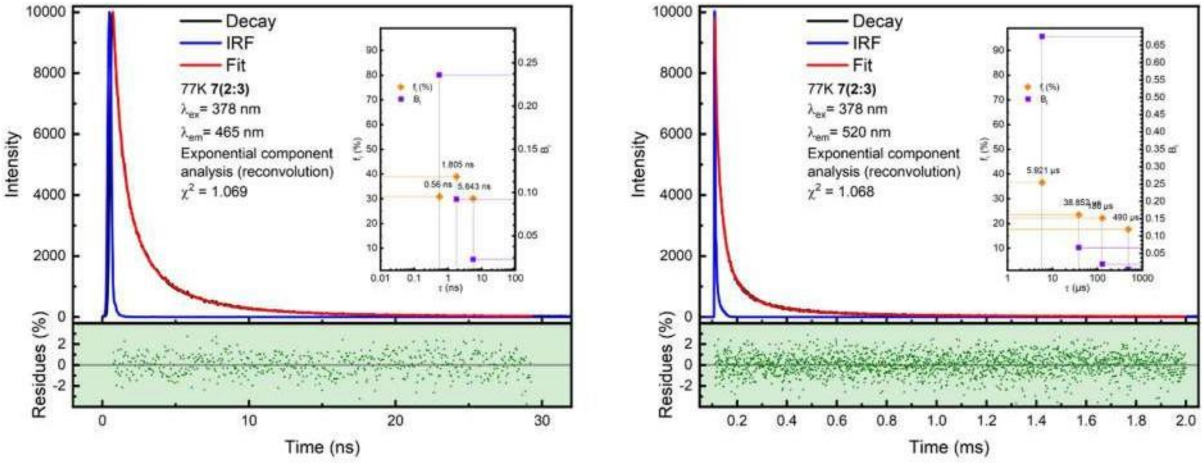

Figure S161. PL decay of **7(2:3)** at 77K. Left : short lifetimes. Right : long lifetimes.

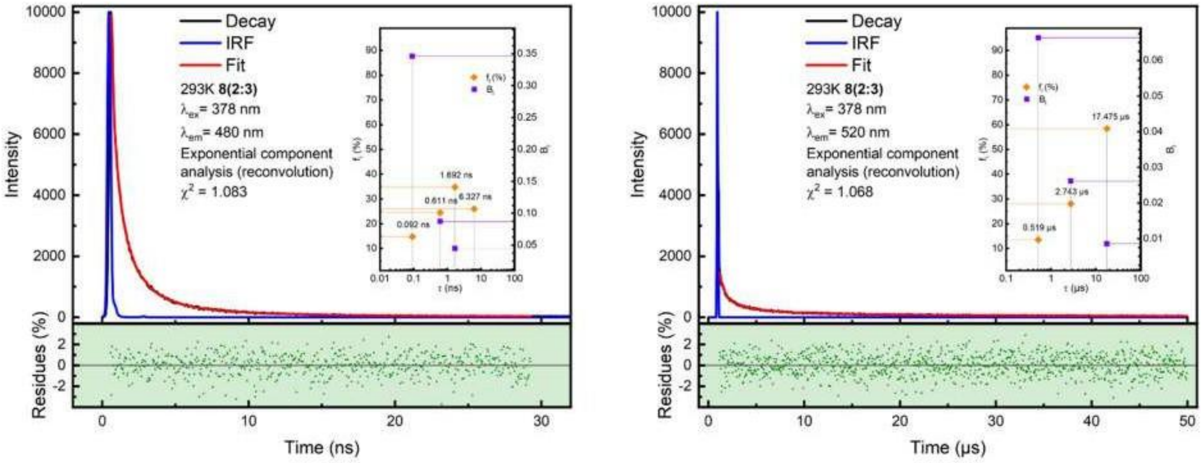

Figure S162. PL decay of **8(2:3)** at 293K. Left : short lifetimes. Right : long lifetimes.

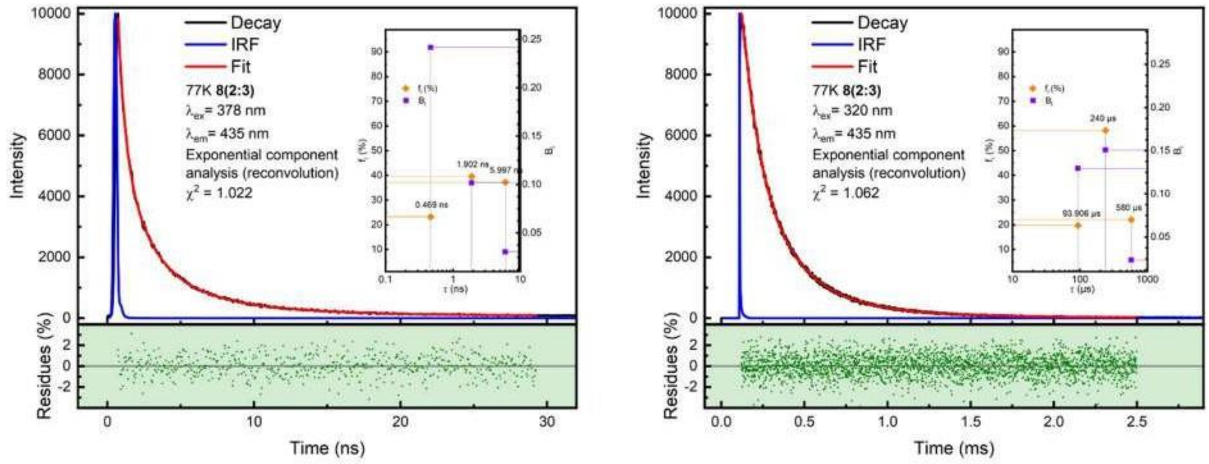

Figure S163. PL decay of **8(2:3)** at 77K. Left : short lifetimes. Right : long lifetimes.

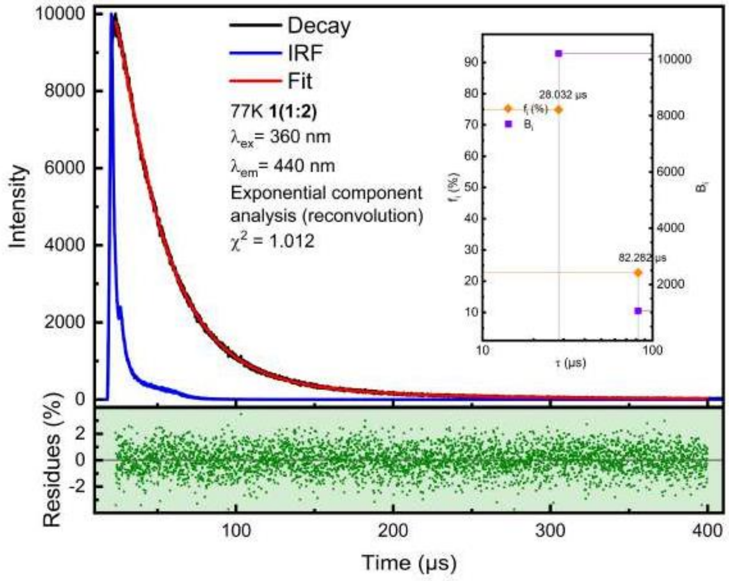

Figure S164. PL decay of **1(1:2)** at 77K.

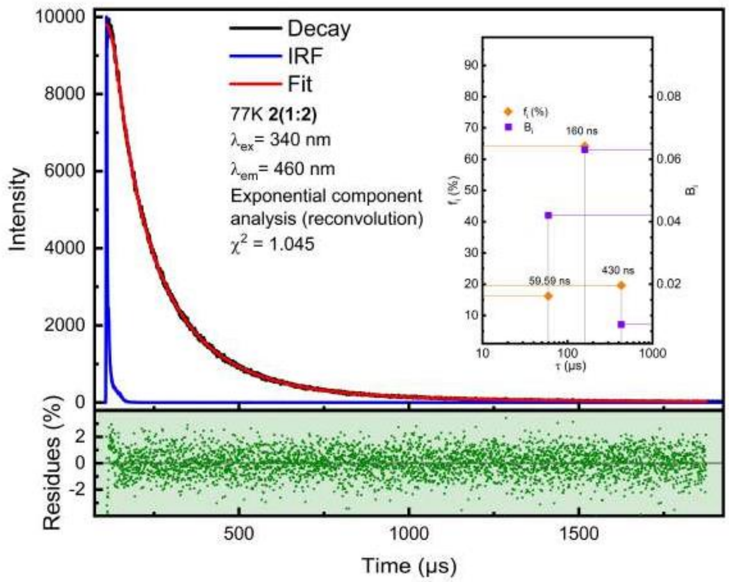

Figure S165. PL decay of **2(1:2)** at 77K.

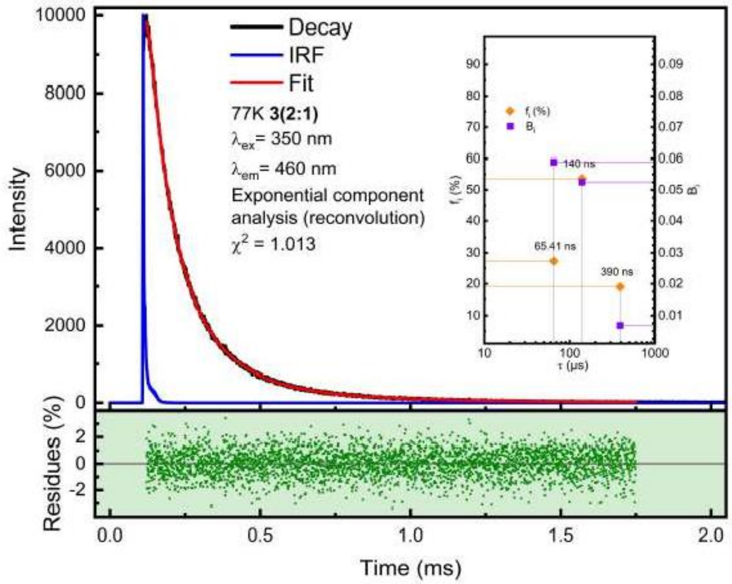

Figure S166. PL decay of **3(2:1)** at 77K.

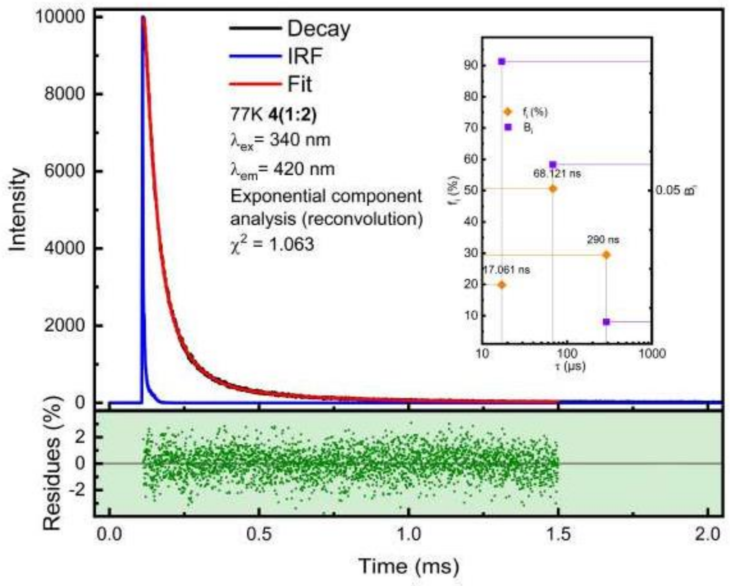

Figure S167. PL decay of **4(1:2)** at 77K.

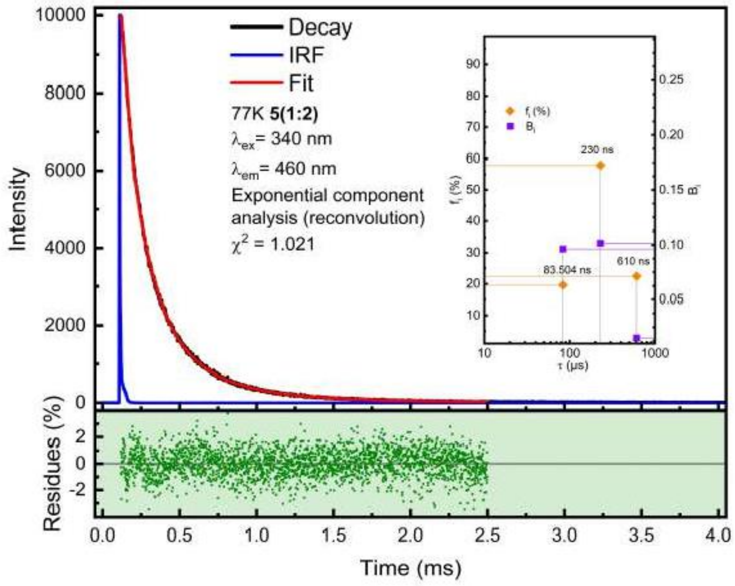

Figure S168. PL decay of **5(1:2)** at 77K.

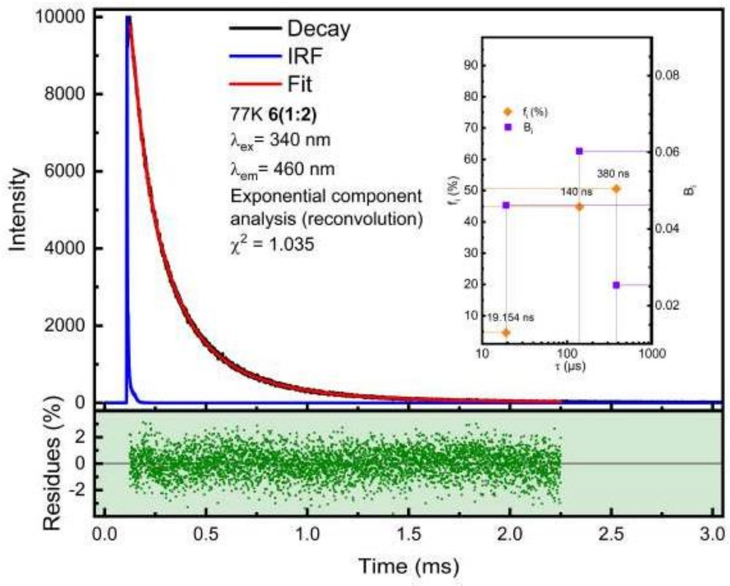

Figure S169. PL decay of **6(1:2)** at 77K.

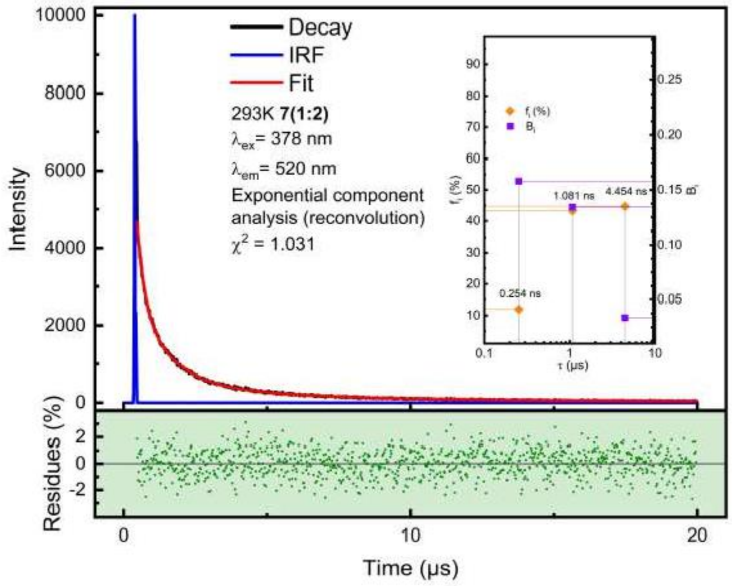

Figure S170. PL decay of **7(1:2)** at 293K.

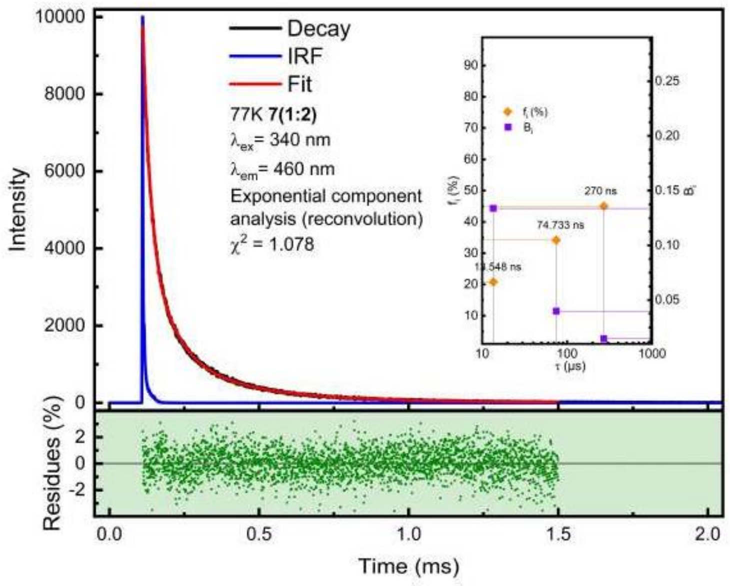

Figure S171. PL decay of **7(1:2)** at 77K.

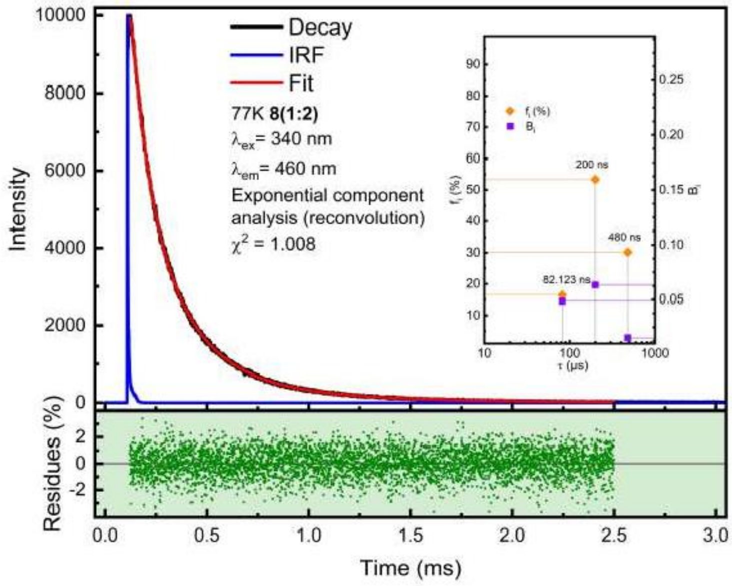

Figure S172. PL decay of **8(1:2)** at 77K.

Table S1. PL decay lifetime components for all compounds.[a]

Sample	τ1(%)	τ2(%)	τ3(%)	τ4(%)	χ^2	Notes
**1(2:1)**	1.03μs (1.64)	6.86μs (98.4)	n.a.	n.a.	1.032	293K
	366ns (28.3)	1.65μs (48.0)	6.38μs (23.7)	n.a.	1.156	77K, short lifetimes
	37.0μs (100)	n.a.	n.a.	n.a.	1.053	77K, long lifetimes
**2(2:1)**	255ns (17.7)	1.77μs (32.1)	6.14μs (50.2)	n.a.	1.367	293K
	15.5μs (26.7)	69.1μs (34.4)	270μs (38.9)	n.a.	1.000	77K
**3(2:1)**	497ns (6.43)	2.71μs (34.2)	6.37μs (59.4)	n.a.	1.100	293K
	599ns (10.3)	15.5μs (14.5)	63.3μs (41.9)	190μs (33.4)	1.074	77K
**4(2:1)**	1.73μs (49.9)	5.87μs (31.3)	42.5μs (18.8)	n.a.	1.011	293K
	7.85μs (34.2)	54.2μs (33.4)	250μs (32.4)	n.a.	1.024	77K, short lifetimes
	26.5μs (24.5)	1.6ms (13.2)	9.4ms (85.6)	n.a.	1.043	77K, long lifetimes
**5(2:1)**	5.13μs (35.9)	9.18μs (64.1)	n.a.	n.a.	1.000	293K
	20.8μs (63.1)	75.0μs (23.0)	250μs (13.8)	n.a.	1.003	77K
**6(2:1)**	[illegible]ns (5.05)	3.79μs (51.2)	7.43μs (43.8)	n.a.	1.062	293K
**7(2:1)**	1.30μs (3.79)	5.00μs (96.2)	n.a.	n.a.	1.088	293K
**8(2:1)**	1.11μs (4.28)	5.12μs (95.7)	n.a.	n.a.	1.071	293K
**1(3:2)**	178ps (25.3)	670ps (41.3)	2.50ns (33.4)	n.a.	1.068	293K, short lifetimes
	38.0ns (5.08)	330ns (14.9)	13.9μs (32.9)	49.5μs (47.0)	1.131	293K, long lifetimes

28.8µs (21.2)	86.8µs (78.8)	n.a.	n.a.	1.009	77K, short lifetimes

	62.8µs (2.18)	140µs (97.8)	n.a.	n.a.	1.071	77K, long lifetimes
**2(3:2)**	<100ps (21.8)	247ps (28.5)	1.30ns (49.7)	n.a.	1.070	293K, short lifetimes
	139ns (11.5)	704ns (27.5)	3.25µs (61.0)	n.a.	1.076	293K, long lifetimes
	8.82µs (72.7)	41.0µs (14.0)	160µs (13.3)	n.a.	1.094	77K
**3(3:2)**	611ns (4.38)	2.89µs (33.1)	6.13µs (62.5)	n.a.	1.055	293K
	9.62µs (21.5)	51.9µs (45.4)	160µs (33.1)	n.a.	1.036	77K
**4(3:2)**	6.54µs (19.3)	17.9µs (56.7)	91.3µs (24.0)	n.a.	1.011	293K
	308ps (16.1)	1.23ns (53.9)	4.85ns (30.0)	n.a.	1.069	77K, short lifetimes
	120µs (5.08)	1.6ms (8.03)	8.7ms (86.9)	n.a.	1.045	77K, long lifetimes
**5(3:2)**	2.31µs (18.5)	7.30µs (81.5)	n.a.	n.a.	1.016	293K
	19.3µs (23.0)	61.9µs (52.0)	200µs (25.0)	n.a.	1.081	77K
**6(3:2)**	705ns (2.93)	3.99µs (23.8)	8.39µs (73.3)	n.a.	1.024	293K
**7(3:2)**	6.57µs (100)	n.a.	n.a.	n.a.	0.998	293K
	1.68µs (21.7)	11.4µs (15.6)	48.3µs (24.3)	170µs (38.5)	1.011	77K
**8(3:2)**	572ns (6.26)	2.94µs (24.4)	6.22µs (69.3)	n.a.	1.005	293K
	13.3µs (16.3)	62.7µs (42.7)	180µs (41.0)	n.a.	1.039	77K
**1(1:1)**	[illegible] (1.76)	24.2µs (61.5)	67.5µs (36.7)	n.a.	1.029	77K
**2(1:1)**	51.0ns (30.7)	903ns (29.8)	4.24µs (39.5)	n.a.	1.095	293K
	8.41µs (21.7)	53.6µs (33.2)	170µs (45.1)	n.a.	1.033	77K
**3(1:1)**	[illegible] (11.9)	1.58µs (33.00	6.05µs (55.1)	n.a.	1.036	293K

	12.0μs (23.3)	54.7μs (46.8)	160μs (29.9)	n.a.	1.051	77K
**4(1:1)**	36.9μs (22.2)	110μs (47.3)	380μs (30.6)	n.a.	1.059	77K
**5(1:1)**	127ps (8.96)	982ps (64.2)	2.25ns (26.8)	n.a.	0.993	293K, short lifetimes
	199ns (18.3)	1.39μs (81.7)	n.a.	n.a.	1.056	293K, long lifetimes
	38.3μs (25.4)	130μs (39.5)	380μs (35.1)	n.a.	1.012	77K
**6(1:1)**	294ns (20.1)	55.8μs (49.6)	110μs (30.3)	n.a.	1.036	77K
**7(1:1)**	158ps (14.0)	942ps (28.0)	3.42ns (58.0)	n.a.	1.049	293K, short lifetimes
	292ns (4.59)	1.47μs (26.0)	5.09μs (69.4)	n.a.	1.006	293K, long lifetimes
	529ps (46.5)	1.05ns (45.2)	4.00ns (8.27)	n.a.	0.994	77K, short lifetimes
	9.99μs (25.1)	44.5μs (38.4)	130μs (36.6)	n.a.	1.059	77K, long lifetimes
**8(1:1)**	23.4μs (13.4)	96.3μs (34.1)	310μs (52.4)	n.a.	0.991	77K
**1(2:3)**	205ns (10.6)	896ns (35.8)	3.23μs (53.5)	n.a.	1.010	293K
	7.88μs (23.3)	46.1μs (38.4)	190μs (38.3)	n.a.	1.049	77K
**2(2:3)**	584ns (55.1)	3.38μs (28.2)	14.6μs (16.7)	n.a.	1.052	293K
	144ps (21.6)	705ps (41.6)	3.18ns (36.7)	n.a.	1.038	77K, short lifetimes
	27.3μs (4.31)	140μs (43.9)	480μs (51.8)	n.a.	1.072	77K, long lifetimes
**3(2:3)**	51.0ns (5.50)	622ns (12.9)	2.89μs (30.9)	9.81μs (50.7)	1.077	293K
	656ps (46.0)	1.61ns (31.3)	5.61ns (22.7)	n.a.	1.007	77K, short lifetimes
	70.9μs (42.9)	200μs (57.1)	n.a.	n.a.	1.038	77K, long lifetimes

**4(2:3)**	<100ps (5.94)	612ps (16.2)	2.24ns (41.6)	6.15ns (36.3)	1.075	293K

	487ps (20.3)	1.61ns (39.3)	5.25ns (40.4)	n.a.	1.040	77K, short lifetimes
	190µs (9.86)	1.10ms (11.4)	10.0ms (78.7)	n.a.	1.003	77K, long lifetimes
**5(2:3)**	<100ps (15.2)	324ps (16.6)	1.30ns (30.2)	4.76ns (38.1)	0.997	293K, short lifetimes
	19.0 ns (12.5)	285ns (26.1)	1.57µs (61.4)	n.a.	1.044	293K, long lifetimes
	226ps (11.6)	1.00ns (49.4)	3.96ns (39.0)	n.a.	1.079	77K
**6(2:3)**	129ps (6.42)	1.01ns (21.3)	4.62ns (72.3)	n.a.	0.993	293K, short lifetimes
	561ns (24.6)	3.13µs (75.4)	n.a.	n.a.	1.046	293K, long lifetimes
	12.9µs (17.9)	89.8µs (31.9)	280µs (50.1)	n.a.	1.053	77K
**7(2:3)**	131ps (14.1)	643ps (17.8)	2.20ns (32.1)	7.33ns (36.0)	1.068	293K, short lifetimes
	121ns (2.80)	441ns (16.6)	1.56µs (38.7)	10.6µs (41.9)	1.082	293K, long lifetimes
	560ps (30.9)	1.81ns (38.9)	5.64ns (30.2)	n.a.	1.069	77K, short lifetimes
	5.92µs (36.6)	38.9µs (23.6)	130µs (22.2)	490µs (17.7)	1.037	77K, long lifetimes
**8(2:3)**	<100ps (14.7)	611ps (24.5)	1.69ns (34.8)	6.33ns (26.0)	1.083	293K, short lifetimes
	519ns (13.5)	2.74µs (28.1)	17.5µs (58.4)	n.a.	1.021	293K, long lifetimes
	469ps (23.2)	1.90ns (39.6)	6.00ns (37.2)	n.a.	1.022	77K, short lifetimes
	93.9µs (19.7)	240µs (58.2)	580µs (22.0)	n.a.	1.062	77K, long lifetimes
**1(1:2)**	6.25µs (2.42)	28.0µs (74.9)	82.3µs (22.7)	n.a.	1.012	77K
**2(1:2)**	59.6µs (16.2)	160µs (64.2)	430µs (19.6)	n.a.	1.045	77K
**3(1:2)**	65.4µs (27.3)	140µs (53.6)	390µs (19.0)			
**4(1:2)**	17.1µs (19.9)	68.1µs (50.7)	290µs (29.5)			

n.a.	1.013	77K
n.a.	1.063	77K

**5(1:2)**	83.5μs (19.7)	230μs (57.9)	610μs (22.5)	n.a.	1.021	77K
**6(1:2)**	19.2μs (4.59)	140μs (44.8)	380μs (50.6)	n.a.	1.035	77K
**7(1:2)**	254ns (12.0)	1.08μs (43.3)	4.45μs (44.7)	n.a.	1.031	293K
	13.5μs (20.8)	74.7μs (34.2)	270μs (45.0)	n.a.	1.078	77K
**8(1:2)**	82.1μs (16.5)	200μs (53.4)	480μs (30.2)	n.a.	1.008	77K

[a]Some percentage totals may not be equal to 100%, due to rounding error.

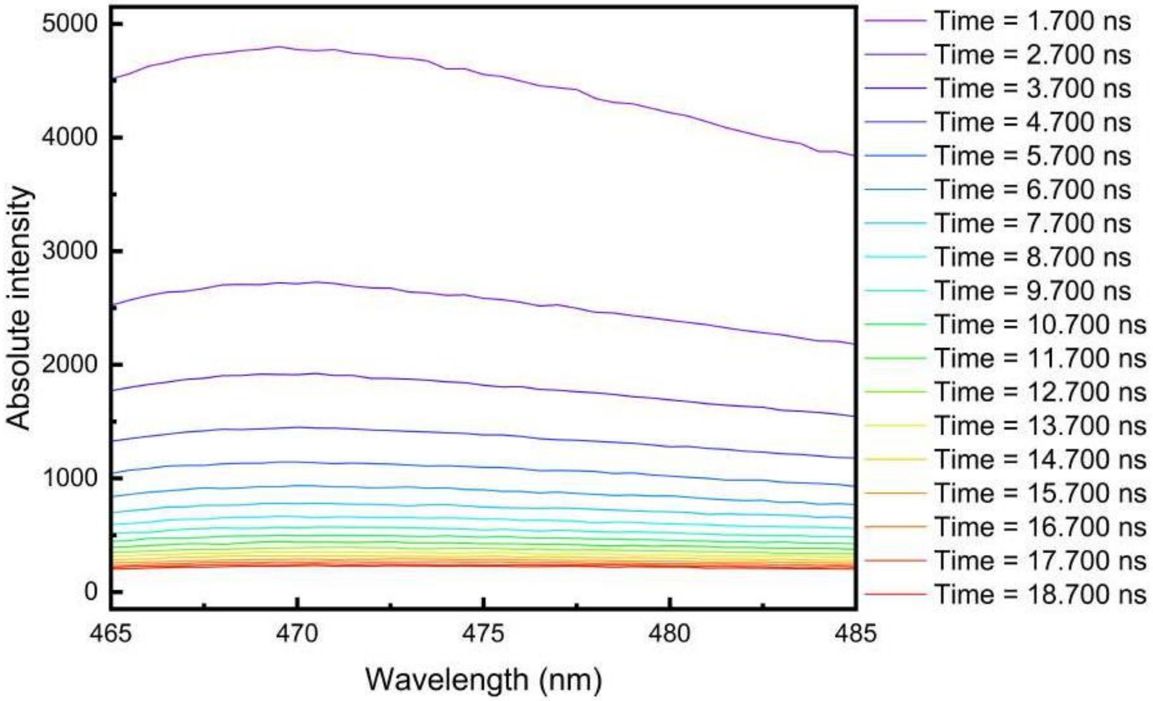

Figure S173. Absolute time-resolved emission spectra of **5(1:1)** at 293K. λ_{ex} = 378nm.

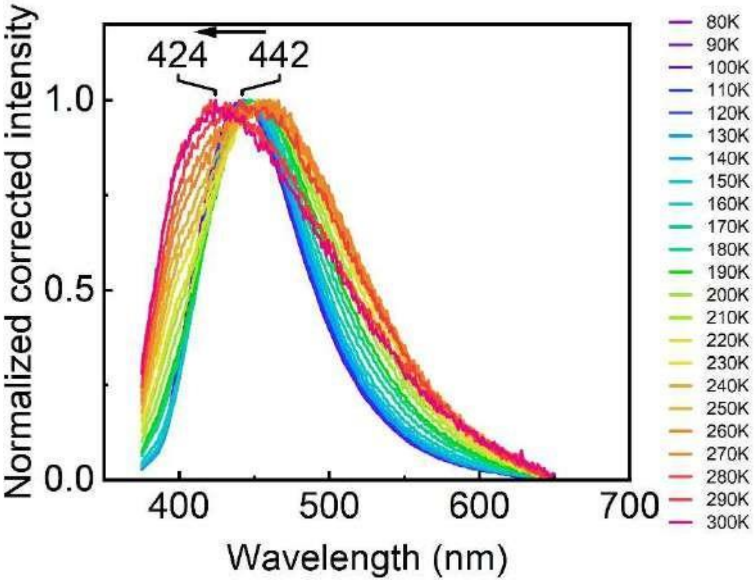

Figure S174. Normalized emission spectra of **5(1:1)** dependent on the temperature.

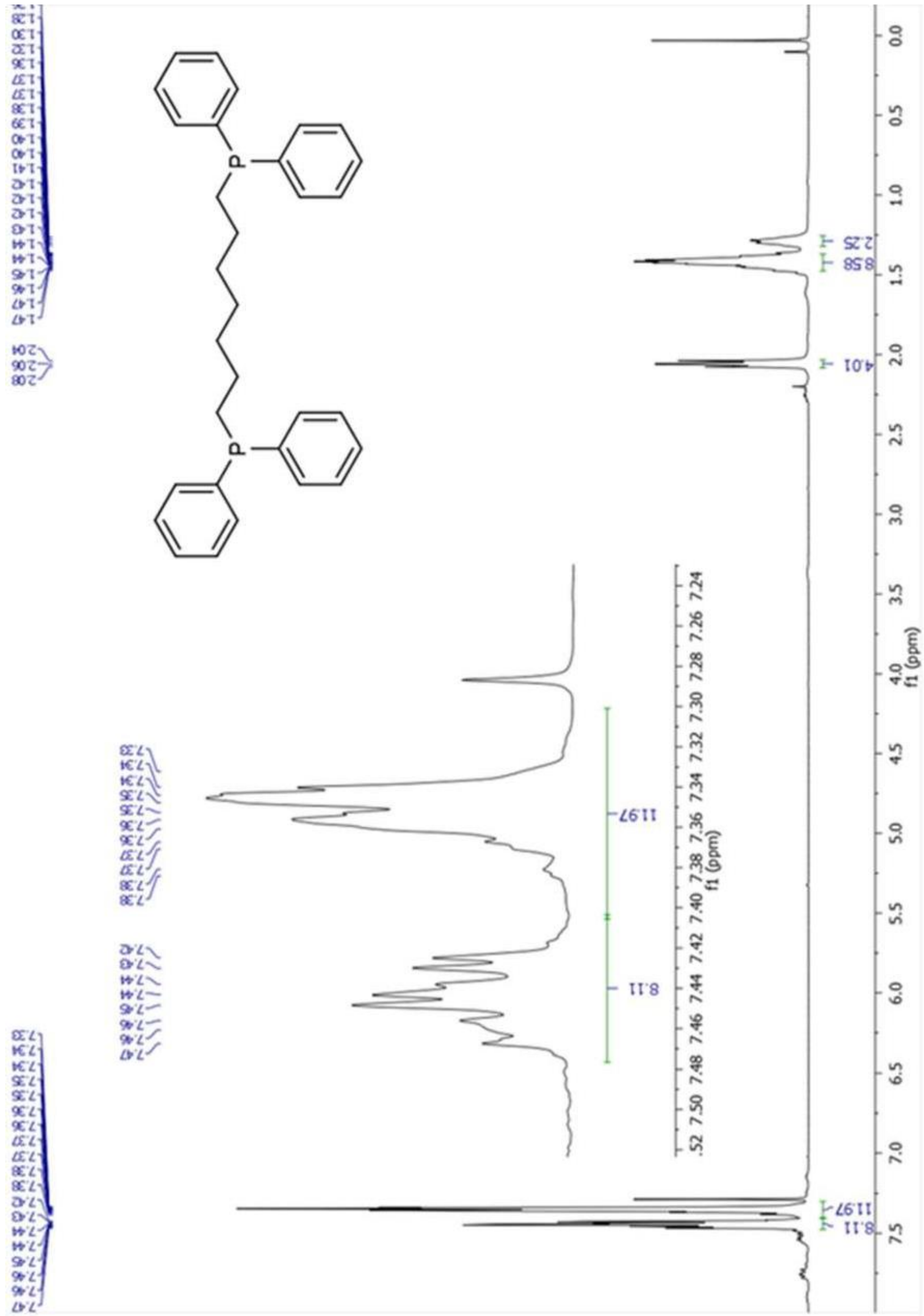

Figure S175. 400 MHz ^{1}H NMR spectrum of **L7** in $CDCl_3$.

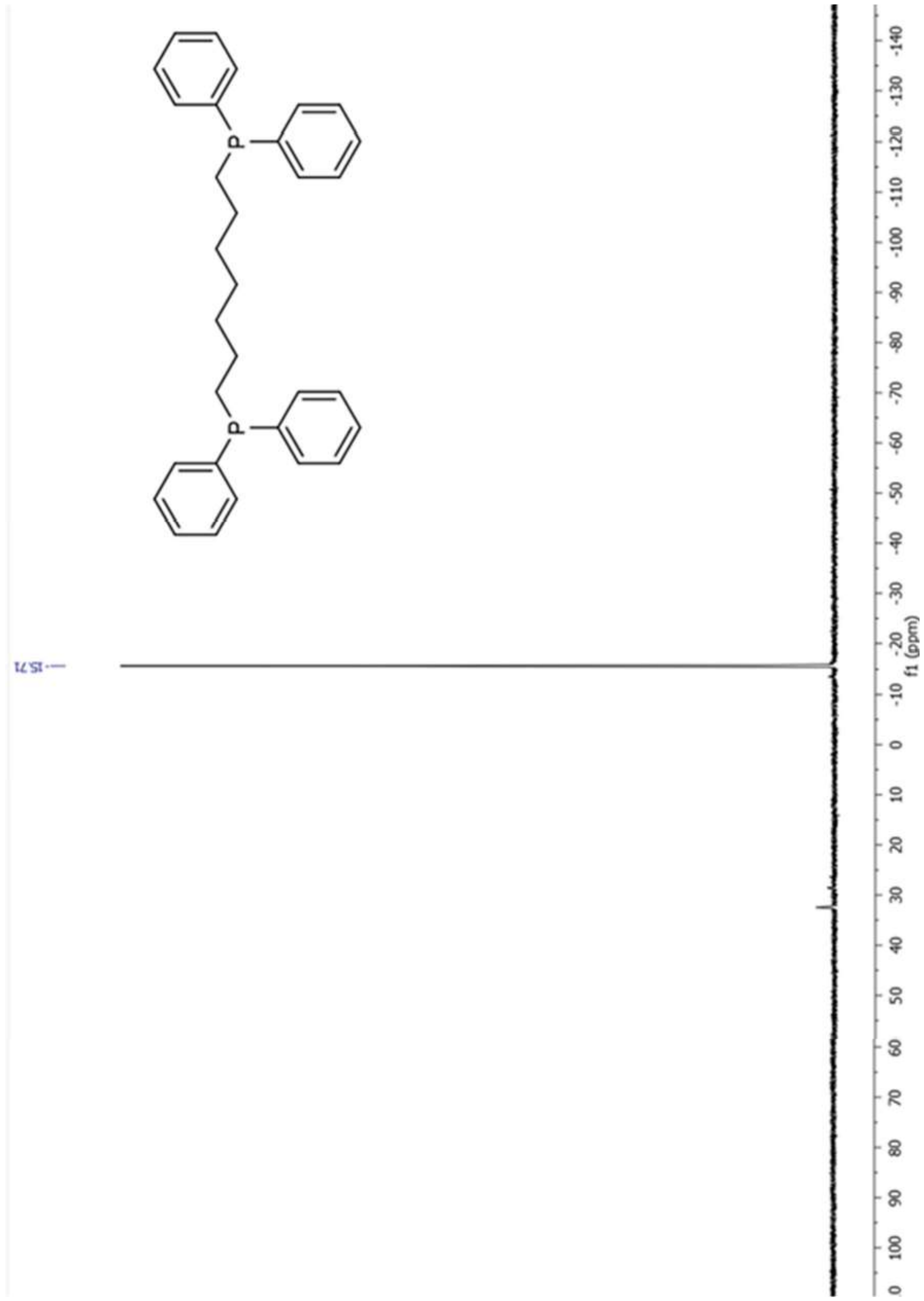

Figure S176. 162 MHz ^{31}P NMR spectrum of **L7** in $CDCl_3$.

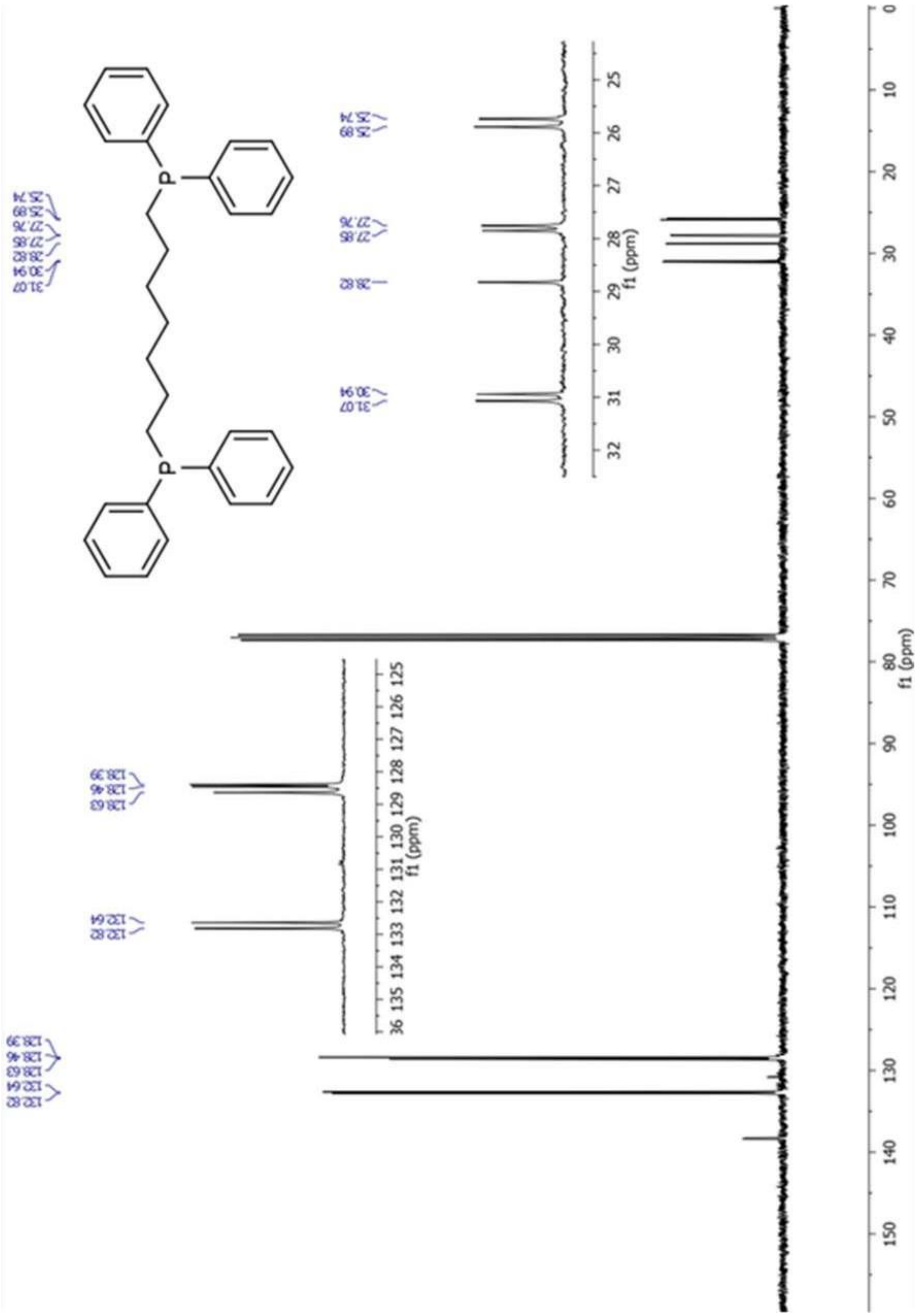

Figure S177. 100 MHz ^{13}C NMR spectrum of **L7** in $CDCl_3$.

REFERENCES

(1) Marsich, N.; Nardin, G.; Randaccio, L. Tetranuclear Copper(I) Complex. Crystal and Molecular Structure of the 2:1 Derivative of Copper(I) Iodide and Bis(Diphenylphosphino)Methane. *J Am Chem Soc* **1973**, *95* (12), 4053–4054. https://doi.org/10.1021/ja00793a040.

(2) Camus, A.; Nardin, G.; Randaccio, L. Structure Determination of the 2:1 Derivatives of Copper(I) Bromide and Iodide with Bis(Diphenylphosphino)Methane. A Simple Structural Scheme for the Formation of (CuX)NLm Species. *Inorganica Chim Acta* **1975**, *12* (1), 23–32. https://doi.org/10.1016/S0020-1693(00)89831-X.

(3) Benito, Q.; Le Goff, X. F.; Nocton, G.; Fargues, A.; Garcia, A.; Berhault, A.; Kahlal, S.; Saillard, J.-Y.; Martineau, C.; Trébosc, J.; Gacoin, T.; Boilot, J.-P.; Perruchas, S. Geometry Flexibility of Copper Iodide Clusters: Variability in Luminescence Thermochromism. *Inorg Chem* **2015**, *54* (9), 4483–4494. https://doi.org/10.1021/acs.inorgchem.5b00321.

(4) Qian, H.; Chen, Y.; Ren, Y.; Zhang, Y.; Lang, J. Solvothermal Synthesis and Crystal Structure of Cu2 (μ-Dppb)2 (μ-I)2 (Dppb = 1,4-Di(Diphenylphosphine)Butane). *Journal of Suzhou University (Natural Science Edition)* **2008**, *24* (4), 83–86.

(5) Zhang, X.; Song, L.; Hong, M.; Shi, H.; Xu, K.; Lin, Q.; Zhao, Y.; Tian, Y.; Sun, J.; Shu, K.; Chai, W. Luminescent Dinuclear Copper(I) Halide Complexes Double Bridged by Diphosphine Ligands: Synthesis, Structure Characterization, Properties and TD-DFT Calculations. *Polyhedron* **2014**, *81*, 687–694. https://doi.org/10.1016/j.poly.2014.07.034.

(6) Effendy; Di Nicola, C.; Fianchini, M.; Pettinari, C.; Skelton, B. W.; Somers, N.; White, A. H. The Structural Definition of Adducts of Stoichiometry MX:Dppx (1:1) M=CuI, AgI, X=simple Anion, Dppx=Ph2P(CH2)XPPh2, X=3–6. *Inorganica Chim Acta* **2005**, *358* (3), 763–795. https://doi.org/10.1016/j.ica.2004.09.047.

(7) Nardin, G.; Randaccio, L.; Zangrando, E. Stereochemistry of Copper(I) Complexes. Part II. The Molecular Structure of the 3 : 2 Reaction Product between Copper Iodide and Bis(Diphenylphosphino)Methane: Di-μ-[Bis(Diphenylphosphino)Methane]-μ-Iodo-Di-M3-Iodo-Triangulo-Tricopper(I)–0.5 Dichloromethane. *Journal of the Chemical Society, Dalton Transactions* **1975**, No. 23, 2566. https://doi.org/10.1039/dt9750002566.

(8) Aslanidis, P.; Cox, P. J.; Divanidis, S.; Tsipis, A. C. Copper(I) Halide Complexes with 1,3-Propanebis(Diphenylphosphine) and Heterocyclic Thione Ligands: Crystal and Electronic Structures (DFT) of [CuCl(PymtH)(Dppp)], [CuBr(PymtH)(Dppp)], and [Cu(*μ* -I)(Dppp)]$_2$. *Inorg Chem* **2002**, *41* (25), 6875–6886. https://doi.org/10.1021/ic025896i.

(9) Effendy; di Nicola, C.; Pettinari, C.; Pizzabiocca, A.; Skelton, B. W.; Somers, N.; White, A. H. The Structural Definition of Adducts of Stoichiometry MX:Dpex (2:3)(∞), M=CuI, AgI, X=simple Anion, Dpex=Ph2E(CH2)XEPh2, E=P, As. *Inorganica Chim Acta* **2006**, *359* (1), 64–80. https://doi.org/10.1016/j.ica.2005.06.012.

(10) Comba, P.; Katsichtis, C.; Nuber, B.; Pritzkow, H. Solid-State and Solution Structural Properties of Copper(I) Compounds with Bidentate Phosphane Ligands. *Eur J Inorg Chem* **1999**, *1999* (5), 777–783. https://doi.org/10.1002/(SICI)1099-0682(199905)1999:5<777::AID-EJIC777>3.0.CO;2-A.
